# Electric field-dependent excited-state twisting in donor–acceptor thiazolothiazole dyes establishes a new class of cell membrane voltage sensors

**DOI:** 10.1039/d6sc04210d

**Published:** 2026-08-25

**Authors:** Pranamita Chakraborti, Rui Fu, Lucia M. Wert, Charlie A. L. Darby, Joy D. Amuzu, Daphne Oettinger, Aditya Nandy, Yamuna Krishnan, Michael G. Walter

**Affiliations:** a Department of Chemistry, University of North Carolina at Charlotte Charlotte NC 28223 USA Michael.Walter@charlotte.edu; b Department of Chemistry, The University of Chicago Chicago IL 60637 USA yamuna@uchicago.edu; c Neuroscience Institute, The University of Chicago Chicago IL 60637 USA; d Institute of Biophysical Dynamics, The University of Chicago Chicago IL 60637 USA; e Department of Chemical and Biomolecular Engineering, University of California, Los Angeles Los Angeles CA 90095 USA aditya.nandy@ucla.edu; f Immunoengineering and Bioengineering Institute, The University of Chicago USA

## Abstract

Voltage sensitive dyes (VSDs) are versatile and powerful reporters of the membrane potential across cell membranes. Thiazolothiazole (TTz) dyes are a promising new class of VSDs with high photostability and low cytotoxicity. However, the mechanistic basis of their voltage sensitivity is unknown. To address this, we developed a new generation of asymmetrically substituted TTz dyes (asym-TTz). They exhibit strong solvatofluorochromism with large Stokes shifts and exceptionally large changes in dipole moments (23–29 D), representing an 80% increase over those of previously reported TTz VSDs. An asym-TTz derivative, TwistTz-P, bearing a carboxyphenyl acceptor group, shows sustained cellular membrane localization and a voltage sensitivity of ∼10% Δ*F*/*F* per 100 mV. Computational analysis of TwistTz-P under varying electric fields revealed a pronounced twist on both sides of the TTz bridging unit in the excited state. The degree of twisting was voltage-dependent, exhibiting an increase upon depolarization, consistent with the experimentally observed fluorescence decrease at an identical voltage. In another asym-TTz dye (FlatTz-P), where a carboxypyrazinyl acceptor group was incorporated, excited-state twisting under varying electric fields was negligible and demonstrated no voltage sensitivity. These observations establish electric field-dependent excited-state twisting as a new voltage sensing mechanism, and, additionally, provide a new strategy for creating highly sensitive VSDs.

## Abstract 2

The underlying mechanism behind the voltage sensing ability of thiazolothiazole fluorophores has been revealed through the characterization and computational analysis of several newly developed cell membrane probes.

## Graphical abstract

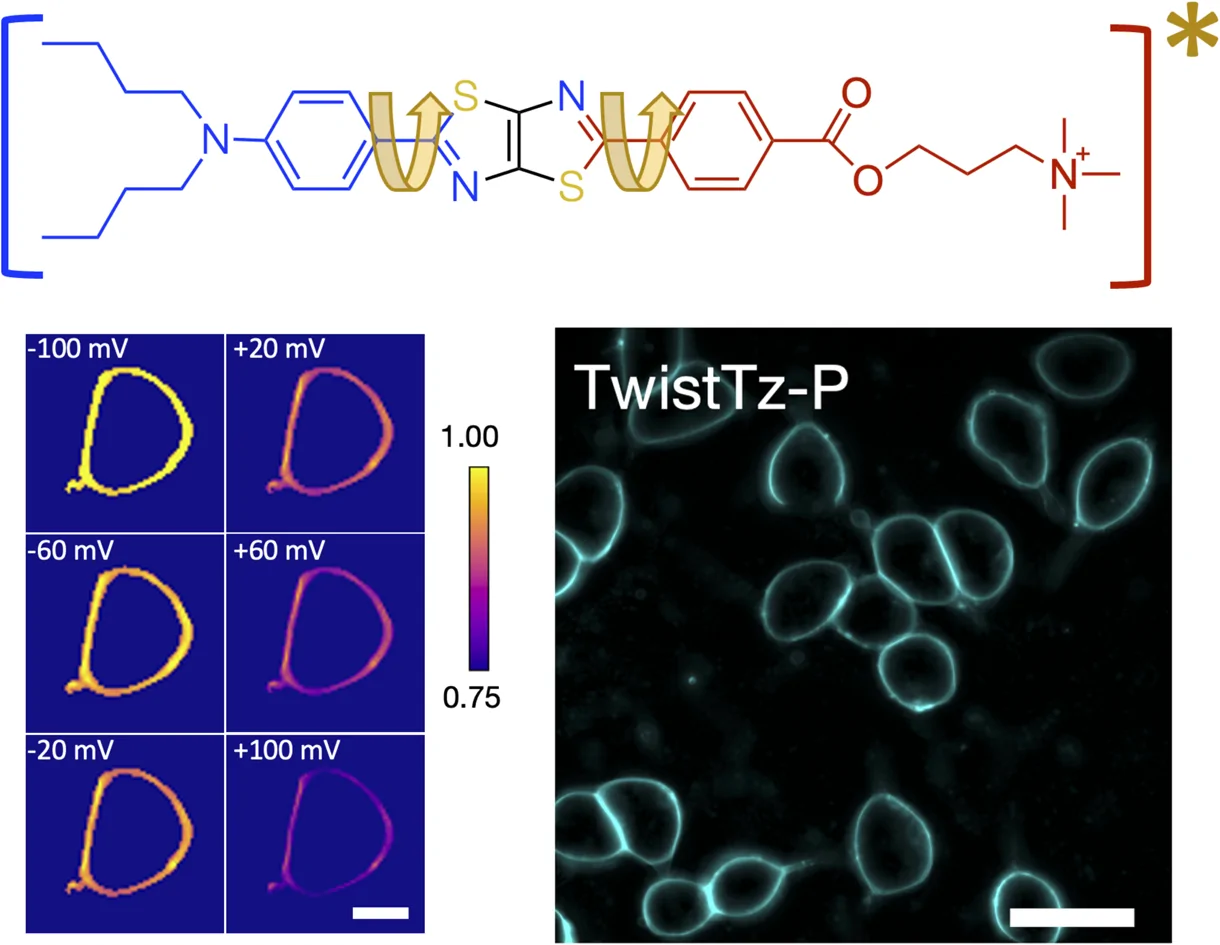

## Introduction

All biological membranes harbor a transmembrane potential, or voltage, that is pivotal to their function. This fundamental feature arises because the biological fluids they separate are highly asymmetric in terms of the concentrations of the major physiological ions, *e.g.*, Na^+^, K^+^, Ca^2+^, and Cl^−^. This sets up an electrochemical gradient across the membrane, and the opening of membrane-resident ion channels leads to the flow of specific ions, thus giving rise to a membrane potential. While the membrane potential may be measured using electrophysiology, this method is invasive and provides limited spatial information. Genetically encoded voltage indicators (GEVIs) provide spatial resolution,^1^ but are constrained by lower brightness and fast photobleaching, which impact long-term monitoring of transmembrane-voltage dynamics.^2^ Hence, there has been a renewed focus on developing voltage sensing dyes (VSDs) that use diverse mechanisms such as electrochromism, PeT, FRET, AIE and ESIPT, to fluorescently report on membrane potential.^3–7^ Commercially available voltage-sensitive dyes (VSDs) typically require fluorescence sensitivities of 10–20% Δ*F*/*F* per 100 mV to enable reliable measurements in applications such as action potential recording, drug screening, and neuronal activity mapping. The FRET dyes, although very sensitive (100–300% Δ*F*/*F* per 100 mV in isolated cells),^5^ exhibit slow response times (in the µs to ms range)^5,8^ and induce capacitive load on the membrane.^9^ Fast response dyes such as electrochromic dyes (with response times in the fs to ps range),^10^*e.g.* the ANEPPS family of dyes, often have lower voltage sensitivity (between 3 and 15% Δ*F*/*F* per 100 mV).^11^ VoltageFluor (VF) dyes sense voltage through photo-induced electron transfer (PeT), which involves fluorescence quenching *via* a molecular wire.^9^ While they have good sensitivity (>60% Δ*F*/*F* per 100 mV in some cases), fast response times (in the ns range), and negligible capacitive load,^9,12–15^ they have less-than-ideal synthetic methods and photophysical properties.

We have recently developed a new class of donor–acceptor VSDs incorporating a thiazolo[5,4-*d*]thiazole (TTz) π-conjugated molecular bridge.^16^ The first generation of asymmetric thiazolo[5,4-*d*]thiazoles or asym-TTz dyes show voltage sensitivity, high photostability, large excited-state dipoles, stable membrane localization, and fast response.^16^ Though they perform favorably compared to other VSDs, the mechanism of voltage sensing in TTz dyes is still unknown. TTz dyes have been developed for a variety of applications, including more recently, volatile organic vapor sensing and solid-state photophysical modulation of crystalline blends,^16–21^ or even as photosensitizers in photodynamic inactivation.^22^ The TTz core minimizes steric effects and reinforces planarity, thus facilitating intramolecular charge transfer readily across the molecule.^23–26^

**Fig. 1:**
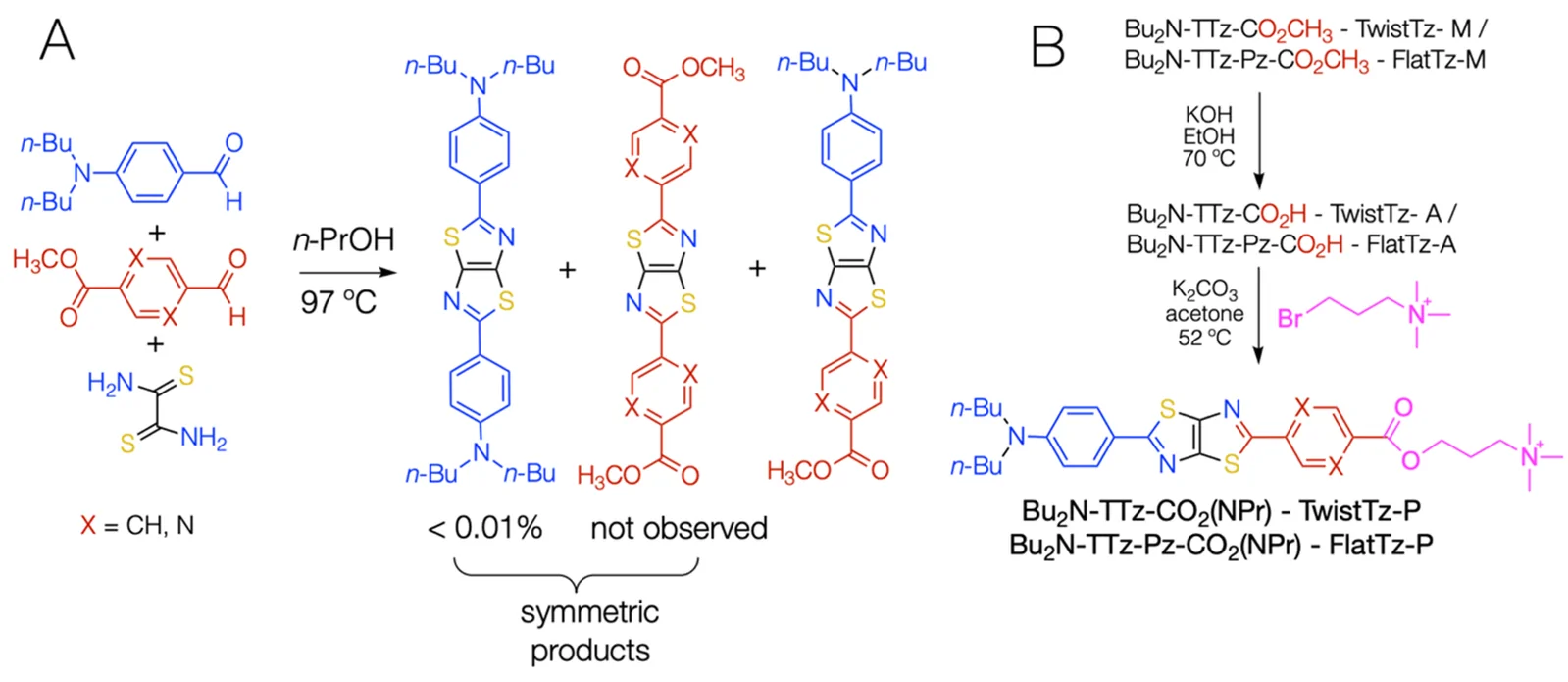
(A) Mixed substituent dibutylaminophenyl-carboxyphenyl/carboxypyrazinyl asymmetric thiazolo[5,4-*d*]thiazole (asym-TTz) derivative (TwistTz-M/FlatTz-M) accessed exclusively. (B) Sequential synthesis of TwistTz-P/FlatTz-P.

We hypothesized that the large change in dipole of asym-TTz dyes could form the basis of their observed voltage sensitivity. To test this hypothesis, we designed new push–pull asym-TTz derivatives incorporating stronger electron-acceptor groups, with the goal of enhancing their change in dipole moment.^16^ Because the first generation of asym-TTz VSDs suffered from low synthetic yields and elaborate purification steps, it limited further synthetic transformations and mechanistic investigations. Therefore, we designed asym-TTz dyes consisting of electron-donating (dibutylaminophenyl) and electron-withdrawing (carboxyphenyl or carboxypyrazinyl) groups on either side of the planar, π-conjugated aromatic TTz bridge that could be accessed *via* a one-pot synthesis. These new push–pull asym-TTz dyes could be obtained almost exclusively from a mixed-substituent reaction, thus eliminating the need for any further purification – a major improvement over the previous generation of asym-TTzs (Fig. 1). The new asym-TTzs also showed an almost ∼80% higher excited-state dipole moment compared to the first generation of asym-TTz dyes.^16^

One of these asym-TTz dyes, TwistTz-P, showed high fluorescence quantum yields, excellent cell membrane localization, and voltage sensitivity, comparing favorably to existing VSDs and to previous asym-TTz dyes (∼10% Δ*F*/*F* every 100 mV). This new class of dyes showed a gradual fluorescence decrease while going from −100 mV to + 100 mV.^16^ Supported by computational studies of excited states, followed by experimental validation, we establish a new mechanism by which small molecule fluorophores can sense voltage. Depending on the applied membrane potential, the unique, heterobicyclic TTz core can exhibit both twisted and planar excited states. By characterizing two different asym-TTz dyes that exhibit opposing twisting behaviors, we demonstrate that an electric field-induced conformational twist in the excited state is responsible for voltage sensitive fluorescence. This new mechanism of voltage sensing opens up the possibility to realize newer classes of VSDs for biological and environmental sensing strategies and applications.

## Results and discussion

**Fig. 2:**
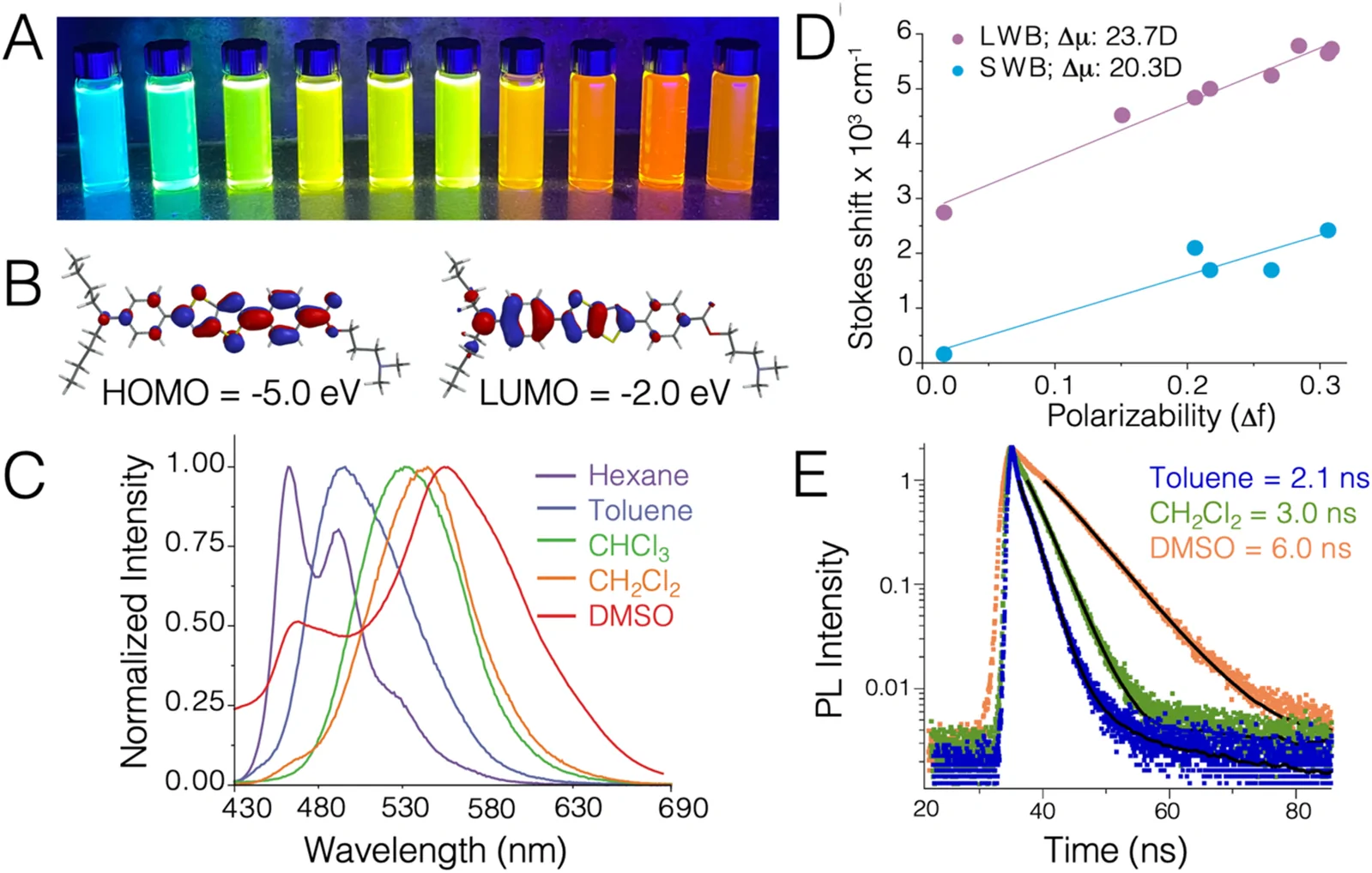
(A) Solvatofluorochromism of TwistTz-M (left to right – hexane, toluene, CHCl_3_, CH_2_Cl_2_, THF, ethyl acetate, acetone, MeCN, DMSO, MeOH). (B) Calculated HOMO/LUMO molecular orbital configurations and energies (eV) for TwistTz-P. (C) Fluorescence spectra of TwistTz-A in the indicated solvents. (D) Lippert–Mataga plots of TwistTz-P showing the long (LWB) and short wavelength band (SWB) change in dipole moments (Δ*µ*) as a function of solvent polarity (left to right – toluene, CHCl_3_, THF, CH_2_Cl_2_, DMSO, acetone, MeCN, MeOH). (E) Time-correlated single photon counting (TCSPC) fluorescence lifetimes of TwistTz-P.

We improved asym-TTz synthesis by exploring various conditions and solvent systems for the bicyclization reaction of dithiooxamide with aromatic aldehydes (Fig. 1). The synthesis is usually carried out in dimethylformamide (DMF) at high temperatures (∼150° C), which leads to product precipitation. Unfortunately, the use of high boiling point solvents like DMF can also complicate purification.^27,28^ Alcohol solvents and lower temperatures are uncommon in TTz syntheses;^29,30^ however, we examined 1-propanol as a solvent because of its lower boiling point (97 °C), easier solvent removal, and higher solubility of TTzs bearing electron-rich substituents.^31^ A molar excess of aldehydes relative to dithiooxamide (donor aldehyde : dithiooxamide : acceptor aldehyde = 1.6 : 1.45 : 1.6) was used. Products precipitated readily from the reaction mixture and were collected, filtered, and rinsed with cold 1-propanol, showing that short-chain alcohols facilitate synthesis scale-up. Of the three possible products (Fig. 1), the symmetric dicarbomethoxphenyl TTz and dibutylaminophenyl TTz were either undetected or formed in trace amounts (<0.01%). Although the overall yields were moderate for the asymmetric TTz Bu_2_N-TTz-CO_2_CH_3_ derivative, which we denote as TwistTz-M (Twisted TTz Methyl ester) (∼22%), it is highly advantageous that the asymmetric derivative is the only product formed, unlike the syntheses of the first generation of asym-TTz VSDs.^16^ To the best of our knowledge, isolation of a single product corresponding to an asymmetric TTz in a one-pot reaction has not been previously described. The TwistTz-M exhibits strong solvatofluorochromism with fluorescence Stokes shifts ranging from 0.24 eV to 0.71 eV in a range of solvents (Fig. 2A). TwistTz-M could be hydrolyzed to Bu_2_N-TTz-CO_2_H (Twisted TTz Acid or TwistTz-A). TwistTz-A was then esterified to Bu_2_N-TTz-CO_2_(NPr) (Twisted TTz Propyl ester/trimethylammonium group or TwistTz-P – Fig. 1B). TwistTz-P was designed for robust cell membrane localization and voltage sensing, because it incorporated a positively charged trimethylammonium group to orient the dye in the cell membrane, while the two *n*-butyl groups facilitated membrane anchoring.

We then studied the photophysical properties of asym-TTz derivatives in various organic solvents (SI Table S1). Asym-TTz dyes showed solvatofluorochromic emission (Fig. 2C), which is a signature of strong push–pull intramolecular charge transfer (ICT). Interestingly, the small shifts in absorption peaks (*λ*_abs_) in their spectra indicate negligible solvent effects on the ground state dipole moment. The emission spectra for TwistTz-M and TwistTz-A in hexane show clear vibrational energy levels around 460 nm and 490 nm, and a broader shoulder at 530 nm, consistent with other asym-TTzs.^16,32^ These peaks become less resolved and eventually merge as solvent polarity increases. In more polar solvents such as DMSO, asym-TTzs exhibit multiple bands arising from excited-state twisting or planarization around the dibutylamino–phenyl bond (Fig. 2C and S11, SI). The dual fluorescence includes a short wavelength band (SWB) associated with emission from a twisted, locally excited state, and a long wavelength band (LWB) associated with emission from a planar excited state.^32^ Their fluorescence lifetimes (TCSPC) depended on the polarity and the protic nature of the solvents. Increasingly polar solvents lowered the energy of the excited state with some indication of hydrogen bonding effects, given the fluorescence lifetime decrease in protic solvents.^16^ The longest fluorescence lifetime was 6.0 ns in the case of TwistTz-P in acetonitrile (Fig. 2E). With increasing solvent polarity, radiative rates decreased, while non-radiative rates increased, for all three derivatives (SI Table S1). The quantum yields were very high in non-polar solvents (*e.g. Φ*_Hex_ = 0.98 in TwistTz-M) and low in polar solvents (*e.g. Φ*_MeOH_ = 0.04 in TwistTz-M). High quantum yields in non-polar environments are desirable as this implies high fluorescence in contexts where VSDs are expected to function.^16,33^ We attribute the unexpectedly low quantum yields of TwistTz-A and TwistTz-P in certain non-polar solvents (*e.g.* hexane) to their low solubility, especially TwistTz-P, due to its positive charge. Therefore, despite the push–pull effect, its solvatofluorochromic behavior is less evident compared to TwistTz-M or TwistTz-A. The positive charge on TwistTz-P serves to position it well within biomembranes. Since cellular membranes have a hydrophobic center where VSDs localize, critical for voltage sensitivity, we reasoned that TwistTz-P would retain voltage sensitivity (Table 1).

**Table 1:** Optical properties of the asym-TTz dye (TwistTz-P) in various solvents

Compound	Solvent	Abs. (nm)	Abs. (eV)	Em. (nm)	Em. (eV)	Stokes shift (eV)	Quantum yield (*Φ*_F_)	Fluorescence lifetime (ns)	Radiative rate (s^−1^)	Non-radiative rates (s^−1^)
TwistTz-P	Toluene	445	2.79	507	2.45	0.34	0.08	2.1	0.4 × 10^8^	4.5 × 10^8^
	DCM	436	2.85	557	2.23	0.62	0.32	3.0	1.1 × 10^8^	2.3 × 10^8^
	MeCN	429	2.90	566	2.20	0.70	0.11	6.0	0.2 × 10^8^	1.5 × 10^8^

To quantify the intramolecular charge transfer (ICT) in these new asym-TTz dyes, we used the Lippert–Mataga equation (eqn (1)):1
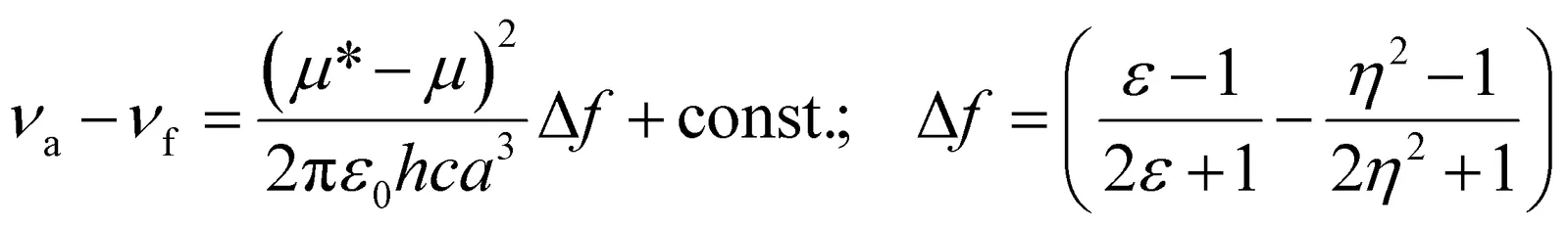
where *ν*_a_ and *ν*_f_ = wavenumbers of the absorption and emission peaks in cm^−1^, respectively; µ* and *µ* = excited state and ground state dipoles, respectively; *ε*_0_ = vacuum permittivity; *h* = Planck's constant; *c* = speed of light; *a* = Onsager cavity radius; Δ*f* = orientation polarizability; *ε* = relative permittivity; and *η* = refractive index.

The Lippert–Mataga plots (SI Section 3.5) showed correlation values > 0.9, indicating negligible solute–solvent interactions, or solute polarizability. We obtained some of the highest ever observed change in dipole moments in these new push–pull asym-TTz dyes.^16,25,34^ Therefore, we expect these dyes to show high environmental sensitivity, suggesting that they might be well suited to sense the cellular membrane potential.^16^ We quantified the change in dipole moments separately for both the SWB and the LWB of TwistTz-P, which were detectable in their emission spectra (SI Fig. S13). The change in dipole moment (23.7 D) of the LWB exceeded that of the SWB (20.3 D), and a closer Lippert–Mataga fit (Fig. 2D and S29, SI) was obtained for the LWB. The SWB is likely more influenced by the polarizability of the solute and the solute–solvent interactions than the LWB, which is the opposite of the trend observed for other asym-TTz derivatives (Table 2).^32^

**Table 2:** Ground and excited state dipole moments

Compound	Onsager radius *a*^a^ (Å)	Ground state dipole moment (*µ*)^a^ (D)	Excited state dipole moment (µ*)^b^ (D)	Change in dipole (Δ*µ*)^b^ (D)
TwistTz-M	8.28	6.82	31.9	25.1
TwistTz-A	8.28	7.52	30.9	23.3
TwistTz-P (LWB)	8.28	6.45	30.2	23.7
TwistTz-P (SWB)	8.28	6.45	26.8	20.3
FlatTz-M	8.20	8.22	34.2	26.0
FlatTz-P	8.21	7.91	36.5	28.6

a Calculated using Spartan 2016 software.

b Semi-empirically calculated using the Lippert–Mataga equation (eqn (1)).

DFT calculations (SI Section 4) show the HOMO (highest occupied molecular orbital) residing over the dibutylaminophenyl (Bu_2_N) donor group whereas the LUMO (lowest unoccupied molecular orbital) resides on the carboalkoxyphenyl acceptor (Fig. 2B), thus generating a push–pull nature. The donor–acceptor nature of these TTz dyes and spatially separated HOMO/LUMO levels shows good electronic overlap across the planar TTz core.

**Fig. 3:**
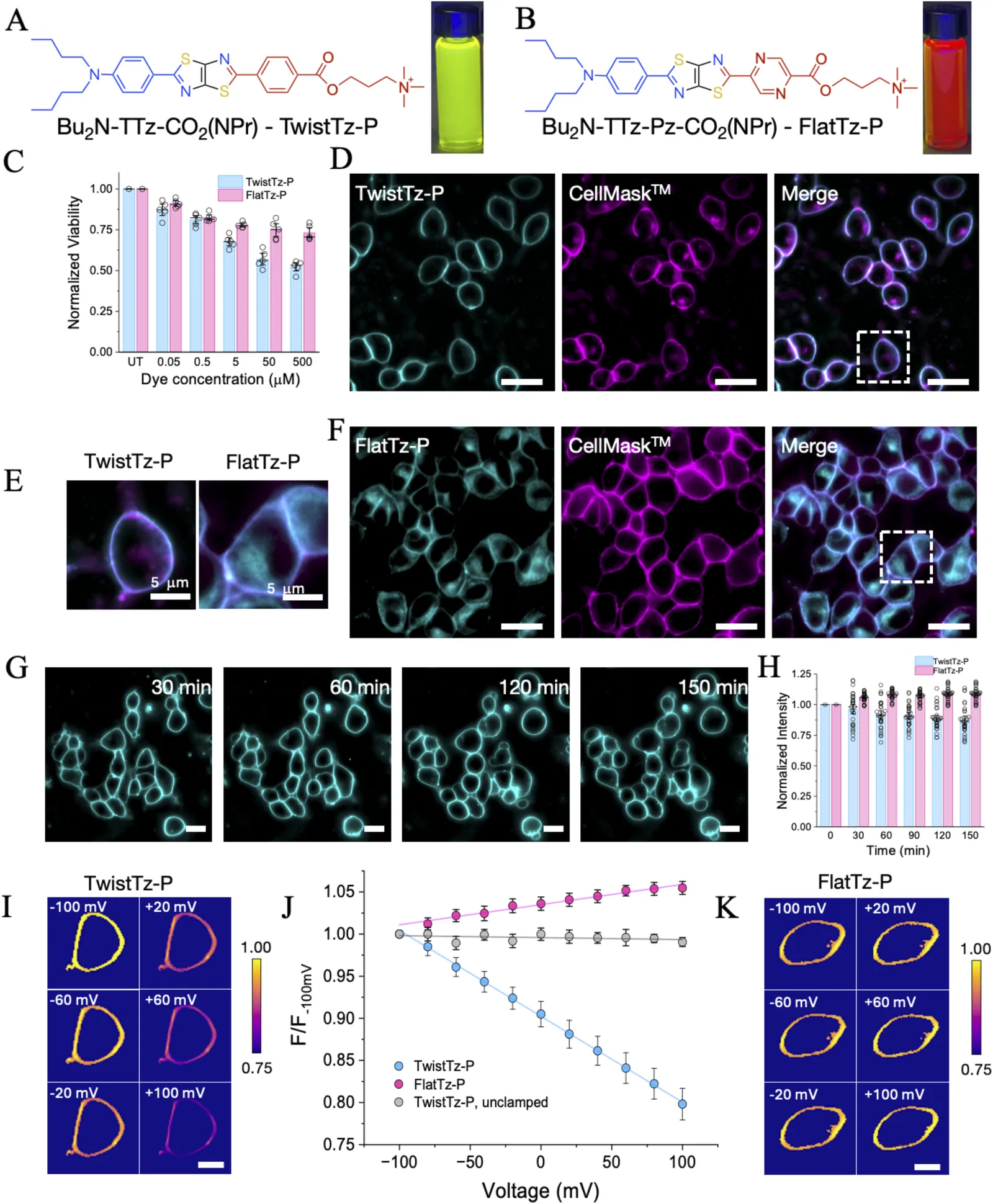
Chemical structures and fluorescence emission (405 nm excitation) of (A) TwistTz-P and (B) FlatTz-P. (C) Cytotoxicity of TwistTz-P and FlatTz-P given by an MTT assay. (D) Fluorescence images of HEK293T cells co-labeled with CellMask™ and TwistTz-P (10 µm scale bar). (E) Magnified regions from (D) and (F) of single co-labeled cells (5 µm scale bar). (F) Fluorescence images of HEK293T cells co-labeled with CellMask™ and FlatTz-P (10 µm scale bar). (G) Fluorescence images of TwistTz-P labeled HEK293T cells at the indicated times showing stability of cell membrane localization over 150 min (10 µm scale bar). (H) Quantification of changes in intensity of TwistTz-P and FlatTz-P labeled cells from (G). (I) *F*/*F*_−100 mV_ ratio map of a single cell labeled with TwistTz-P imaged at the indicated voltage (5 µm scale bar). (J) Fractional change in fluorescence (*F*/*F*_−100 mV_) *versus* membrane potential applied on resting cells or patch clamped whole cells labeled with either TwistTz-P or FlatTz-P. All error bars correspond to standard errors of the mean. (K) *F*/*F*_+100 mV_ ratio map of a single cell labeled with FlatTz-P imaged at the indicated voltage (5 µm scale bar).

We then evaluated the ability of TwistTz-P to report on membrane potential in live cells (Fig. 3). TwistTz-P showed negligible cell toxicity at ≤500 nM, which is the concentration used for all future studies (Fig. 3C). HEK293T cells were co-labeled with an admixture of the plasma membrane marker CellMask™ and 500 nM TwistTz-P (in 1% DMSO/water), and both dyes were imaged by fluorescence microscopy. TwistTz-P and CellMask™ showed excellent colocalization in the plasma membrane (Fig. 3D), with a Pearson's correlation coefficient (PCC) of 0.95 across 15 cells (SI Fig. S40). Next, we evaluated the robustness of cell membrane labeling by TwistTz-P by imaging labeled cells as a function of time. We also found that TwistTz-P remains at the cell membrane without labeling internal membranes of cells even up to 150 min, outperforming the commercial standard CellMask™, which has a plasma membrane retention time of only up to 90 min (Fig. 3G).^35^ We attribute this exceptionally stable localization to the positive charge on the trialkylammonium moiety of TwistTz-P. We expect that TwistTz-P is embedded in the outer leaflet of the cell membrane where the hydrophilic trimethylammonium charged group is oriented towards the membrane/extracellular milieu interface while the hydrophobic dibutylaminophenyl side is membrane-embedded. This behavior is similar to sulfofluoresceins in PeT dyes, where sulfonic acids facilitate proper orientation and prevent cellular internalization.^4^ To test its responsivity to membrane potential, 500 nM TwistTz-P was used to label HEK293T cells, and then imaged at different membrane potentials by holding the cell membrane at specific values of membrane potential, from −100 mV to +100 mV, using whole-cell patch clamp electrophysiology. The fluorescence intensity of TwistTz-P decreased by ∼10% Δ*F*/*F* per 100 mV as the membrane potential moved in the positive direction (Fig. 3I and J). This decrease is not due to photobleaching, since TwistTz-P in resting HEK293T cells showed negligible fluorescence changes for an equivalent number of exposures (Fig. 3J). The high photostability of TwistTz-P and good voltage sensitivity compared to other reported VSDs, *e.g.*, RH-421 (10% Δ*F*/*F* per 100 mV),^36^ di-4-ANNEPPS (15% Δ*F*/*F* per 100 mV),^36^ VF2.1(OMe).Me (13% Δ*F*/*F* per 100 mV),^4^ and other asym-TTz dyes (10% Δ*F*/*F* per 100 mV),^37^ as well as its low toxicity, stable plasma membrane localization, and ease of synthesis, render it attractive for future cellular applications.

**Fig. 4:**
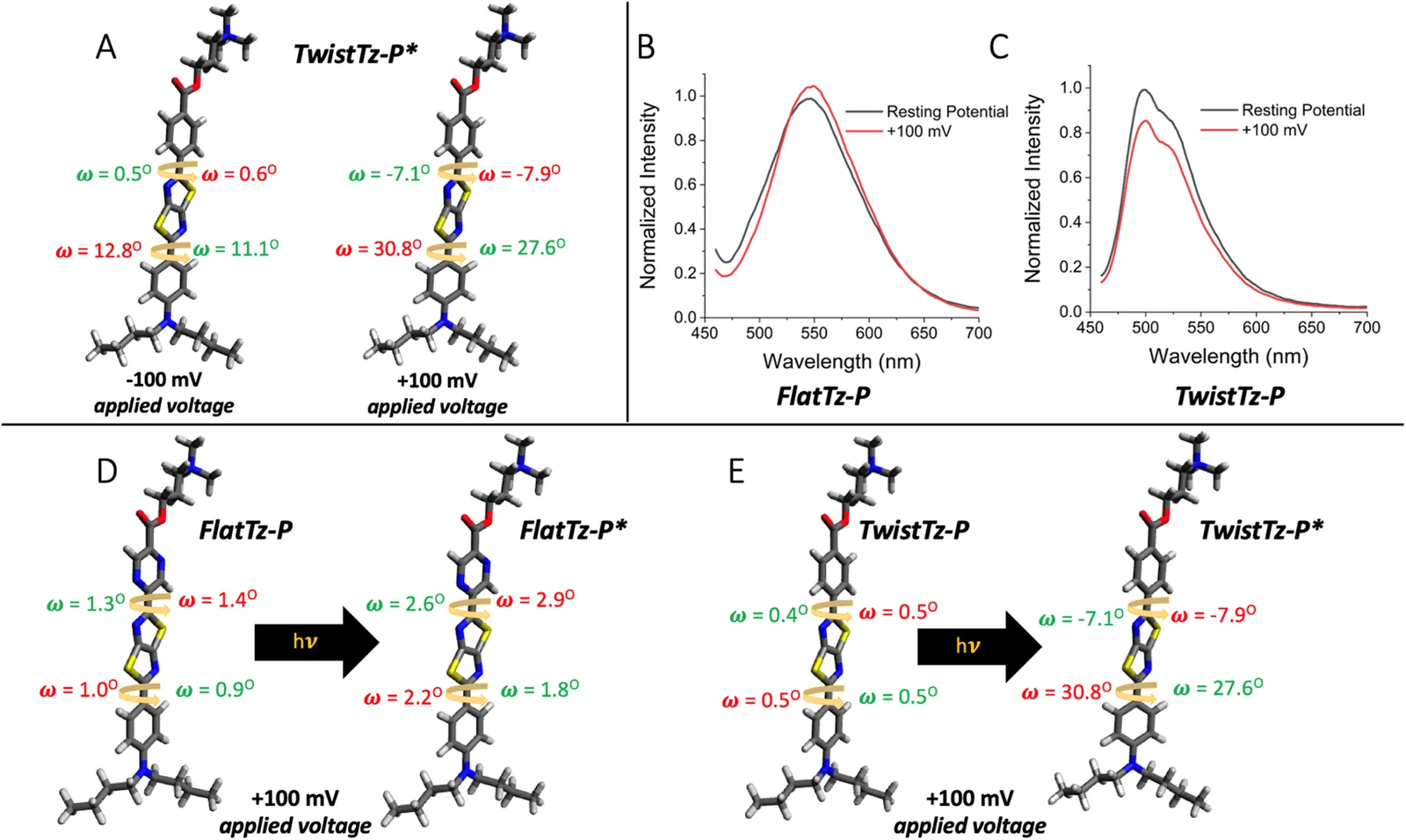
(A) Computed lowest energy excited-state conformation of TwistTz-P at applied potentials of −100 mV (corresponding to hyperpolarization) and +100 mV (corresponding to depolarization), (B) normalized emission spectra of FlatTz-P labeled HEK293T cells excited at 445 nm at resting potential and at +100 mV, (C) normalized emission spectra of TwistTz-P labeled HEK293T cells excited at 445 nm at resting potential and at +100 mV, (D) computed conformation of FlatTz-P in the ground state and in the excited state under an applied potential of +100 mV, (E) computed conformation of TwistTz-P in the ground state and in the excited state under an applied potential of +100 mV; *ω* is the twist angle about the S–C–C–C (red) or N–C–C–C (green) bonds.

To understand the origin of voltage sensitivity in TwistTz-P, we recorded its fluorescence emission spectra in labeled cells (Fig. 4) at resting potential or at +100 mV (SI Fig. S38). Interestingly, TwistTz-P fluorescence in cell membranes shows emission maxima at 487 nm and 525 nm, clearly reflecting the vibronic structure of the dye (Fig. 4 and S38, SI). These vibronic structures are more well-resolved in solutions of asym-TTz dyes in non-polar solvents like *n*-hexane that mimic the hydrophobic lipid interior (Fig. 2C), and their emission spectrum changes depending on the membrane potential. At +100 mV, both 487 and 525 nm emissions decrease, with no spectral shifts in absorbance or emission, characteristics that are generally observed for electrochromic dyes.

To explore how membrane potential affects TTz dye fluorescence, we performed time-dependent DFT (TD-DFT) calculations (SI Sections 7.1–7.3). To mimic the electric field that the TTz dyes would experience within the cell membrane, we simulated applying an electric field along the long axis of the molecule. While there were no appreciable changes in its molecular structure in different solvents (SI Section 7.2), we did observe a significant change in the dihedral angles between the TTz core and the phenyl groups on the donor and the acceptor sides in the excited states (Fig. 4A). Additionally, calculations show that the twist decreased the fluorescence intensity at higher values of membrane potential (Tables S6 and S7), and the TTz core was more planar at lower values of membrane potential (Table S3). Fig. 4D and E show the difference in angles between the TTz core and the adjacent aromatic rings on the donor and acceptor sides of FlatTz-P and TwistTz-P molecules, respectively, in ground and excited states at +100 mV. Note that the twist is greater on the donor phenyl ring than on the acceptor phenyl ring, increasing from 0.5° in the ground state to 30.8° in the excited state on the donor side next to the donor sulfur in TwistTz-P. In contrast, the twist in the same molecule changes from 0.5° in the ground state to −7.9° in the excited state on the acceptor side next to the acceptor sulfur of the TTz core. All additional ground and excited state angles under different applied voltages are provided in the SI (Section 7). In the twisted excited state, the light emission is less efficient since the p-orbitals are poorly aligned when twisted, thereby reducing π-conjugation and/or increasing the probability of non-radiative processes. When the applied potential is more positive, it is computationally observed that the relative oscillator strength decreases, making the transition less favorable and thus affecting the fluorescence intensity. This is consistent with our observed experimental data (Fig. 3I and J), which show that emission decreases as membrane potential becomes more positive and the twisted state is more favored.

Twisted intramolecular charge transfer (TICT) mechanisms are not uncommon, especially in dialkylaminophenyl donor group-containing fluorophores whereby excited states may rapidly (in the fs to ps timescale) planarize or induce significant twisting between the dialkylamino and conjugated bridge of the fluorophore.^38–40^ As previously discussed, steady-state fluorescence supports the presence of a TICT (as seen with other asym-TTz fluorophores developed in our lab) and is consistent with the classical description of strong electron donors and acceptors within a conjugated, push–pull molecular framework.^41^ Our data suggest an additional molecular twisting around the TTz core that is dependent on the applied voltage. Although several studies have studied field-induced twisting across a single molecular junction in the absence of light excitation,^42–44^ the arrangement described here in which donor–acceptor molecules are aligned within a membrane, photoexcited, and subjected to an applied electric field is unique.

To test our hypothesis, using the synthetic procedure described in Fig. 1, we synthesized an analogous set of TTz compounds with a carboxypyrazinyl group on the electron acceptor side instead of the aforementioned carboxyphenyl group, namely Bu_2_N-TTz-Pz-CO_2_CH_3_ (Flat TTz Methyl, FlatTz-M), Bu_2_N-TTz-Pz-CO_2_H (Flat TTz Acid, FlatTz-A) and Bu_2_N-TTz-Pz-NPr (Flat TTz Propyl, FlatTz-P) (Fig. 1 and 3B). The carboxypyrazinyl compounds have a far more red-shifted emission compared to the carboxyphenyl compounds. The fluorescence emission in chloroform shifted from greenish yellow (545 nm) for TwistTz-P to red (645 nm) for FlatTz-P (Fig. 3B and Table S1). As per the DFT calculations and the Lippert–Mataga plots for these carboxypyrazinyl compounds, FlatTz-M and TwistTz-M exhibited a comparable change in dipole moment of 26.0 D and 25.1 D, respectively. These pyrazinyl derivatives were synthesized based on their computational behavior under different electric fields in the excited state. When FlatTz-P was subjected to different voltages, it showed insignificant twisting (<3°) in the dihedral angles between the TTz core and the donor/acceptor side chains in the excited states (Fig. 4 and Tables S5, S6). This drastic reduction in twisting under applied voltage leads to the prediction that FlatTz-P should be almost voltage insensitive. To test this prediction, we tested the toxicity and voltage sensitivity of FlatTz-P in HEK293T cells. FlatTz-P showed negligible cytotoxicity and stable cell membrane localization for up to 150 min, with an average PCC of 0.79 in 15 cells (Fig. S39 and 3C). Importantly, FlatTz-P showed negligible voltage sensitivity in the cell membranes, with ∼2% change over 100 mV, and a slight increase in fluorescence intensity as voltage increases to +100 mV (Fig. 3J and K). Furthermore, since the changes in dipole moments of the carboxyphenyl and carboxypyrazinyl derivatives are similar, we can pinpoint the voltage sensitivity of TwistTz-P to the geometric changes in its excited state when exposed to differing electric fields. Thus, the voltage sensitivity of TwistTz-P can be attributed to molecular twisting in its excited state, as reflected in the dihedral angles between the TTz core and the electron donor and acceptor groups.

## Conclusions

Asym-TTz dyes (TwistTz-P and FlatTz-P), accessed *via* an improved one-step synthesis, unveil a new mechanism of voltage sensing by a molecular fluorophore. The superior membrane localization and orientation of TwistTz-P lead to membrane potential modulating a dihedral twist on either side of the thiazolothiazole bridge in the excited state. This mechanism controls fluorescence emission intensity without shifting the emission peak. Since the voltage sensing mechanism relies on twisting, these fluorophores do not introduce a capacitive load on the biological membrane. Additionally, because the mechanism involves changes in excited state geometry, response times are much faster than FRET-based protein reporters or the “slow VSDs” and comparable to “fast sensing” RVF dyes.

This method of sensing voltage is truly unique, leading to the establishment of a brand-new class of voltage sensitive dyes, with parameters never described before. Unlike TwistTz-P, its structural variant FlatTz-P showed negligible twisting in the excited state computationally, and voltage sensitivity in FlatTz-P was effectively eliminated when tested in HEK293T cells. These studies provide evidence of the necessity of the electric field-dependent excited-state twist for voltage sensitivity to occur with asym-TTz dyes. Their similar changes in dipole moments imply that voltage sensing behavior cannot be attributed solely to the difference in their dipole moments between the ground and excited states, and that geometric changes in their excited states are the primary factors governing their voltage sensitivity. Our studies reveal that this sensing mechanism in the asym-TTz dyes is unlike that seen in PeT dyes, where a fluorophore is quenched by an electron-rich molecular wire.^10^ It also differs from that of electrochromic dyes, in which the excitation and emission characteristics, *i.e.*, those of the ground state and the excited state, shift as the membrane potential changes *via* the molecular Stark effect.^45^ Lastly, most VSDs exhibit an increase in fluorescence during depolarization, while some electrochromic dyes and certain d-PeT VF dyes show a decrease in fluorescence with higher membrane potentials.^46,47^ The ability to observe cells under hyperpolarization conditions with voltage-sensitive asym-TTz fluorophores provides a complementary addition to the available VSD palette. These observations position the TTz core as a promising scaffold for developing more sensitive reporters for voltage imaging in biological systems.

## Author contributions

Conceptualization: P. C., A. N., Y. K., and M. G. W. Experimentation and data analysis: P. C., R. F., L. M. W., C. A. L. D., J. D. A., D. O., and A. N. Funding acquisition: Y. K. and M. G. W. Project administration and supervision: A. N., Y. K., and M. G. W. Writing – original draft: P. C., R. F., and A. N. Writing – review and editing: P. C., Y. K., and M. G. W.

## Conflicts of interest

The authors declare no competing financial interests.

## Supplementary Material

SC-OLF-D6SC04210D-s001

## Data Availability

The data that support the findings of this study are available in the supplementary information (SI) of this article. Supplementary information: synthetic procedures, instrumentation details, optical energy gaps, absorption and emission spectra, DFT (TD-DFT) calculations, procedures for spectral measurements of dye-labeled cells, microscopy, cellular localization, and all spectrophotometric titration data. See DOI: https://doi.org/10.1039/d6sc04210d.
