# Supplementary material for: Electric field-dependent excited-state twisting in donor–acceptor thiazolothiazole dyes establishes a new class of cell membrane voltage sensors
Source: Chem Sci. 2026 Aug 25;17(42):20270–8. doi: 10.1039/d6sc04210d (PMC13598645; doi:10.1039/d6sc04210d)
Supplement: Supplementary file 1 [file SC-017-D6SC04210D-s001.pdf]

Supplementary Information (SI) for Chemical Science.
This journal is © The Royal Society of Chemistry 2026

Supplementary Information

Electric Field-Dependent Excited-State Twisting in Donor-Acceptor Thiazolothiazole Dyes Establishes a New Class of Cell Membrane Voltage Sensors

Pranamita Chakraborti,[†] *Rui Fu,*[‡] *Lucia M. Wert,*[†] *Charlie A.L. Darby,*[†] *Joy D. Amuzu,*[†]

Daphne Oettinger,[‡] *Aditya Nandy**[§] *Yamuna Krishnan**[‡] *and Michael G. Walter**[†]

[†]Department of Chemistry, University of North Carolina at Charlotte, Charlotte, NC, 28223,

[‡]Department of Chemistry, The University of Chicago, Chicago, IL 60637

[‡]Neuroscience Institute, The University of Chicago, Chicago, IL 60637

[‡]Institute of Biophysical Dynamics, The University of Chicago, Chicago, IL 60637

[§] Department of Chemical and Biomolecular Engineering, University of California, Los Angeles, Los Angeles, CA 90095

*aditya.nandy@ucla.edu

*yamuna@uchicago.edu

*Michael.Walter@charlotte.edu

Table of Contents

Section 1: Materials and Methods 3
Section 1.1: Materials and Instrumentation 3
Section 1.1.1: Cell Labeled Spectral Measurements 3
Section 1.1.2: Electrophysiology 4
Section 1.1.3: Microscopy 5
Section 1.1.4: Cellular Localization 6
Section 1.1.5: Cytotoxicity 6
Section 1.2: Synthesis 7
Section 2: Spectra Characterization 11
Section 2.1: ^{1}H-NMR 11
Section 2.2: MALDI-TOF MS 16
Section 3: Solvent Sensitivity 18
Section 3.1: Emission 18
Section 3.2: Fluorescence Lifetimes 22
Section 3.3: Quantum Yield and Molar Absorptivity 25
Section 3.4: Optical Properties 27
Section 3.5: Lippert-Mataga Plots 28
Section 3.6: Polarizability 30
Section 4: Computation 31
Section 5: Emission of Planar and Twisted States 36
Section 6: Emission Studies in Cells 37
Section 7: Molecular Angles with Applied Voltage 38
Section 7.1: Angles for TwistTz-P and FlatTz-P in excited and ground states 38
Section 7.2: Angles for TwistTz-P in different solvents 40
Section 7.3: Molecular Angles with Applied Voltage 41

Section 1: Materials and Methods
Section 1.1: Materials and Instrumentation

4-(dibutylamino)benzaldehyde, dithiooxamide, methyl-4-formylbenzoate, (3-bromopropyl)trimethylammonium bromide, and all solvents used for spectroscopic measurements were purchased from Sigma-Aldrich and used without further purification. ^{1}H-NMR measurements were obtained with either a JEOL 300 or 500 MHz or Bruker 500 MHz NMR. High resolution mass spectra were obtained using a Voyager Matrix Assisted Laser Desorption/Ionization Time-of-Flight (MALDI-TOF) mass spectrometer, using 1,8,9-Trihydroxyanthracene as a matrix. Solution-state UV-Vis spectra were collected on a Cary 300 UV-Vis spectrophotometer. Time-resolved measurements were taken on a Horiba Scientific Fluoromax+ TCSPC spectrofluorometer equipped with a 405 nm diode laser for time-resolved PL decay measurements. All decays were calculated with a $\mathcal{X}^2$ ~1.2. Igor Pro 6.3 software was used to fit PL(*t*) decay data to single/multiple exponential decays. Quantum yields were calculated using perylene orange reference (quantum yield [Φ_F] in chloroform = 0.99 ± 0.05).

Section 1.1.1: Cell culture. HEK293T cells were cultured in Dulbecco's Modified Eagle's Medium (Invitrogen Corporation, USA) containing 10% heat inactivated Fetal Bovine Serum (FBS) (Invitrogen Corporation, USA), 100 U/mL penicillin, and 100 µg/mL streptomycin. Cells were maintained at 37 ºC under 5% CO_2.

Section 1.1.2: Cell labeled spectral measurements. Excitation and emission spectra were obtained by labeling HEK293T cells (0.5×10^5) with 500 nM TwistTz-P and FlatTz-P in phosphate-buffered saline (PBS). After a 10-min incubation at 37 °C, the cells were washed with PBS and divided into four treatment groups: Group 1: cells resuspended in PBS. Group 2: cells resuspended in buffer containing 200 mM NaCl and 5 mM TEA-Cl. Group 3: cells fixed with 4%

paraformaldehyde (PFA) for 10 min, then resuspended in PBS. Group 4: cells permeabilized with 50 µM digitonin in PBS, then resuspended in PBS. Labeled cells were transferred to a quartz cuvette, and fluorescence spectra were recorded using a Fluoromax-4 spectrofluorometer (Horiba Scientific, Edison, NJ, USA). Excitation spectra were collected at an emission wavelength of 520 nm, and emission spectra were acquired with excitation at 445 nm.

Section 1.1.3: Electrophysiology. Whole-cell voltage-clamp measurements were conducted on HEK293T cells labeled with 500 nM TwistTz-P and FlatTz-P. The experiments utilized an Axopatch 200B amplifier (Molecular Devices) for voltage clamping, with digitization performed using an NI-6251 DAQ (National Instruments) and NI-6501 USB (National Instruments), and control facilitated by WinWCP v5.8.1 software (Strathclyde Electrophysiology Software). Imaging was carried out using an IX83 WF microscope at 60X magnification. Membrane seals were established using micropipettes filled with intracellular buffer, drawn from borosilicate glass capillaries pulled via Sutter P-97 Micropipette puller and polished with a Coater/Polisher Microforge (ALA Scientific Instruments Inc.). Patch pipettes with resistances between 1-4 M Ohm were employed in the voltage-clamp experiments and positioned using an MP325 motorized manipulator (Sutter). OLYMPUS cellSens Dimension 4.4 was synchronized with an NI-6501 USB to enable voltage-triggered image acquisition. The extracellular solution composition was 145 mM NaCl, 10 mM HEPES, 3 mM KCl, 2 mM $CaCl_2$, 1 mM $MgCl_2$, 20 mM glucose, pH 7.4 (310 mOsm), while the intracellular solution composition was 115 mM potassium gluconate, 10 mM EGTA tetrapotassium salt, 10 mM HEPES, pH 7.2, 5 mM NaCl, 10 mM KCl, 2 mM ATP disodium salt (290 mOsm). After achieving whole-cell mode, the membrane was held at 0 mV, and step pulses were provided ranging from -100 mV to +100 mV with an increment of 20 mV at each step for 5 seconds. Simultaneously, at each voltage step, images were snapped for analysis.

PFA Fixation

Labeled cells were resuspended in 4% paraformaldehyde (PFA) in PBS and incubated on an orbital shaker for 10 min at room temperature. Following fixation, the cells were centrifuged at 500 × g for 5 min, the supernatant was discarded, and the pellet was resuspended in PBS for fluorescence spectral measurements. Fluorescence intensity was normalized to cell count.

Digitonin Treatment

Labeled cells were resuspended in PBS containing 50 µM digitonin and incubated on an orbital shaker for 10 min at room temperature. Cells were then centrifuged at 500 × g for 5 min, the supernatant was removed, and the pellet was resuspended in PBS for subsequent fluorescence spectral recording. Fluorescence intensity was normalized to cell count.

Section 1.1.4: Microscopy. Imaging was carried out on an IX83 inverted microscope (Olympus Corporation of the Americas, Center Valley, PA, USA). This setup included a 100X, 1.4 NA, oil immersion objective (UPlanSApo, Olympus), a 60X, 1.42 NA, oil immersion objective (PlanApo, Olympus) and an CMOS camera (ORCA-Flash4.0, HAMAMATSU). Control over the Filter wheel, shutter, and CMOS camera were facilitated by OLYMPUS cellSens Dimension 4.4.

For photostability studies, HEK293T cells were labeled with 500 nM TwistTz-P and FlatTz-P for 10 mins followed by a wash with PBS. Subsequently, the cells were illuminated with a light source emitting ~5 W/cm^2 power. Images were captured at 30 min intervals till 150 min using specific filter configurations: 430/24 bandpass excitation filter, 535/50 bandpass emission filter and Di02-R532 dichroic. For CellMask™ red (Invitrogen Corporation, USA), images were obtained using the 640/30 bandpass excitation filter, 705/72 bandpass emission filter and 89007 dichroic.

Section 1.1.5: Cellular localization: To explore the prolonged membrane localization of TwistTz-P and FlatTz-P, time-lapse imaging was employed. HEK293T cells were initially treated with 500nM TwistTz-P, FlatTz-P, and a plasma membrane (CellMask^{TM}) for 10 min in 1X HBSS (Hank's balanced salt solution, Thermofisher). Subsequent to this labeling, any excess unbound dyes were eliminated via a wash with 1X PBS. The membrane-associated TwistTz-P, FlatTz-P, and CellMask^{TM} Red were sequentially imaged every 30 mins till 150 min.

Section 1.1.6: Cytotoxicity: The cellular toxicity of the voltage-sensitive dye TwistTz-P and FlatTz-P was evaluated using a mitochondrial respiration assay involving dimethylthiazol diphenyl tetrazolium (MTT) staining (CyQuant MTT Cell Proliferation Assay Kit, Invitrogen). Initially, a 12 mM MTT stock solution was prepared by dissolving one 5mg vial of MTT in 1ml of sterile PBS, which was then vortexed or sonicated until fully dissolved. HEK293T cells were cultured and treated with various concentrations of the dye, ranging from 0.05 to 500 μM, for 10 mins at 37 ºC, followed by a PBS wash. Subsequently, 10 μl of the 12mM MTT stock solution was added to 100 μl of freshly replaced culture media, followed by an incubation period at 37 ºC for 2-5 h. After removing most of the medium from the wells, leaving only 25 μl, 50 μl of DMSO was added and thoroughly mixed by pipetting. Absorbance at 540nm was measured after a further incubation with DMSO at 37 ºC for 10 min. Based on these absorbance values, the normalized cell viability was calculated and plotted against the concentration of the dye used in the experiment.

Section 1.2: Synthesis of asym-TTz molecules. TwistTz-M was synthesized using a one-step mixed pot reaction and was hydrolyzed to form an acid, TwistTz-A, and subsequently the TwistTz-P derivative, as is discussed in the manuscript. A similar strategy was used to access FlatTz-M and FlatTz-P.

Section 1.2.1: 2-(N,N-dibutyl-4-aminophenyl)-5-(4-carbomethoxyphenyl) thiazolo[5,4-d]thiazole (Bu_2N-TTz-CO_2CH_3) – ***TwistTz-M***

Dithiooxamide (0.175 g, 1.45 mmol), 4-(dibutylamino)benzaldehyde (0.373 g, 1.6 mmol), and 4-methyl-4-formylbenzaldehyde (0.263 g, 1.6 mmol) were refluxed in 10 mL of 1-propanol (in air at 97°C) for 48 h. The reaction was cooled to room temperature and left to sit in a fridge (~2 °C) for 24 h. The product was collected using gravity filtration and rinsed with 30-40 mL ice-cold n-propanol to isolate an orange solid (0.151 g, 22% yield). The isolated product was found to be sufficiently pure for subsequent synthetic steps. Only a single fluorescent yellow band corresponding to the target product was observed during both thin-layer chromatography (TLC) and silica gel column chromatography. Molecular characterization data (^{1}H-NMR, MALDI-TOF MS, and photophysical analyses) was collected on a sample purified using silica gel column chromatography using hexane: ethyl acetate 6:1 (7.2 mg was purified to yield 4.1 mg of product, 57% recovery). Nearly identical results (^{1}H-NMR, MALDI-TOF MS, and silica TLC) were obtained from the isolated and the purified product. **^{1}H NMR** (300 MHz, Chloroform-d, δ): 8.12 (d, J = 8.52 Hz, 2H), 8.03 (d, J = 8.52 Hz, 2H), 7.82 (d, J = 8.79 Hz, 2H), 6.66 (d, J = 9.06 Hz, 2H), 3.95 (s, 3H), 3.34 (t, J = 7.70 Hz, 4H), 1.61 (m, 4H), 1.33 (m, 4H), 0.97 (t, J = 7.29 Hz, 6H). **UV-Vis λ_{max} ($CHCl_3$, ε = $M^{-1}cm^{-1}$):** 430 nm (ε = 45,000). **MALDI-TOF MS:** m/z calculated for $C_{26}H_{30}N_3O_2S_2^+$, 480.1774; found, 480.7234. $\boldsymbol{\Phi_F}$ = 0.98 (hexane).

Section 1.2.2: 2-(N,N-dibutyl-4-aminophenyl)-5-(4-caroboxyphenyl) thiazolo[5,4-d]thiazole (Bu_2N-TTz-CO_2H) – ***TwistTz-A***

2-(N,N-dibutyl-4-aminophenyl)-5-(4-carbomethoxyphenyl) thiazolo[5,4-d]thiazole (0.025 g, 0.05 mmol) was heated with 2 M potassium hydroxide (5 mL), and 95% ethanol (5 mL) for 48 h at 70°C in an aerated environment. The reaction was cooled to 25°C and pH adjusted to ~2 using 1:1 conc. HCl: H_2O. Using dichloromethane: methanol (4:1) the product

was purified using silica gel column chromatography to obtain 0.002 g of product, 86% yield. **^{1}H NMR** (500 MHz, DMSO-d6, δ): 8.00 (d, *J* = 8.25 Hz, 2H), 7.92 (d, *J* = 8.25 Hz, 2H), 7.73 (d, *J* = 9.2 Hz, 2H), 6.68 (d, *J* = 9.2 Hz, 2H), 3.29 (t, 4H), 1.46 (m, 2H), 1.28 (m, 2H), 0.86 (t, *J* = 7.55 Hz, 6H). **UV-Vis λ_{max} ($CHCl_3$, ε = $M^{-1}cm^{-1}$):** 429 nm (ε = 15,000). **MALDI-TOF MS:** m/z calcd for $C_{25}H_{28}N_3O_2S_2^+$, 466.1618; found 466.8454. Φ_F = 0.49 ($CHCl_3$).

*Section 1.2.3: 2-(N,N-dibutyl-4-aminophenyl)-5-(4-carbo[3-(trimethylammonio)propoxy]phenyl) thiazolo[5,4-d]thiazole bromide [Bu_2N-TTz-CO_2(NPr)] – **TwistTz-P***

2-(N,N-dibutyl-4-aminophenyl)-5-(4-carboxyphenyl) thiazolo[5,4-d]thiazole **(*TwistTz-A*)** (0.027 g, 0.06 mmol), (3-bromopropyl)trimethylammonium bromide (0.094 g, 0.36 mmol) and potassium carbonate (0.066 g, 0.48 mmol) were mixed in 30 mL acetone for 24 h at 52 °C. The reaction mixture was cooled to room temperature and allowed to sit overnight. Solvent was removed and remaining solids were rinsed/centrifuged with hexane and collected via gravity filtration (0.033 g, 97% yield). Further rinsing/centrifugation with hexane (with minimal loss of yield) helped to remove a minor impurity only detectable as a weakly fluorescent spot on TLC (using a high intensity UV light). **^{1}H NMR** (500 MHz, d-DCM, δ): 8.09 (d, *J* = 8.7 Hz, 2H), 8.00 (d, *J* = 8.7 Hz, 2H), 7.74 (d, *J* = 9.15 Hz, 2H), 6.61 (d, *J* = 9.15 Hz, 2H), 4.44 (t, *J* = 5.95 Hz, 2H), 3.81 (t, *J* = 8.48 Hz, 2H), 3.37 (s, 9H), 3.28 (t, *J* = 13.73 Hz, 4H), 2.26 (m, 2H), 1.54 (m, 4H), 1.32 (m, 4H), 0.90 (t, *J* = 7.33 Hz, 6H). **UV-Vis λ_{max} ($CHCl_3$, ε = $M^{-1}cm^{-1}$):** 437 nm. **MALDI-TOF MS:** m/z calcd for $C_{31}H_{41}N_4O_2S_2^+$, 565.2666; found 565.8606. Φ_F = 0.40 ($CHCl_3$).

*Section 1.2.4: 2-(N,N-dibutyl-4-aminophenyl)-5-(4-carbomethoxypyrazinyl) thiazolo[5,4-d]thiazole (Bu_2N-TTz-Pz-CO_2CH_3) – **FlatTz-M***

Dithiooxamide (0.178 g, 1.48 mmol), 4-(dibutylamino)benzaldehyde (0.371 g, 1.59 mmol), and methyl-5-formyl-2-pyrazinecarboxylate (0.254 g, 1.53 mmol) were refluxed in 10

mL of 1-propanol (in air at 97°C) for 48 h. The reaction was cooled to room temperature. The product was collected using gravity filtration and rinsed with 30-40 mL ice-cold 1-propanol to isolate a red solid (0.1102 g, 16% yield). The isolated product was found to be sufficiently pure for subsequent synthetic steps. Only a single fluorescent band corresponding to the target product was observed during both thin-layer chromatography (TLC) and silica gel column chromatography. Molecular characterization data (^{1}H-NMR, MALDI-TOF MS, and photophysical analyses) was collected on a sample purified using silica gel column chromatography using chloroform (18.4 mg was purified to yield 7.2 mg of product, 39% recovery). Nearly identical results (^{1}H-NMR, MALDI-TOF MS, and silica TLC) were obtained from the isolated and the purified product. **^{1}H NMR** (500 MHz, Chloroform-d, δ): 9.44 (s, 1H), 9.18 (s, 1H), 7.80 (d, *J* = 5 Hz, 2H), 6.63 (d, 2H), 4.00 (s, 3H), 3.29 (t, *J* = 7.5 Hz, 4H), 1.55 (m, 4H), 1.31 (m, 4H), 0.92 (t, *J* = 7.5 Hz, 6H) . **UV-Vis λ_{max} ($CHCl_3$, ε = $M^{-1}cm^{-1}$):** 486 nm (ε = 69,000). **MALDI-TOF MS:** m/z calcd for $C_{24}H_{28}N_5O_2S_2^+$, 482.1679, found 482.5244. Φ_F = 0.51 ($CHCl_3$).

Section 1.2.5: 2-(N,N-dibutyl-4-aminophenyl)-5-(4-caroboxyphenyl) thiazolo[5,4-d]thiazole (Bu_2N-TTz-Pz-CO_2H) – ***FlatTz-A***

2-(N,N-dibutyl-4-aminophenyl)-5-(4-carbomethoxypyrazinyl) thiazolo[5,4-d]thiazole (0.05 mmol, 0.025 g) was reacted with 2 M potassium hydroxide (5 mL), and 95% ethanol (5 mL) for 48 h at 70°C in an aerated environment. The reaction was cooled to room temperature and pH was then adjusted to ~2 using 1:1 conc. HCl: H_2O solution to obtain 0.012 g (51% yield) of a red solid product. Only one fluorescent band indicating the target product was observed. The product was used for the subsequent reaction without further purification.

*Section 1.2.6: 2-(N,N-dibutyl-4-aminophenyl)-5-(4-carbo[3-(trimethylammonio)propoxy]pyrazinyl) thiazolo[5,4-d]thiazole bromide [Bu_2N-TTz-Pz-CO_2(NPr)] – **FlatTz-P***

2-(N,N-dibutyl-4-aminophenyl)-5-(4-carboxypyrazinyl) thiazolo[5,4-d]thiazole (0.0155 g, 0.033 mmol), (3-Bromopropyl)trimethylammonium bromide (0.0284 g, 0.11 mmol) and potassium carbonate (0.0347 g, 0.251mmol) were mixed in 10 mL acetone for 72 h at 52 °C. The reaction mixture was cooled to room temperature and left overnight. Solid product was collected using gravity filtration and rinsed/centrifuged with hexane (0.0098 g, 52% yield). Further rinsing/centrifugation with hexane (with minimal loss of product yield) helped to remove a minor impurity only detectable as a weakly fluorescent spot on TLC (using a high intensity UV light). **^{1}H NMR** (500 MHz, d-DCM, δ): 9.37 (s, 1H), 9.18 (s, 1H), 7.76 (d, J = 10 Hz, 2H), 6.62 (d, 2H), 4.53 (t, J = 5 Hz, 2H), 3.83 (t, 2H), 3.40 (s, 9H), 3.28 (t, J = 10 Hz, 4H), 2.3 (m, 2H), 1.54 (m, 4H), 1.32 (m, 4H), 0.90 (t, J = 7.5 Hz, 6H). **UV-Vis λ_{max} (DMSO, ε = $M^{-1}cm^{-1}$):** 483 nm, (ε = 14,000). **MALDI-TOF MS:** m/z calcd for $C_{29}H_{39}N_6O_2S_2^+$, 567.2571; found 567.2131. $\boldsymbol{\Phi}_{\mathbf{F}}$ = 0.23 ($CHCl_3$).

Section 2: Spectra Characterization

Section 2.1: ^{1}H-NMR

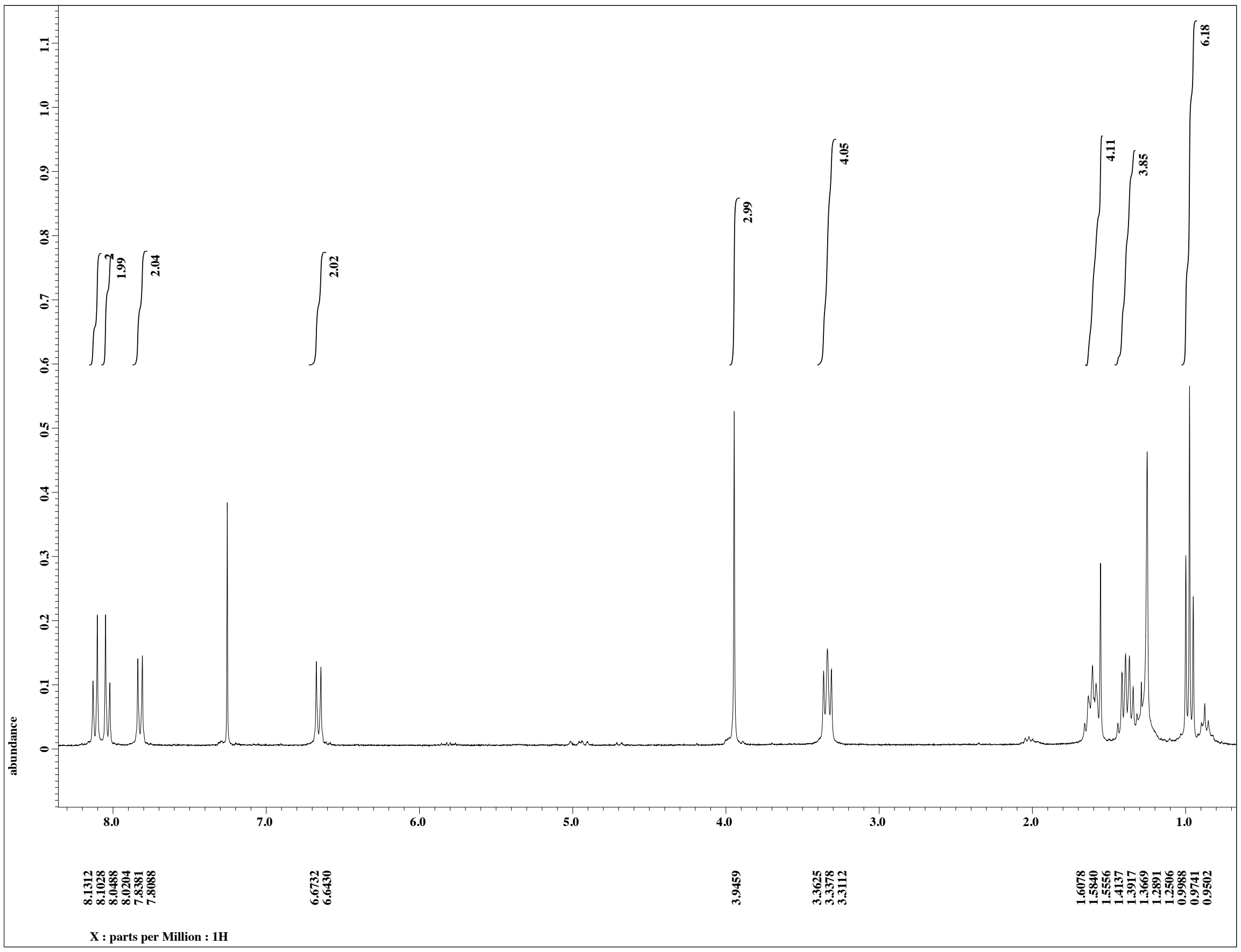

Figure S1: ^{1}H NMR of TwistTz-M

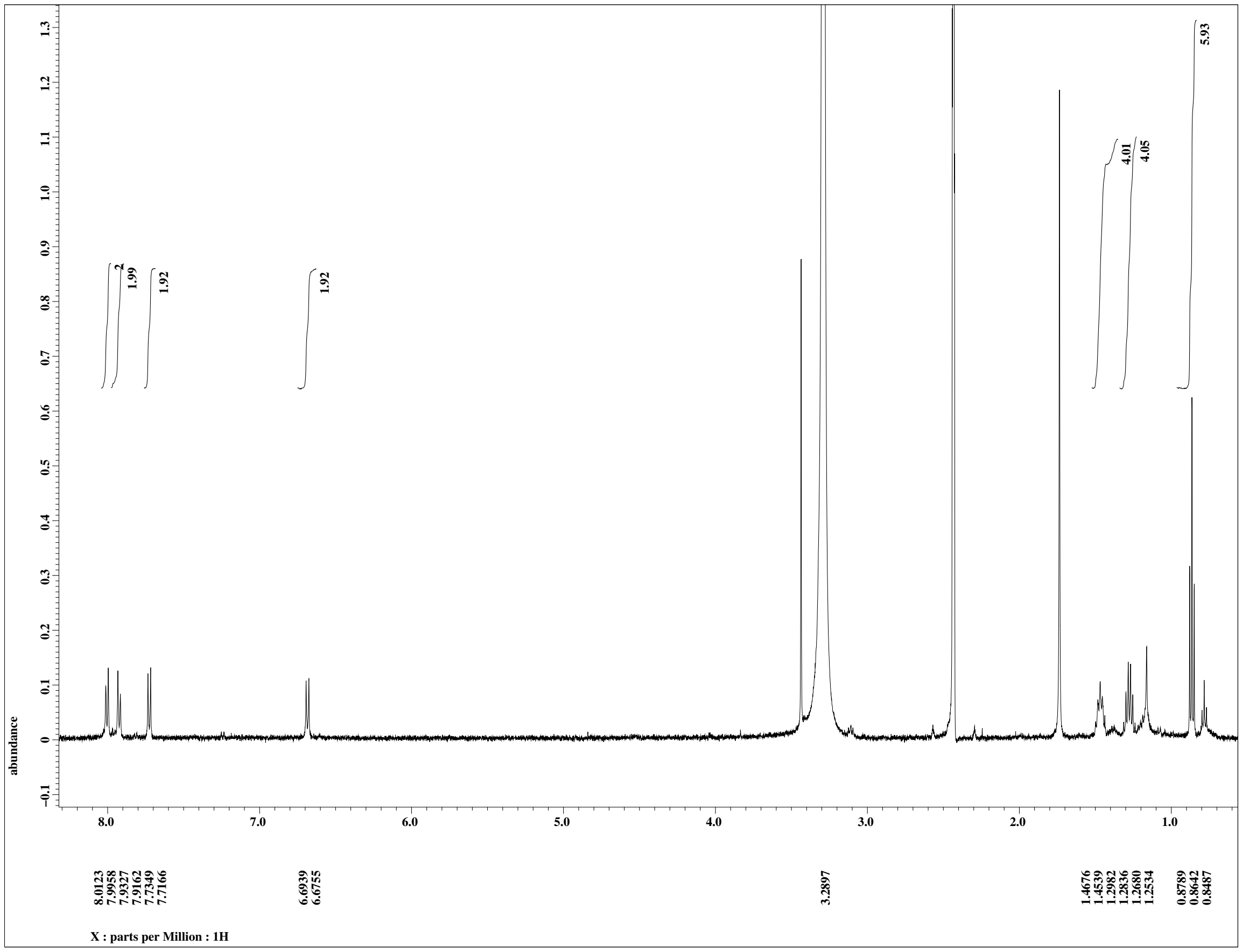

Figure S2: ^{1}H NMR of TwistTz-A

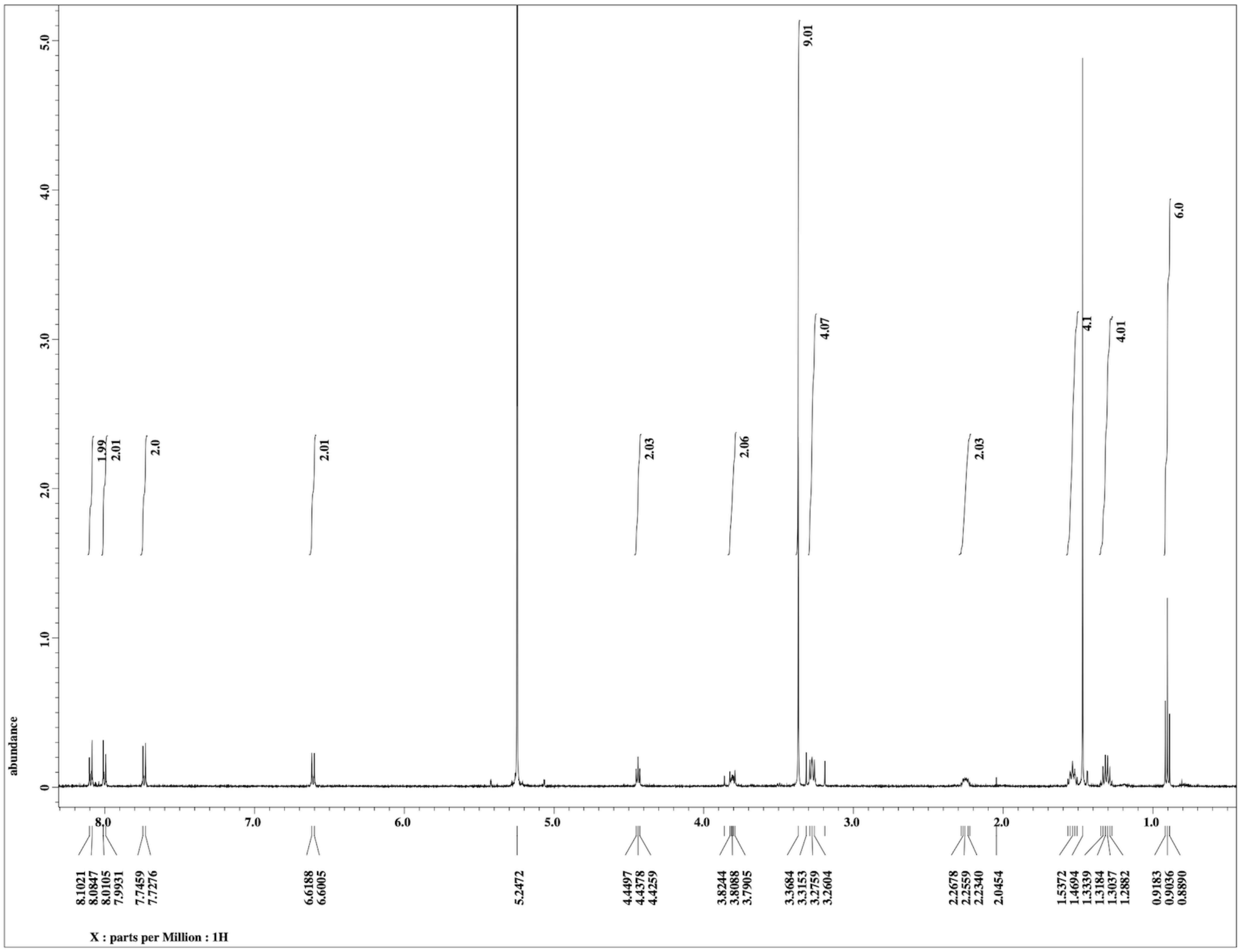

Figure S3: ^{1}H NMR of TwistTz-P

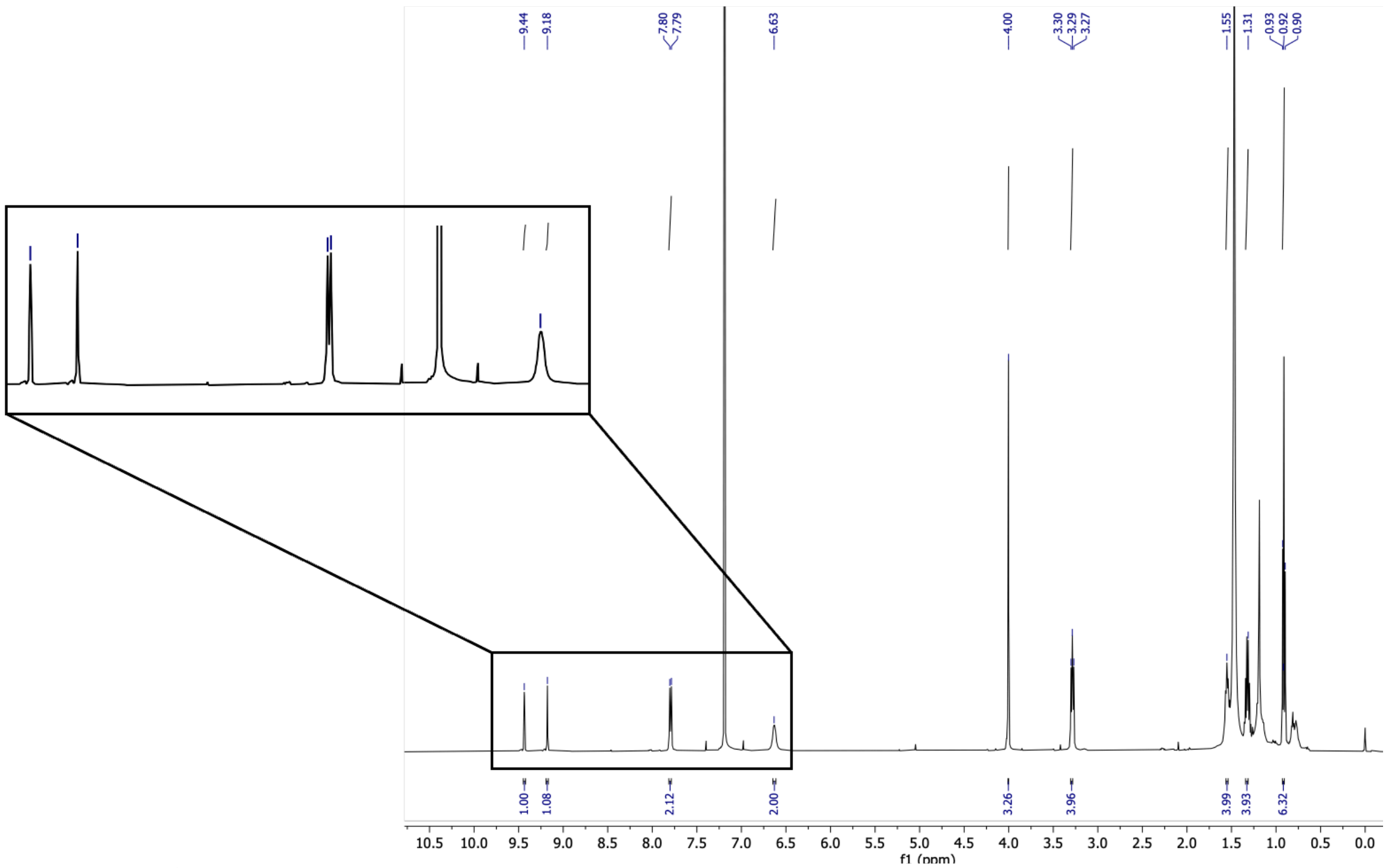

Figure S4: ^{1}H NMR of FlatTz-M

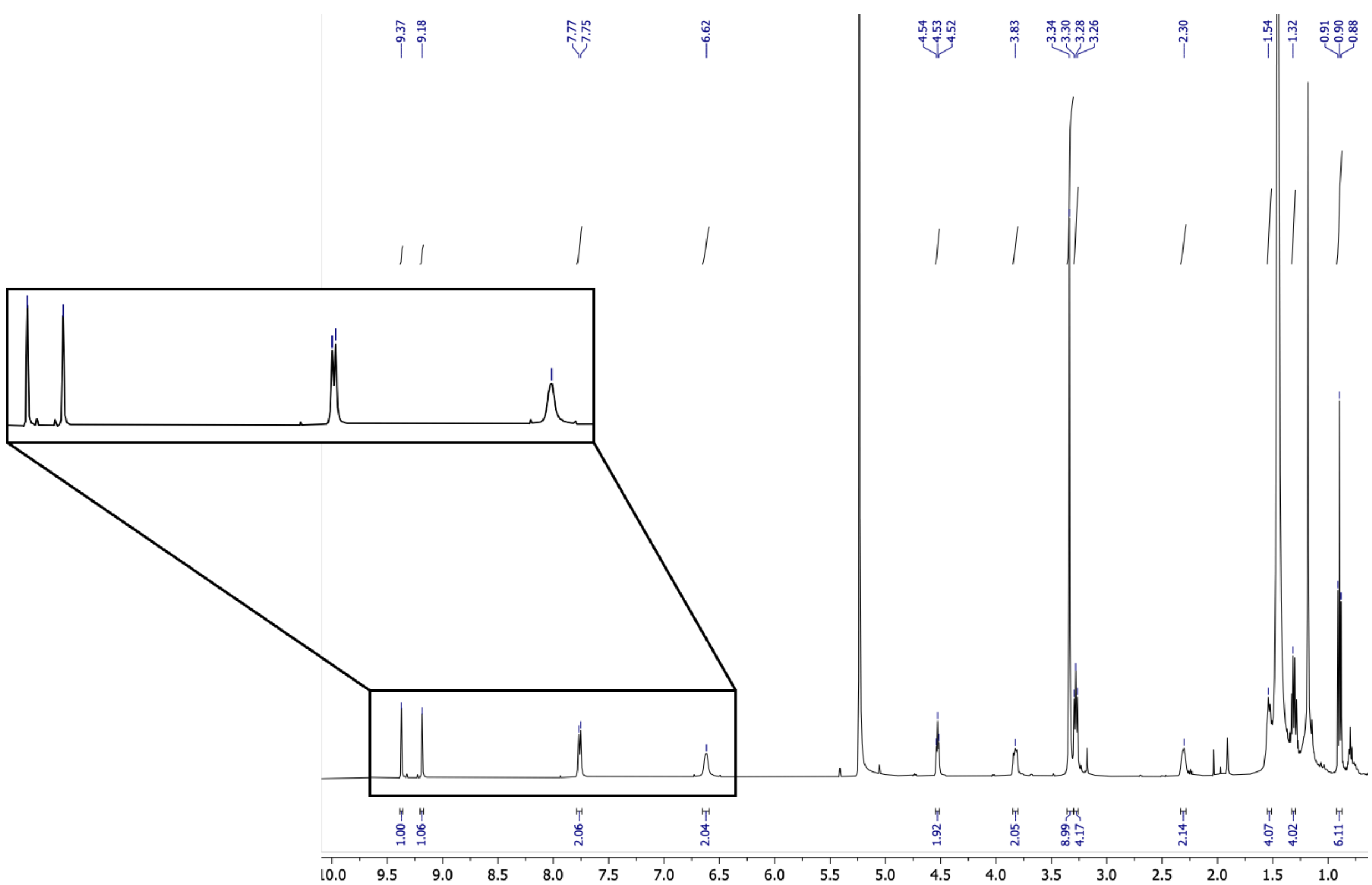

Figure S5: ^{1}H NMR of FlatTz-P

Section 2.2: MALDI-TOF MS

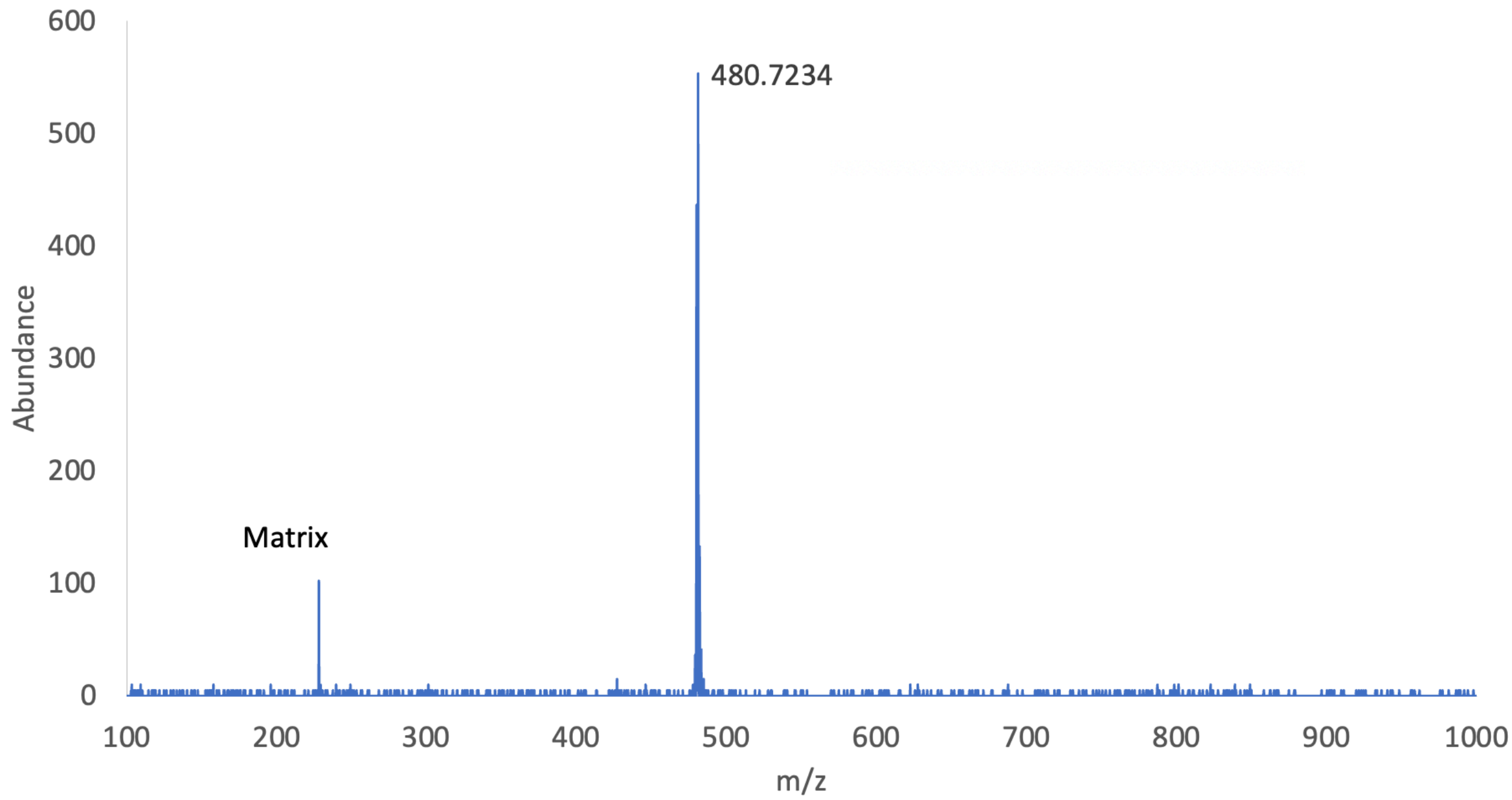

Figure S6: MALDI-TOF MS of TwistTz-M^+

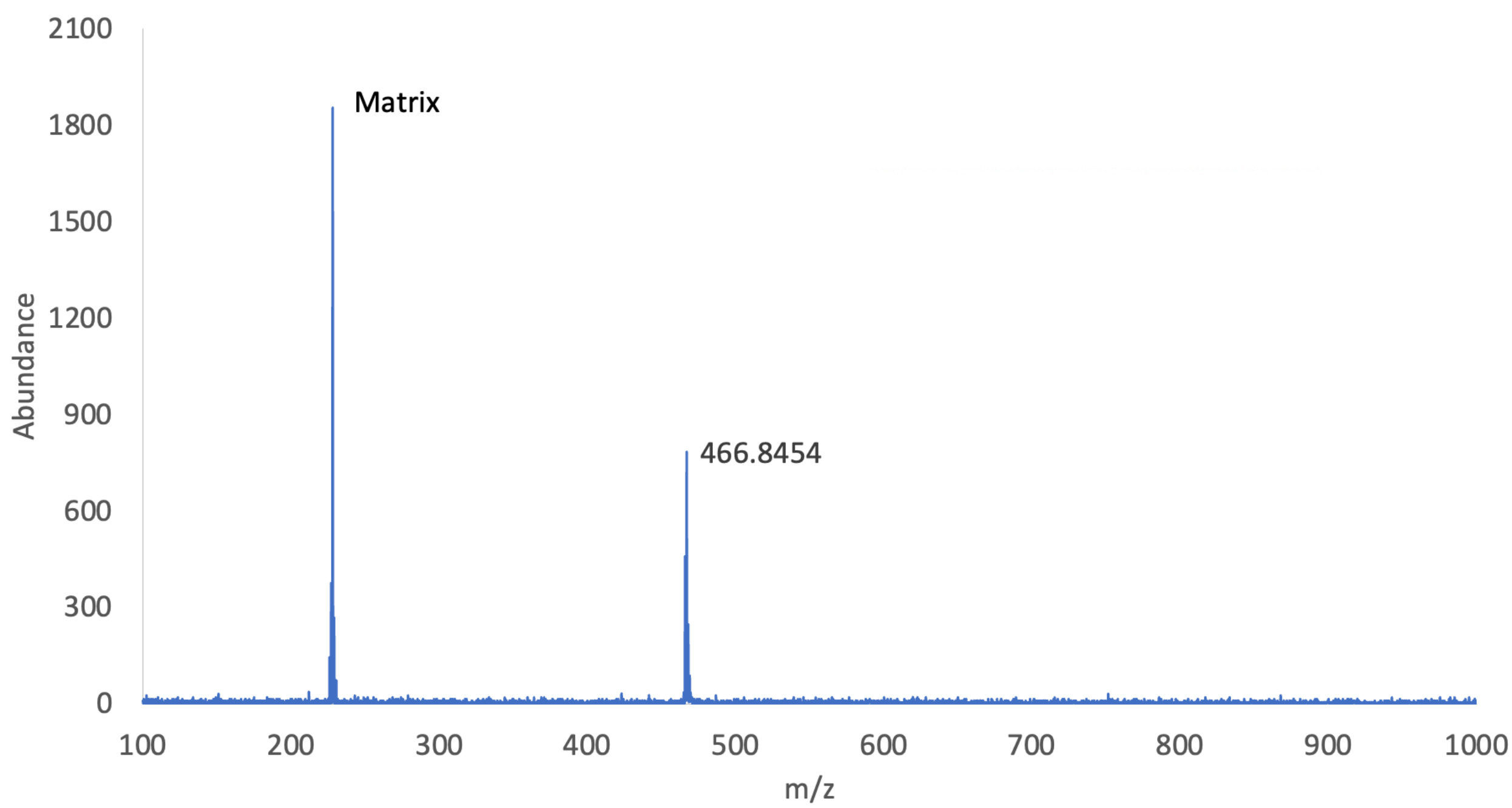

Figure S7: MALDI-TOF MS of TwistTz-A^+

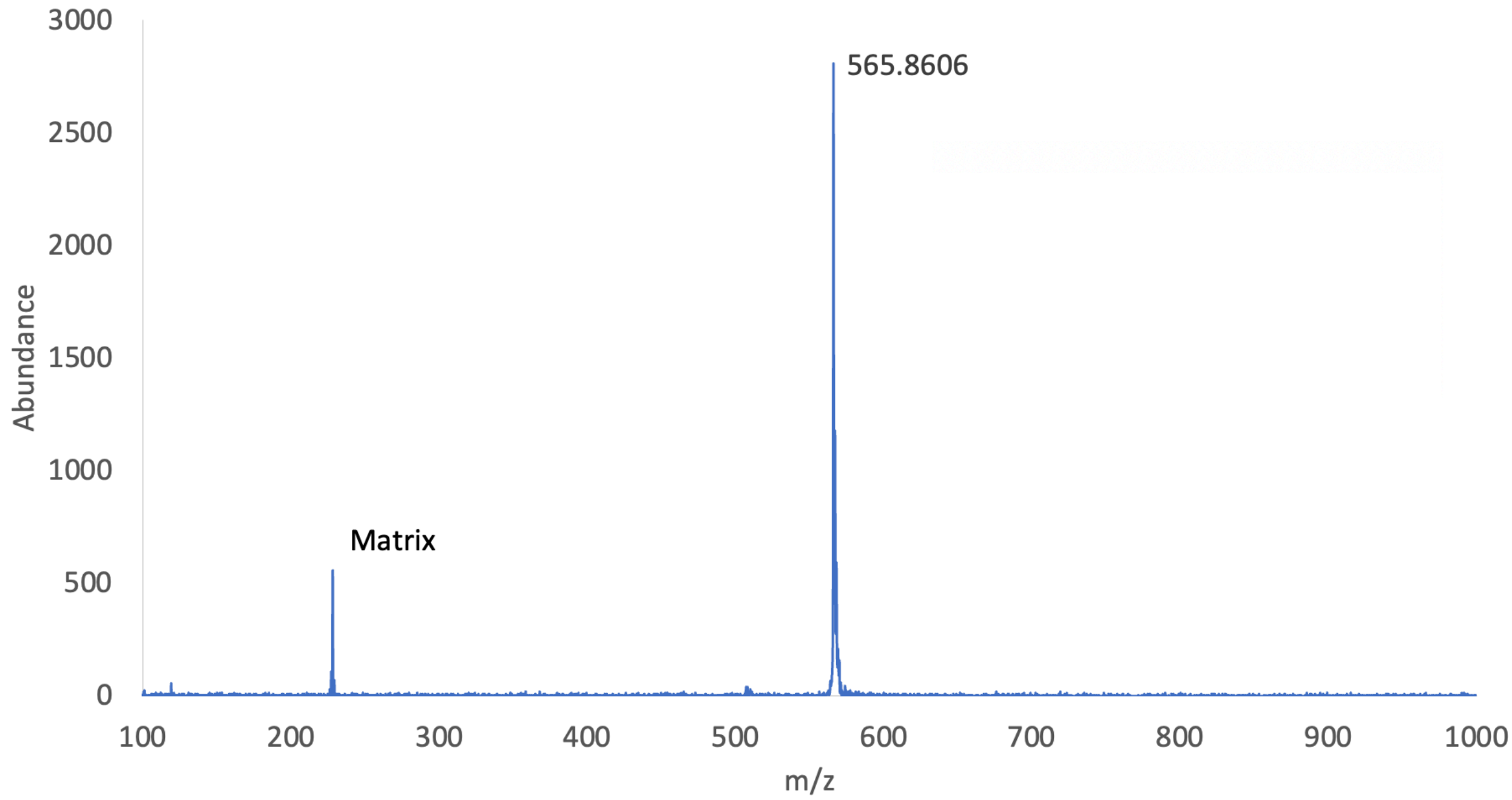

Figure S8: MALDI-TOF MS of TwistTz-P^+

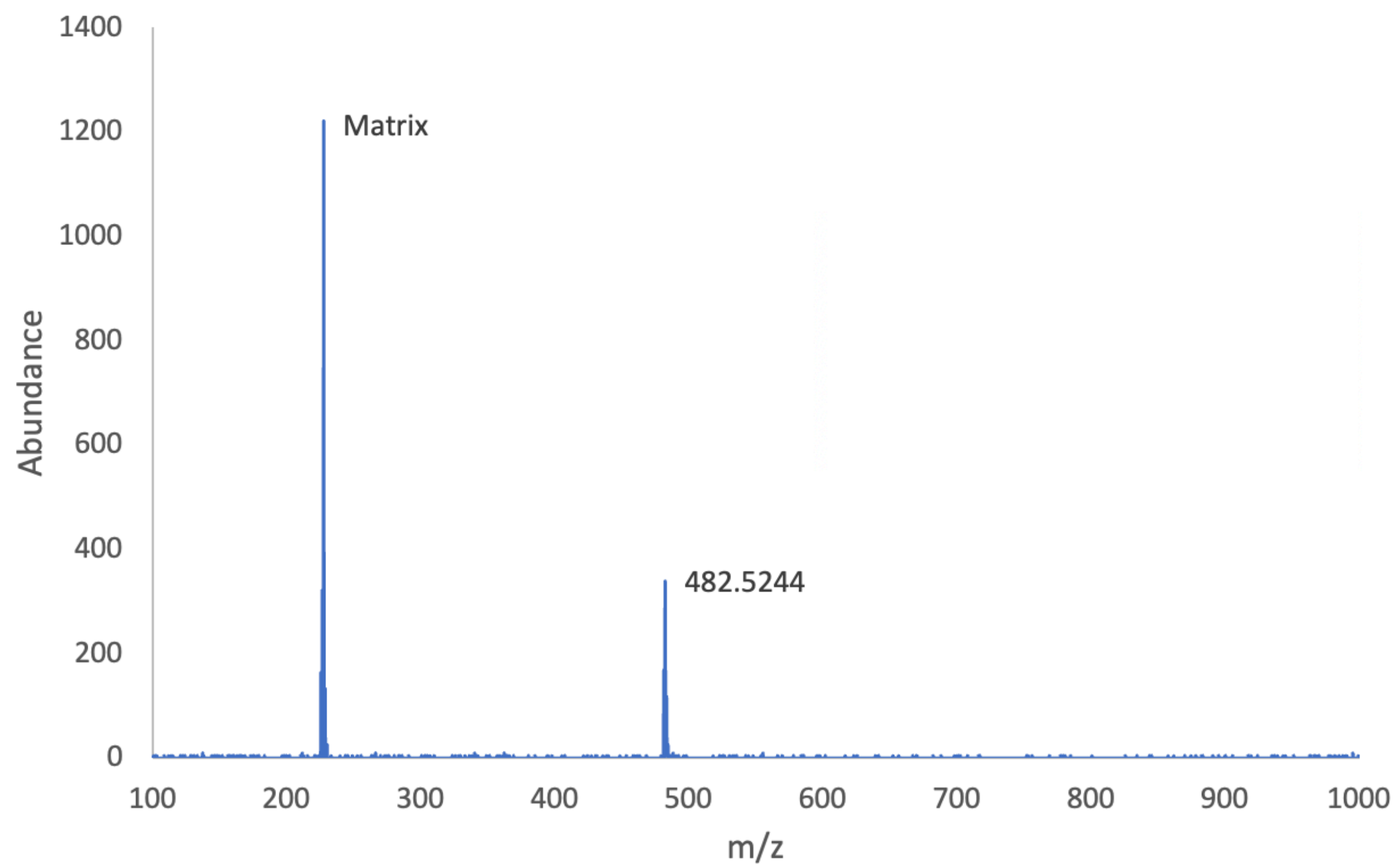

Figure S9: MALDI-TOF of FlatTz-M^+

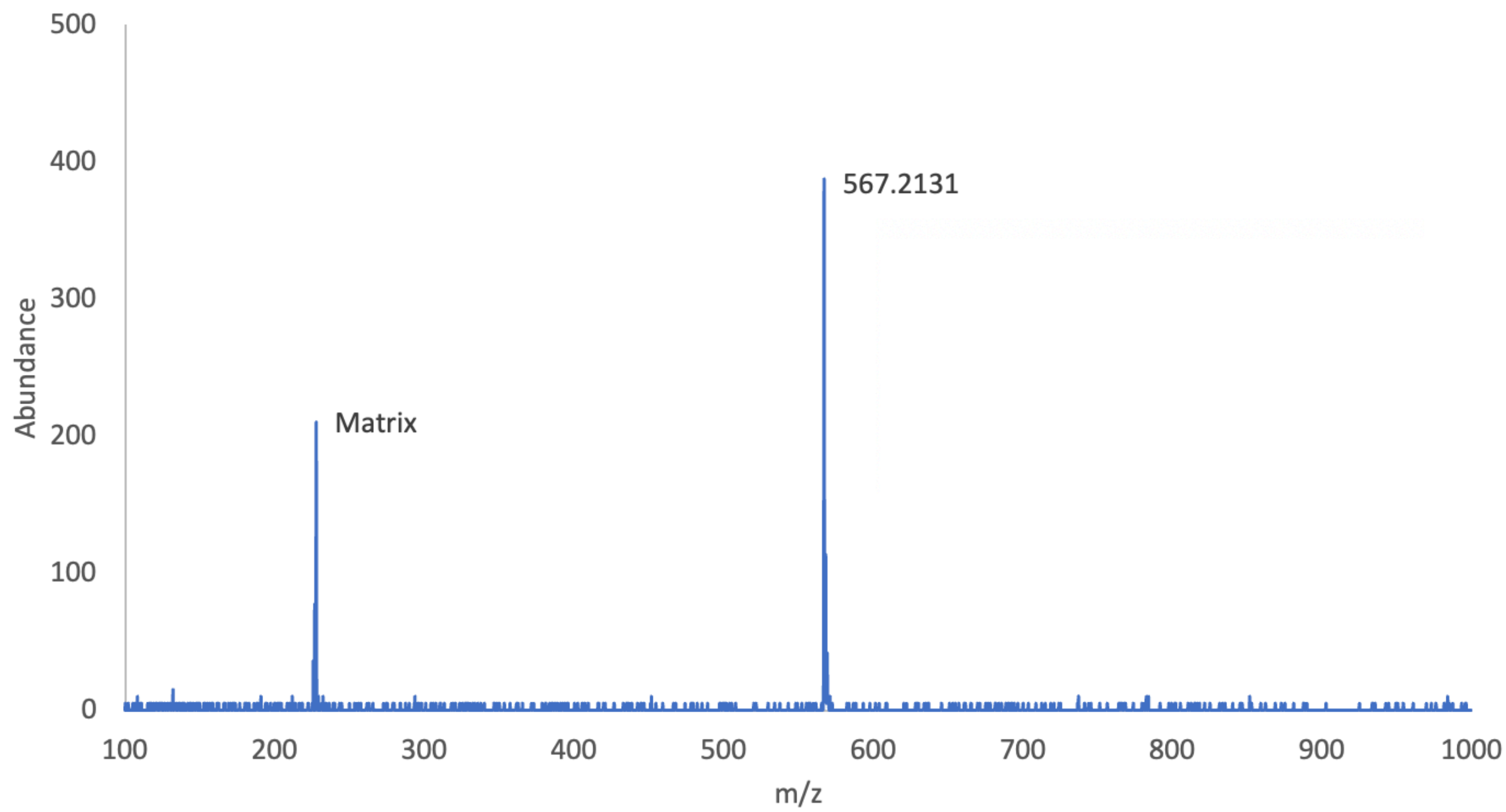

Figure S10: MALDI-TOF of FlatTz-P^+

Section 3: Solvent Sensitivity

Section 3.1: Emission

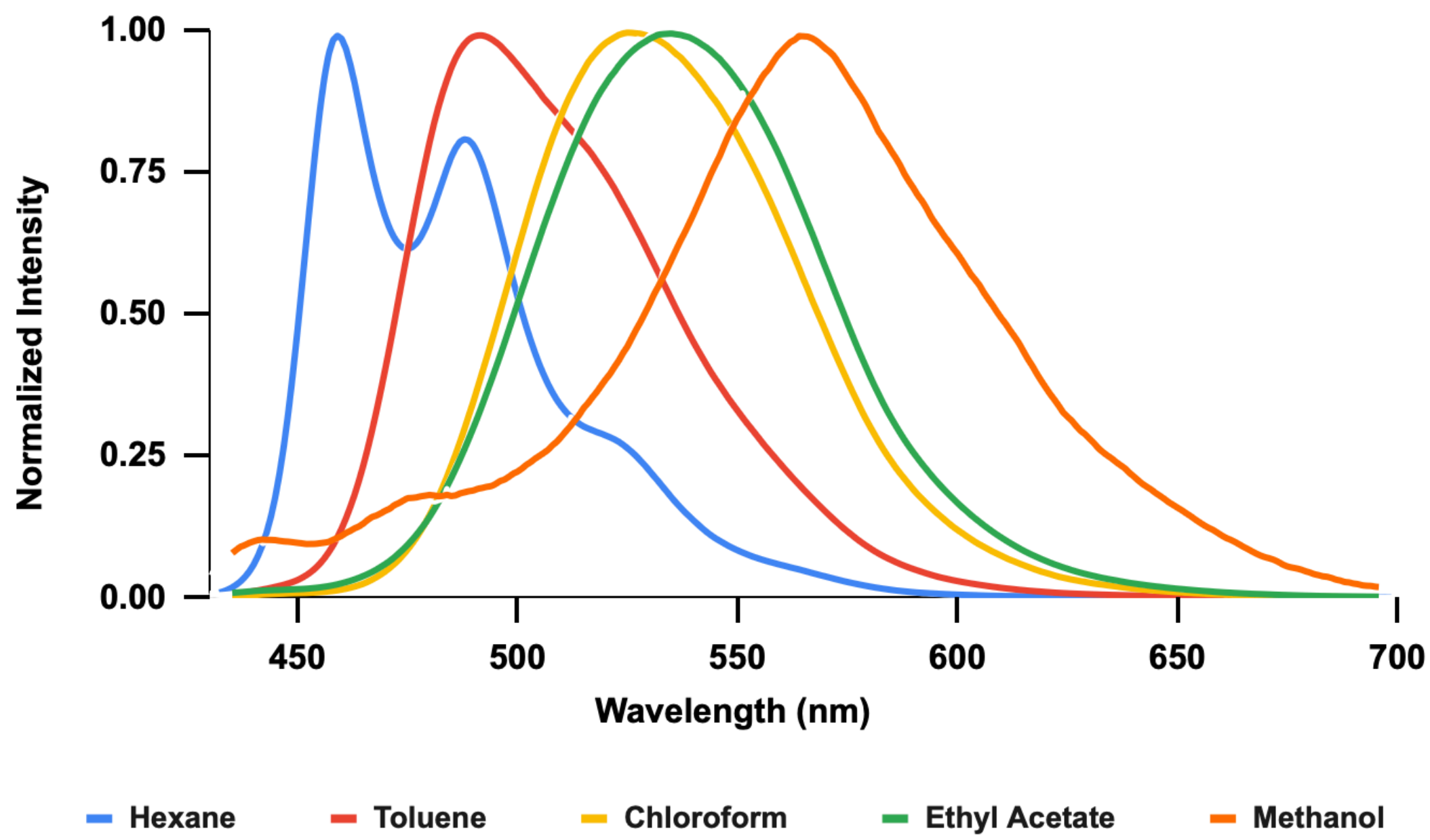

Figure S11: Normalized emission of TwistTz-M in different solvents (415 nm excitation).

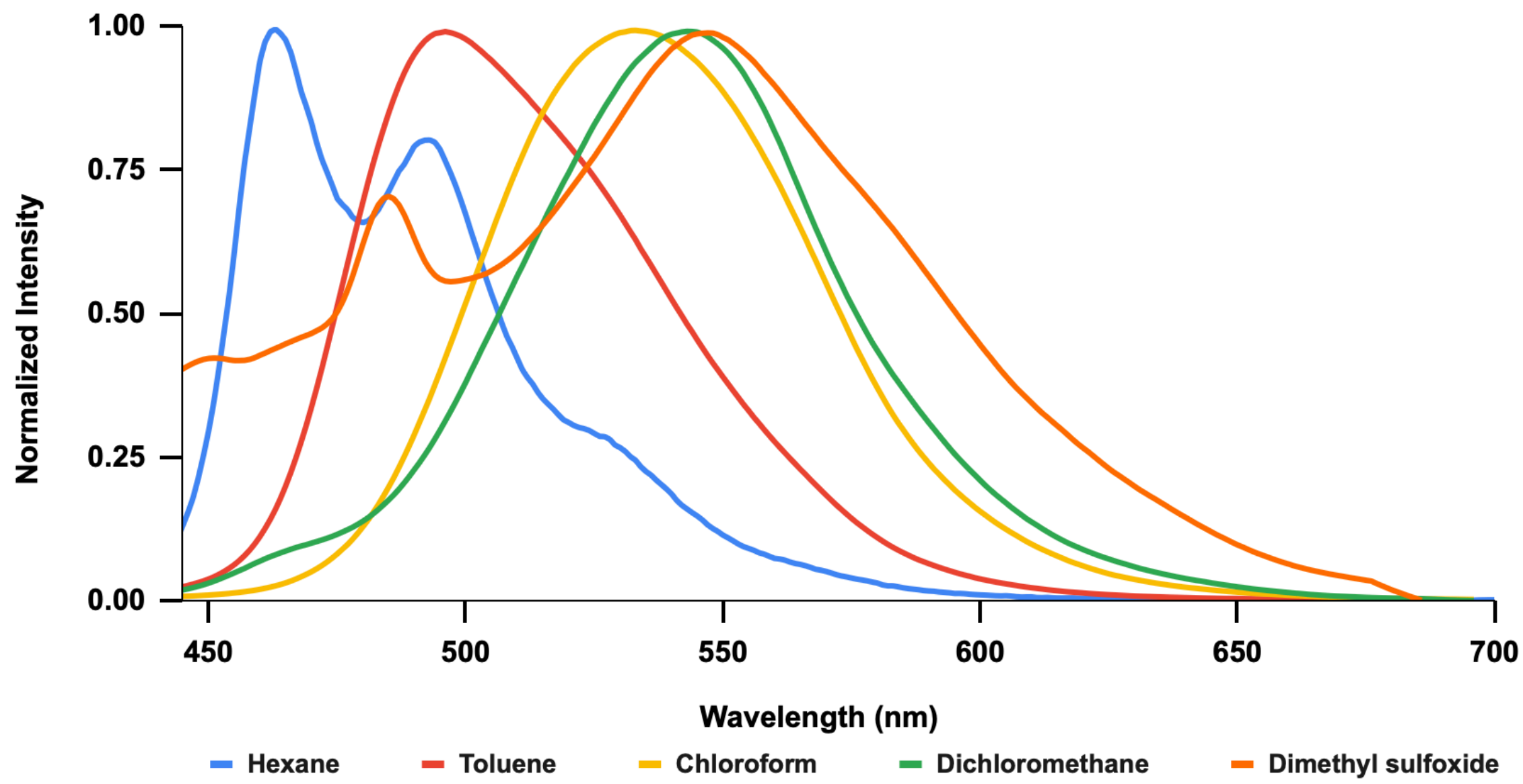

Figure S12: Normalized emission of TwistTz-A in different solvents (415 nm excitation).

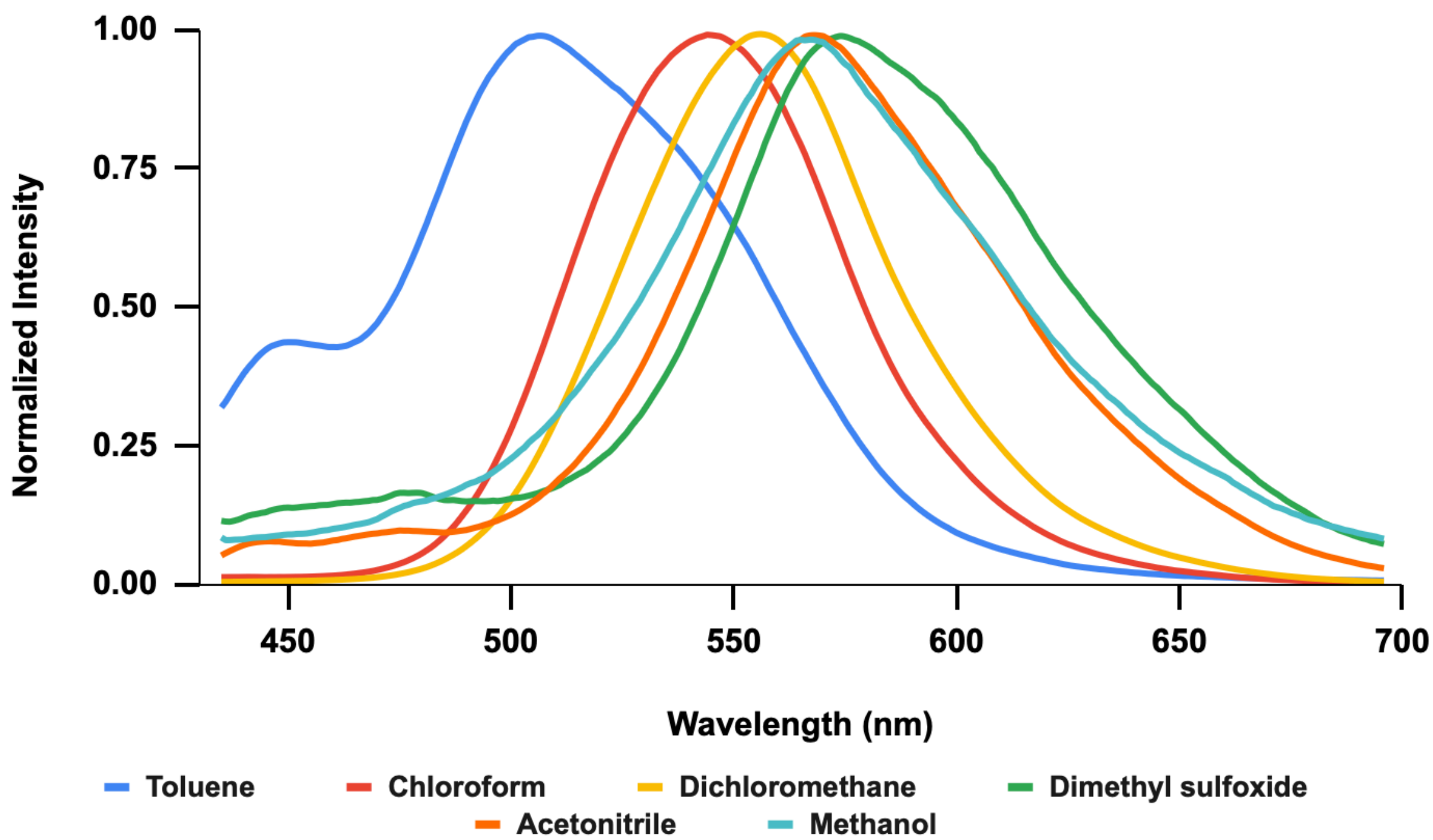

Figure S13: Normalized emission of TwistTz-P in different solvents (415 nm excitation).

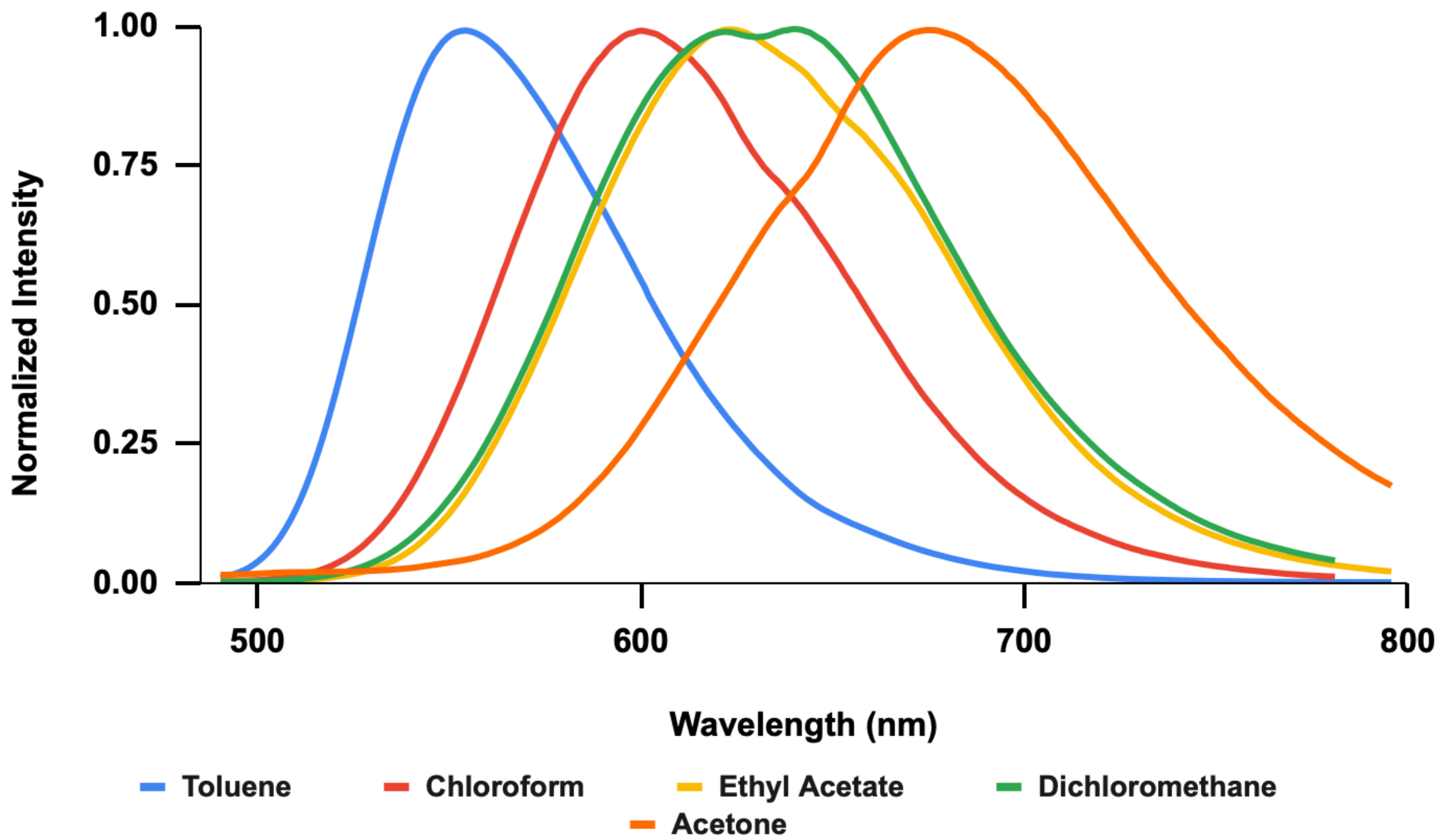

Figure S14: Normalized emission of FlatTz-M in different solvents

PC45

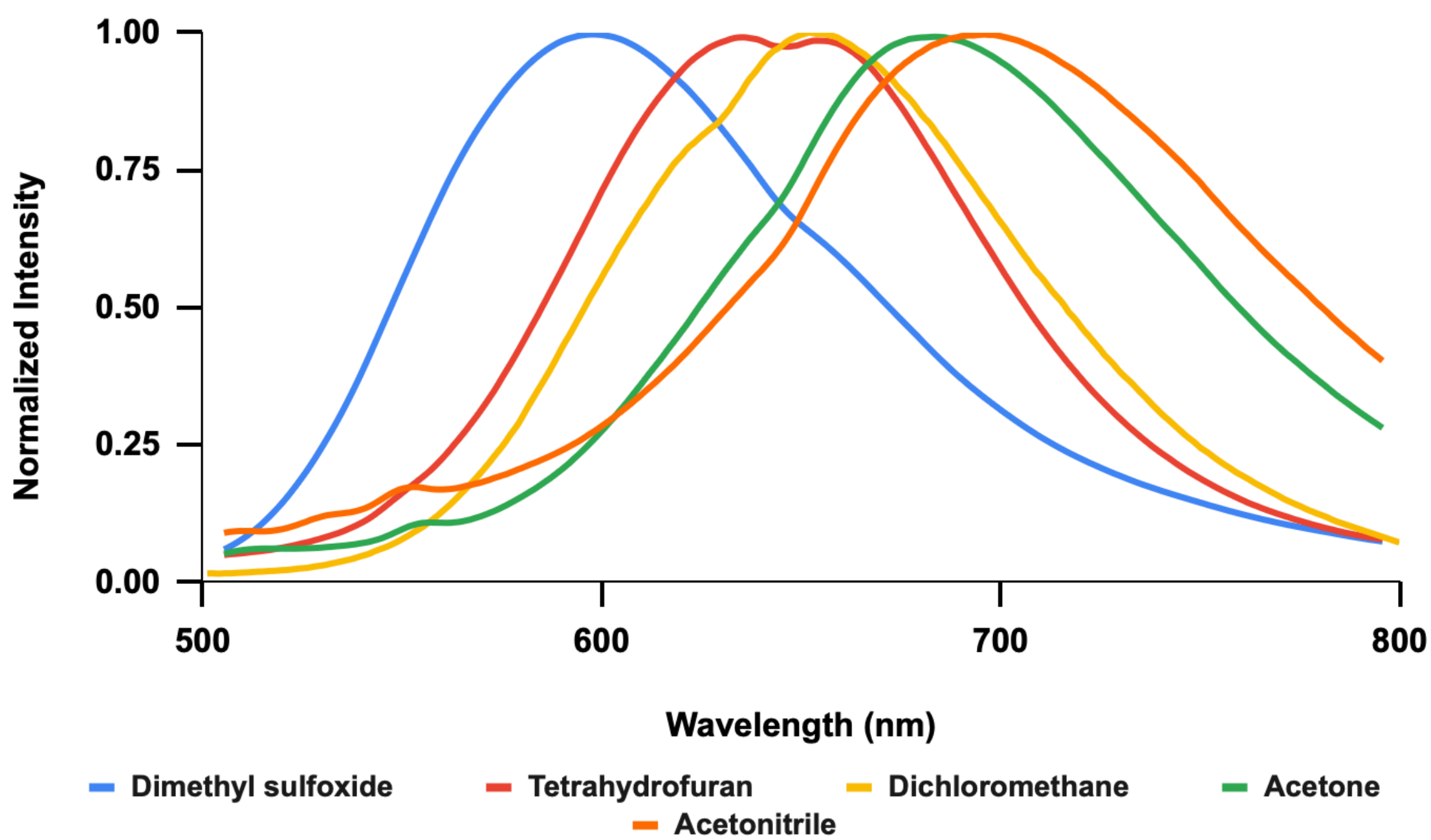

Figure S15: Normalized emission of FlatTz-P in different solvents

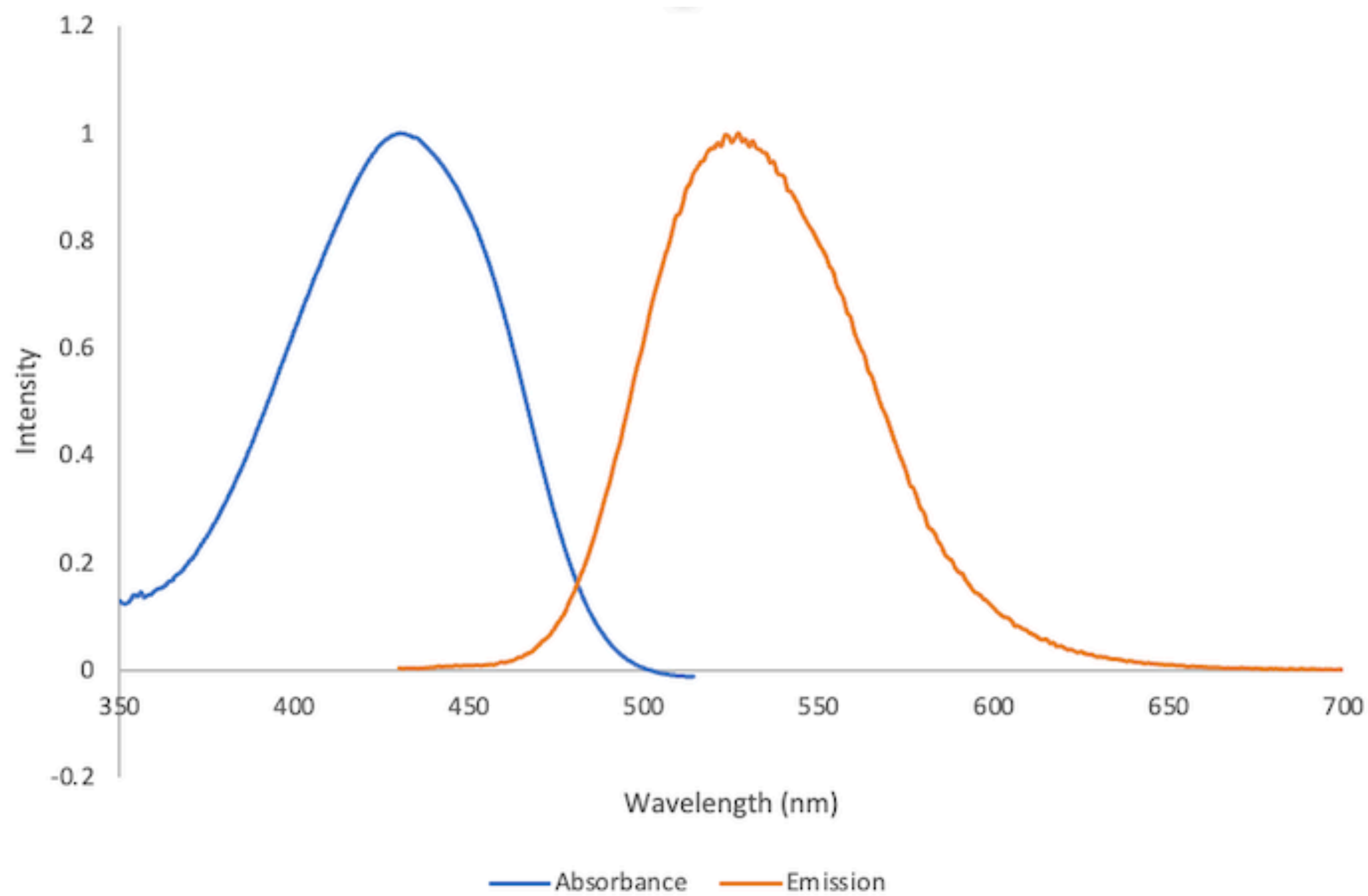

Figure S16: Normalized abs/em spectra of TwistTz-M in Chloroform.

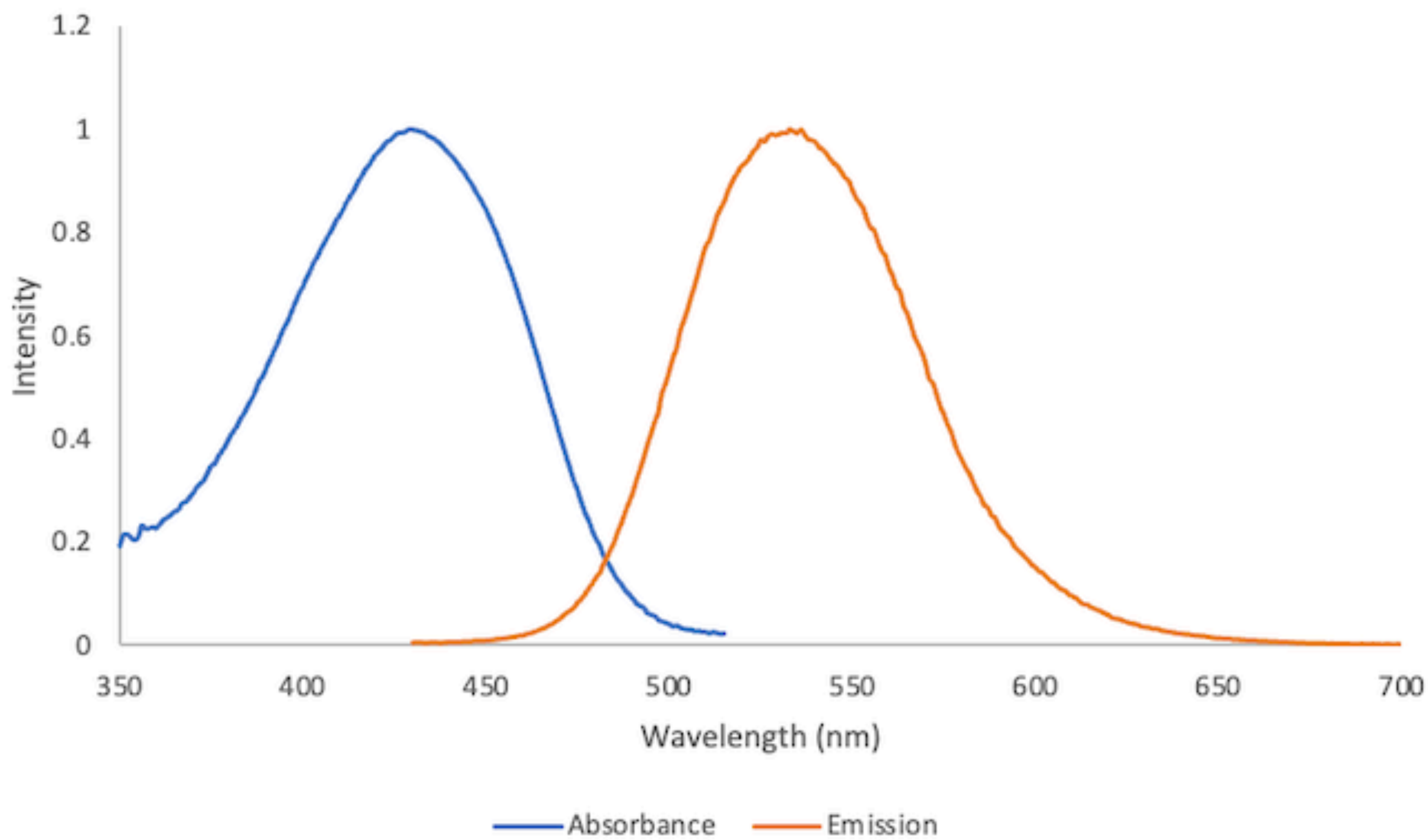

Figure S17: Normalized abs/em spectra of TwistTz-A in Chloroform

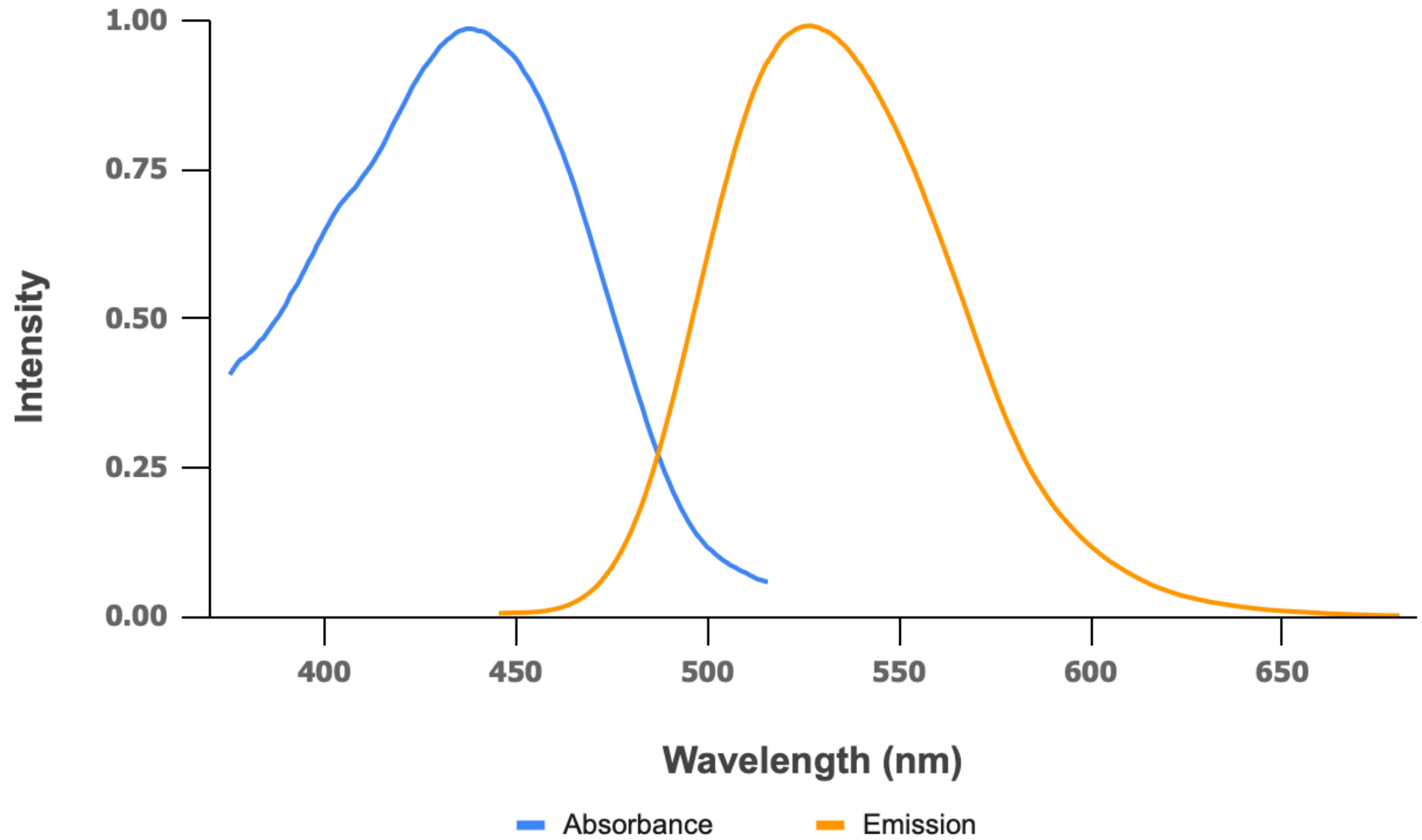

Figure S18: Normalized abs/em spectra of TwistTz-P in Chloroform

Section 3.2: Fluorescence Lifetimes

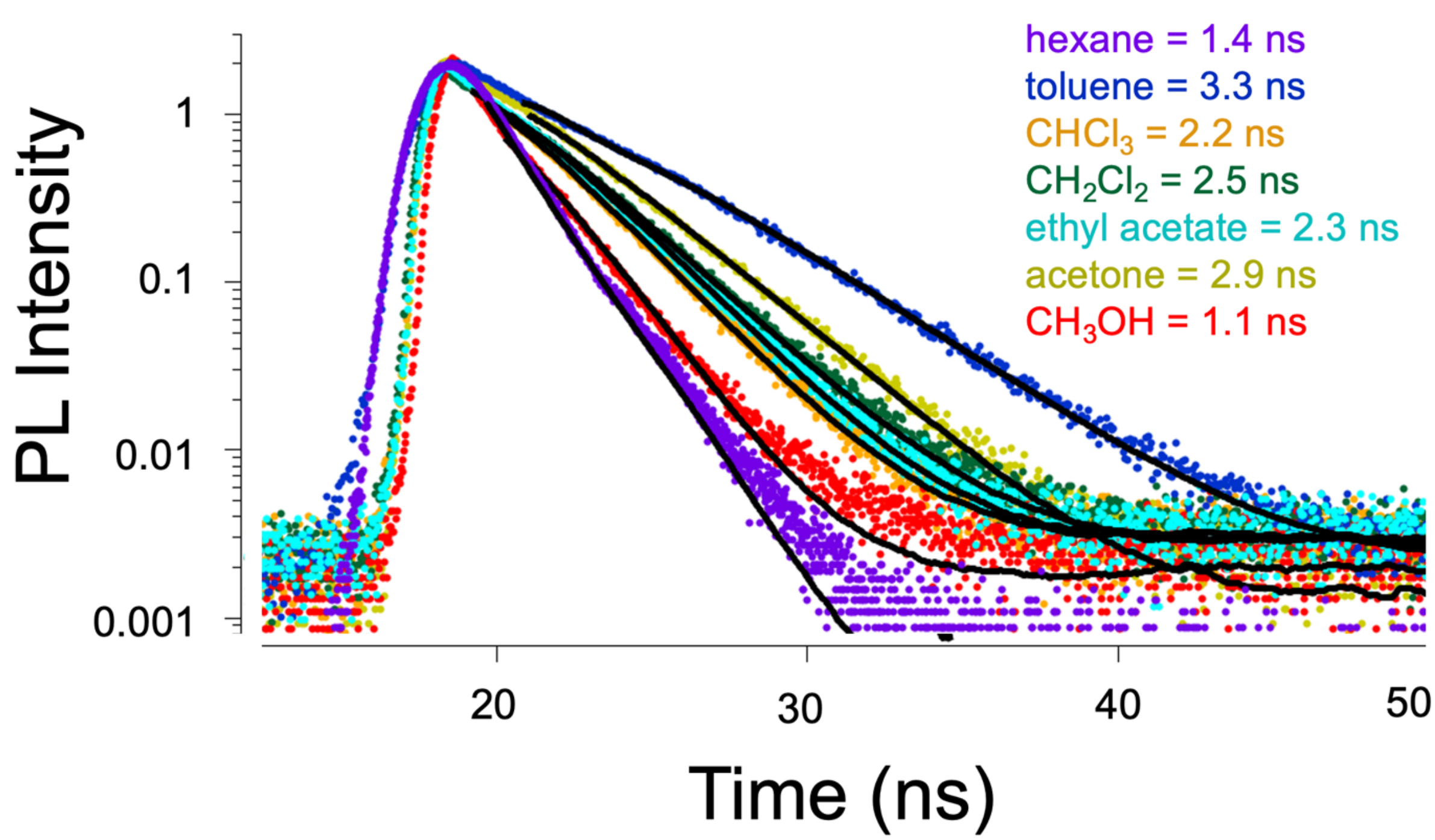

Figure S19: Fluorescence lifetime plots (405 nm excitation) of TwistTz-M in various solvents.

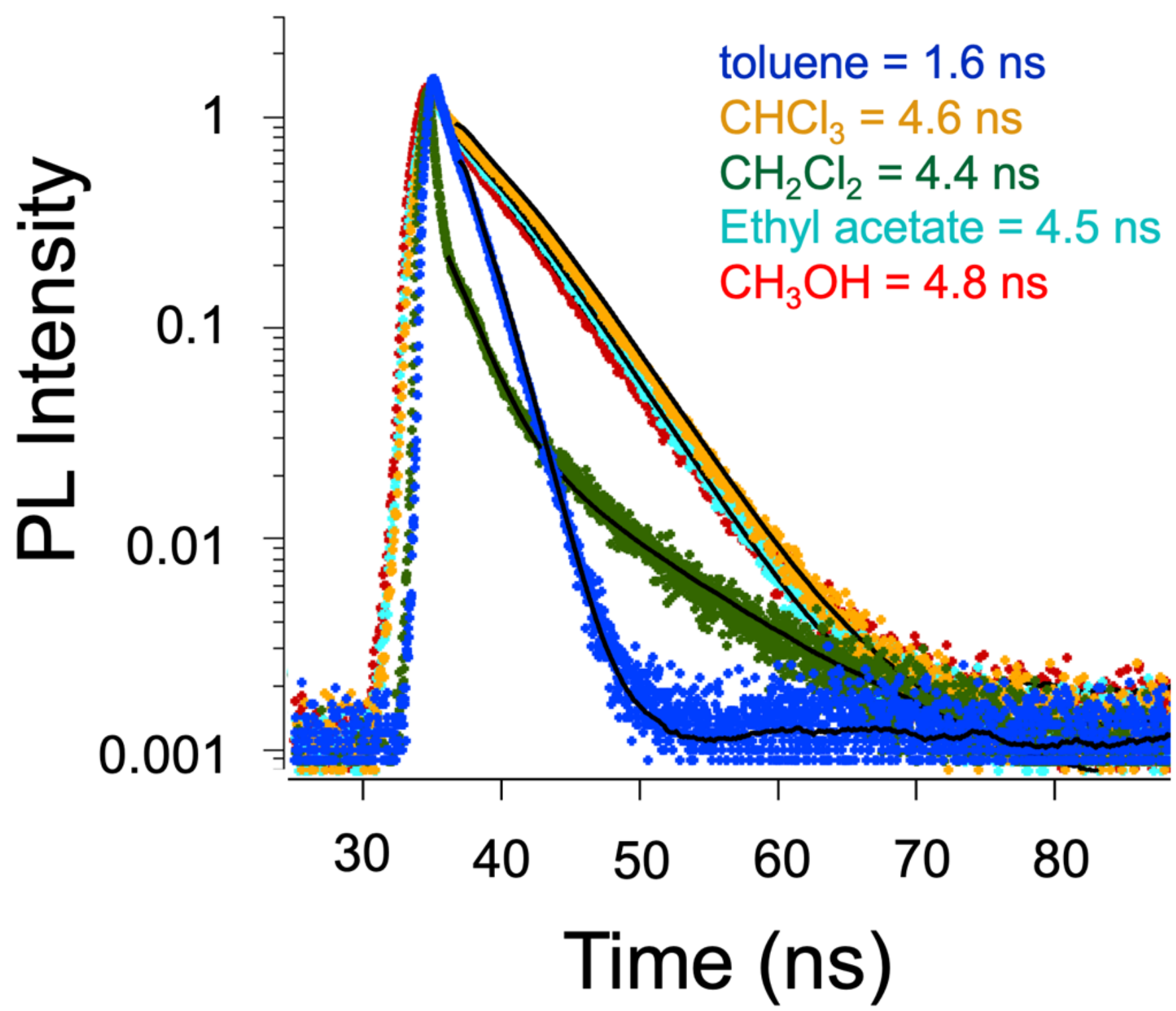

Figure S20: Fluorescence lifetime plots (405 nm excitation) of TwistTz-A in various solvents.

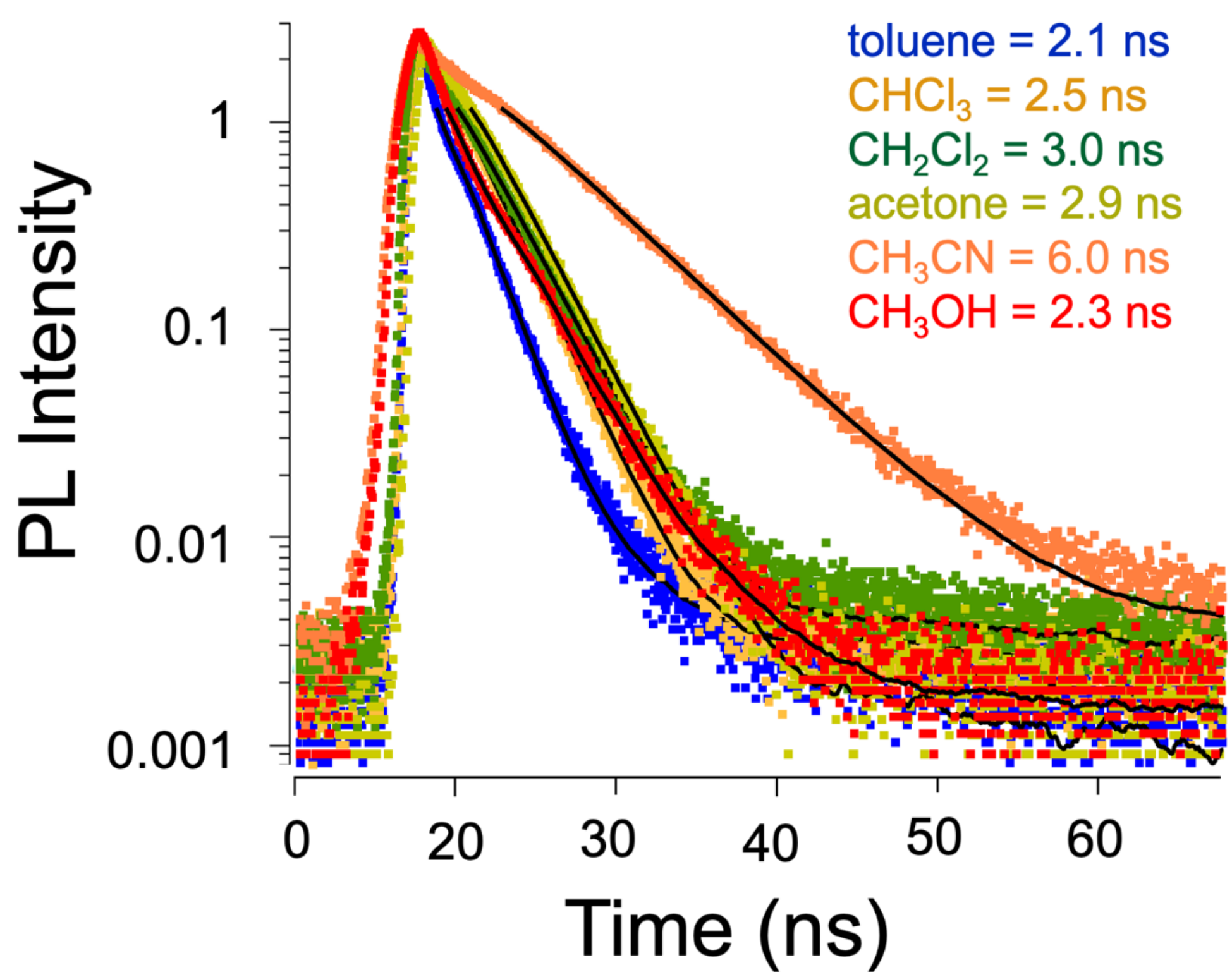

Figure S21: Fluorescence lifetime plots (405 nm excitation) of TwistTz-P in various solvents.

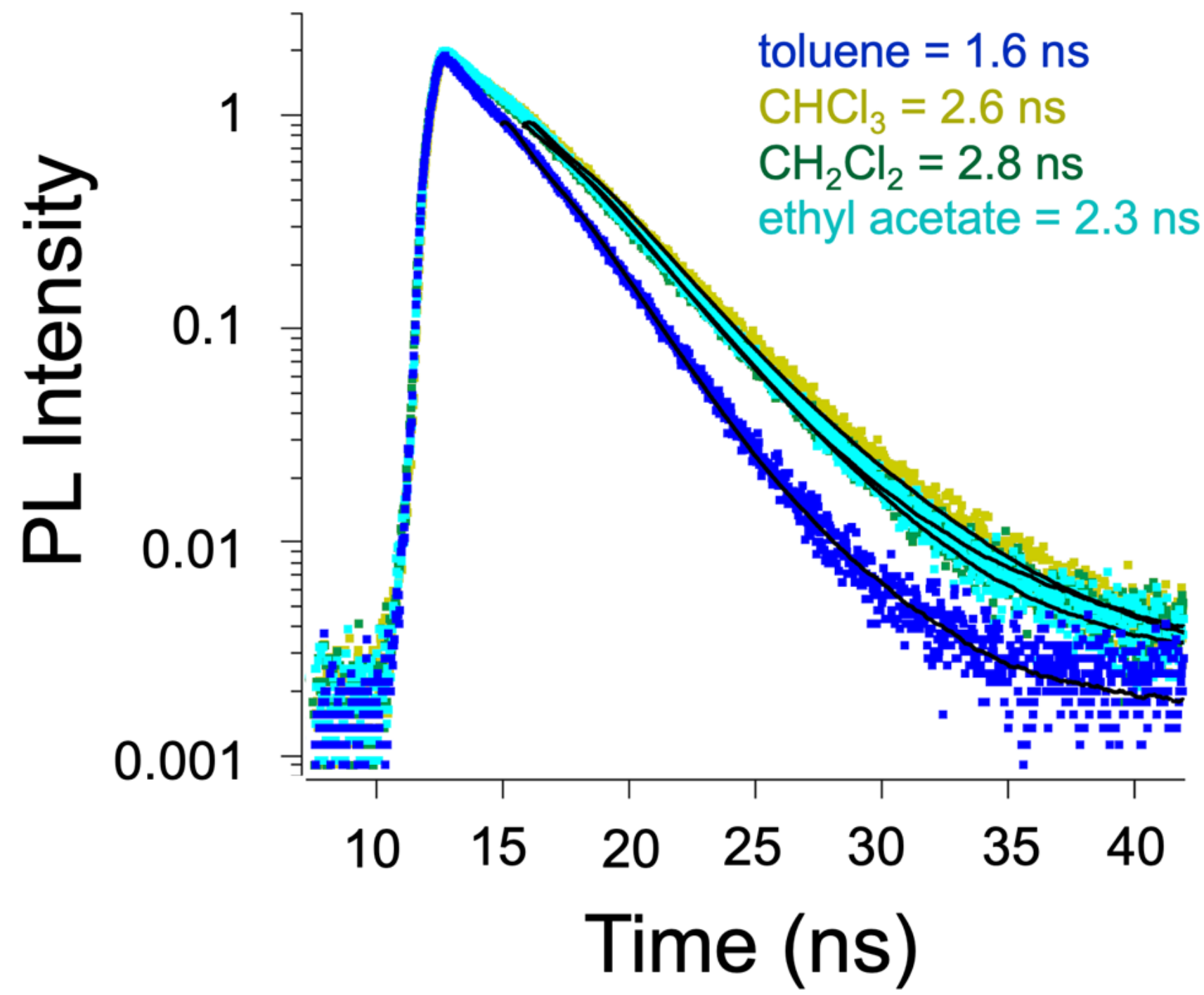

Figure S22: Fluorescence lifetime plots (405 nm excitation) of FlatTz-M in various solvents.

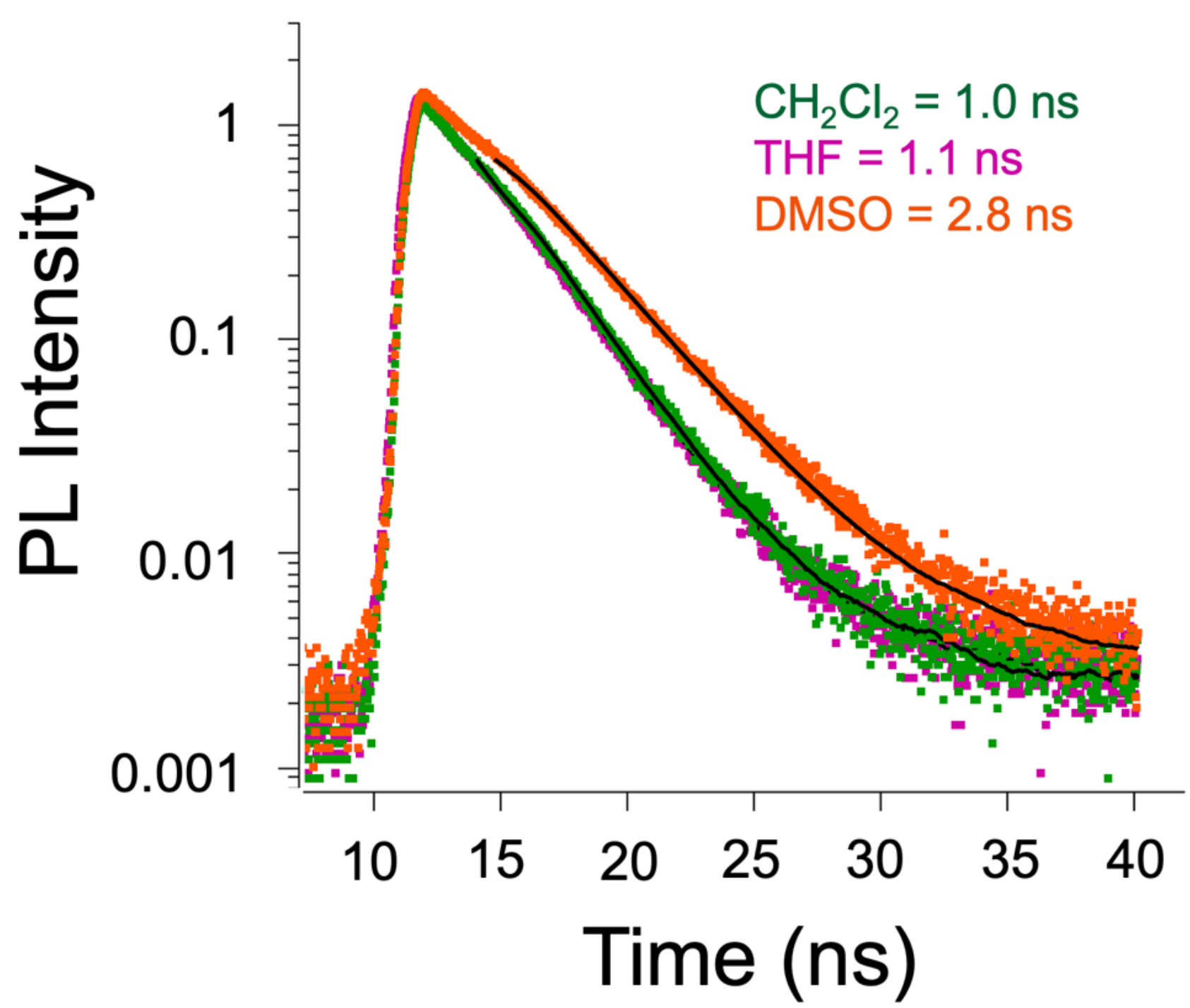

Figure S23: Fluorescence lifetime plots (405 nm excitation) of FlatTz-P in various solvents.

Section 3.3: Quantum Yield and Molar Absorptivity

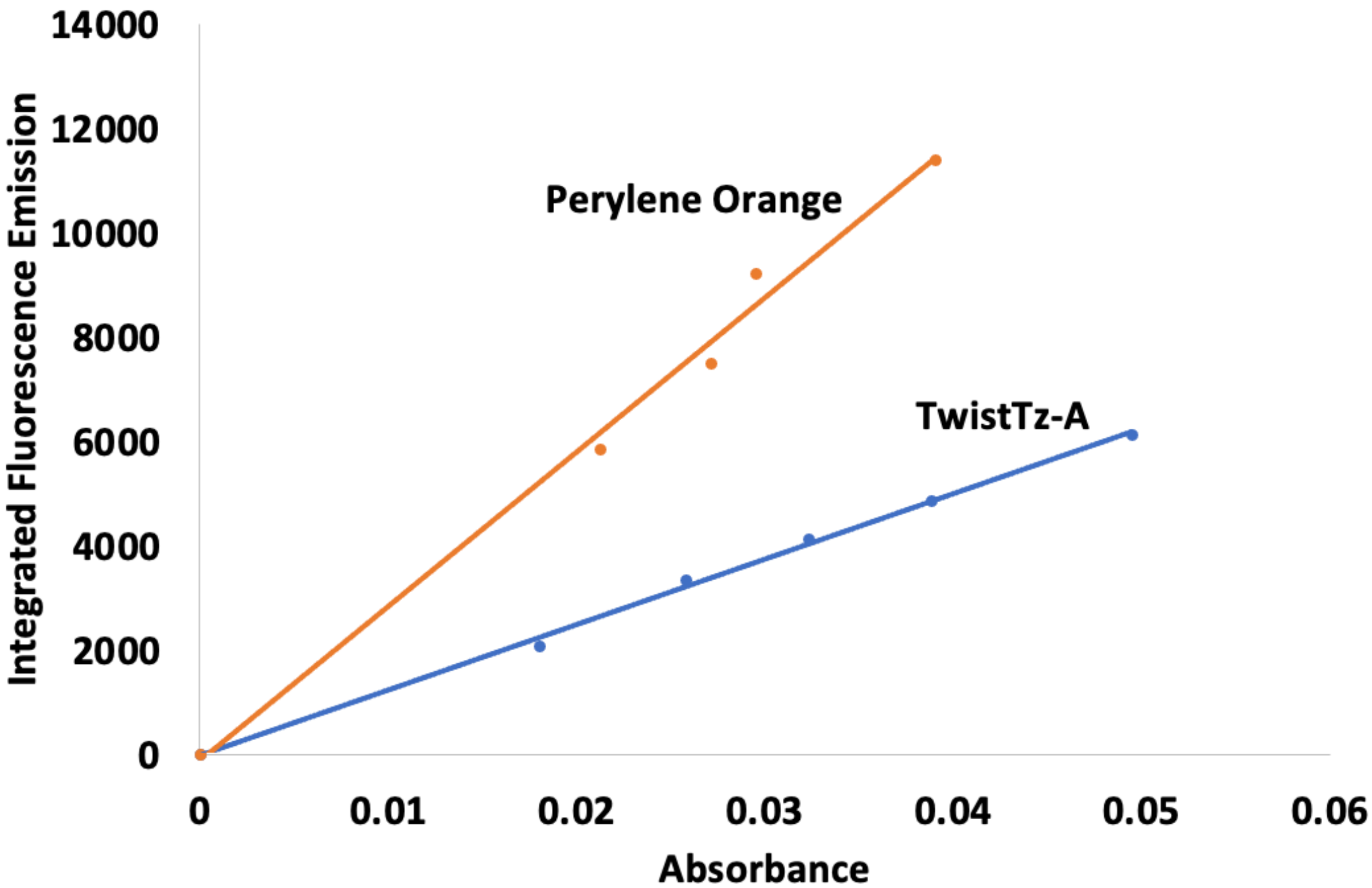

Figure S24: Example of relative fluorescence quantum yield with TwistTz-A in DCM and reference perylene orange in $CHCl_3$.

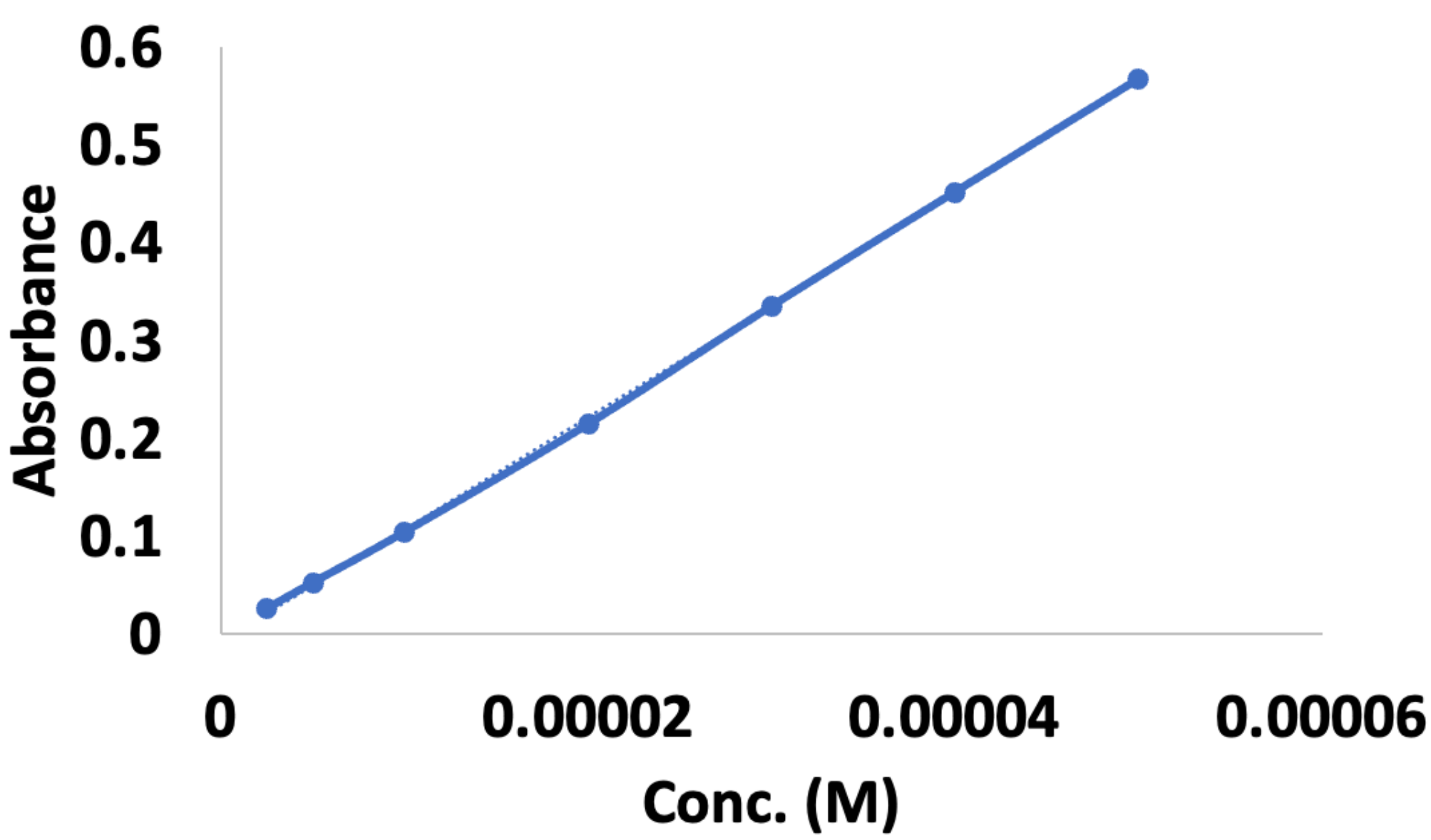

Figure S25: Example of molar absorptivity with TwistTz-M in MeOH.

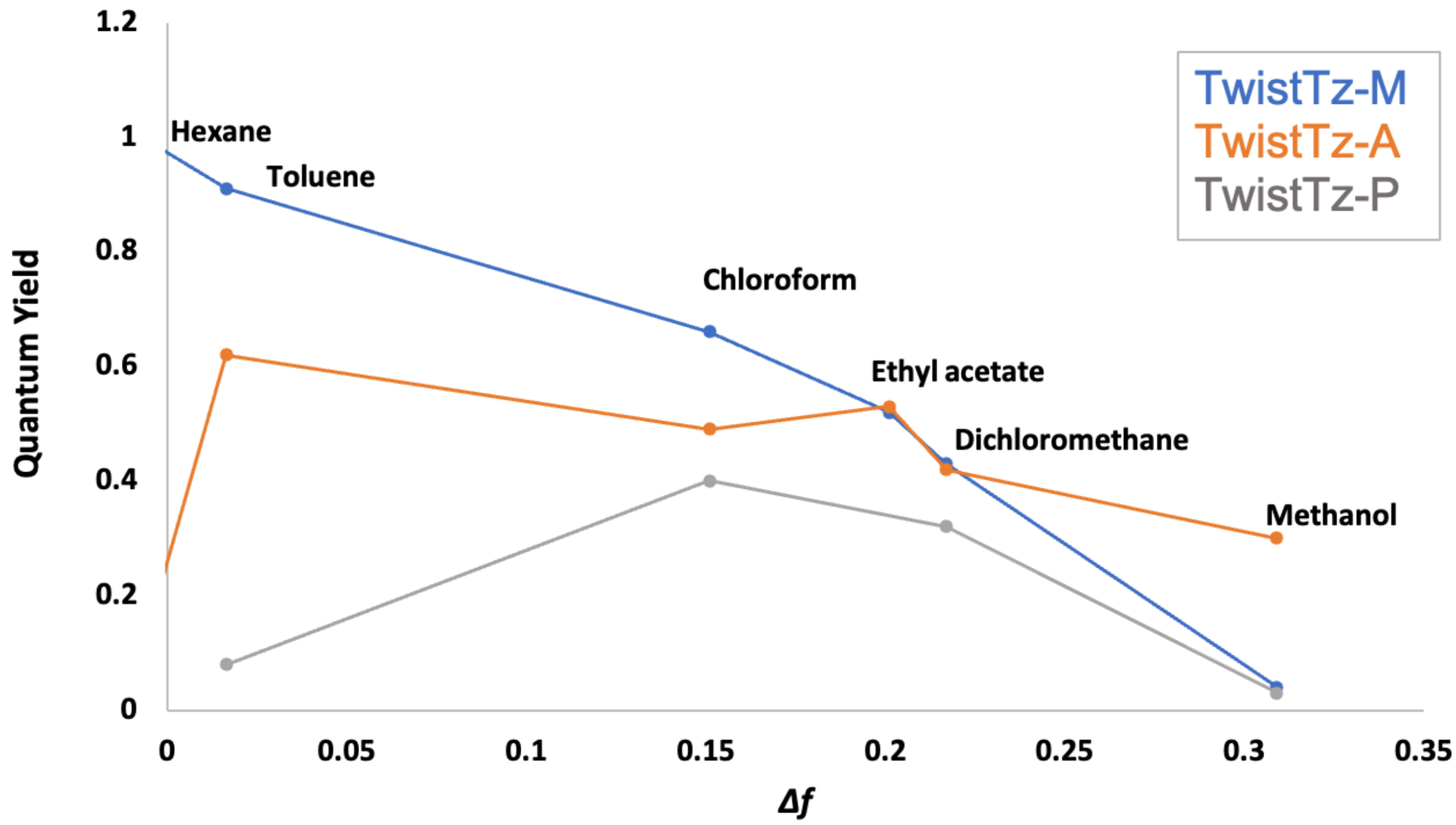

Figure S26: QY of asymmetric TwistTz-M, A, and P dyes with increasing polarizability.

Section 3.4: Optical Properties

Table S1. Optical properties of Asym-TTz dyes in various solvents

Compound	Solvent	Abs. (nm)	Abs. (eV)	Em. (nm)	Em. (eV)	Stokes shift (eV)	Quantum yield (Φ_F)	Fluorescence lifetime (ns)	Polarizability (Δf)	Radiative rate (s^{-1})	Non-radiative rates (s^{-1})
TwistTz-M	Hexane	417	2.98	454	2.74	0.24	0.98	1.4	-0.0017	6.8×10^8	0.1×10^8
	Toluene	426	2.92	489	2.54	0.38	0.91	3.3	0.0164	2.7×10^8	0.3×10^8
	$CHCl_3$	430	2.89	525	2.37	0.52	0.66	2.2	0.1508	3.0×10^8	1.6×10^8
	EtOAc	422	2.95	534	2.33	0.62	0.52	2.3	0.2007	2.2×10^8	2.1×10^8
	DCM	427	2.91	545	2.28	0.63	0.43	2.5	0.2171	1.7×10^8	2.2×10^8
	Acetone	428	2.90	560	2.22	0.68	0.20	2.9	0.2841	0.7×10^8	2.8×10^8
	MeOH	428	2.90	566	2.20	0.71	0.04	1.1	0.3088	0.4×10^8	9.1×10^8
TwistTz-A	Hexane	417	2.98	462	2.69	0.29	0.22	1.5	-0.0017	1.4×10^8	5.0×10^8
	Toluene	424	2.93	494	2.52	0.42	0.62	1.6	0.0164	3.8×10^8	2.3×10^8
	$CHCl_3$	429	2.90	533	2.33	0.57	0.49	4.6	0.1508	1.1×10^8	1.1×10^8
	EtOAc	424	2.93	529	2.35	0.58	0.53	4.5	0.2007	1.2×10^8	1.0×10^8
	DCM	434	2.86	552	2.25	0.61	0.42	4.4	0.2171	1.0×10^8	1.3×10^8
	MeOH	418	2.97	535	2.32	0.65	0.30	4.8	0.3088	0.6×10^8	1.5×10^8
TwistTz-P	Toluene	445	2.79	507	2.45	0.34	0.08	2.1	0.0164	0.4×10^8	4.5×10^8
	$CHCl_3$	437	2.84	545	2.28	0.56	0.40	2.5	0.1508	1.6×10^8	2.4×10^8
	THF	433	2.87	548	2.27	0.60	0.40	2.6	0.2058	1.5×10^8	2.3×10^8
	DCM	436	2.85	557	2.23	0.62	0.32	3.0	0.2171	1.1×10^8	2.3×10^8
	Acetone	431	2.88	565	2.20	0.68	0.20	2.9	0.2841	0.7×10^8	2.7×10^8
	MeCN	429	2.90	566	2.20	0.70	0.11	6.0	0.3062	0.2×10^8	1.5×10^8
	DMSO	442	2.81	576	2.16	0.65	0.07	2.4	0.2634	0.3×10^8	3.9×10^8
FlatTz-M	Toluene	471	2.64	552	2.25	0.39		1.6	0.0164		
	$CHCl_3$	486	2.56	618	2.01	0.55	0.51	2.6	0.1508	2.0×10^8	1.9×10^8
	EtOAc	467	2.66	618	2.01	0.65	0.46	2.3	0.2007	2.0×10^8	2.3×10^8
	DCM	481	2.58	654	1.90	0.68	0.25	2.8	0.2171	0.9×10^8	2.6×10^8
FlatTz-P	$CHCl_3$	495	2.51	645	1.93	0.58	0.23		0.1508		
	THF	474	2.62	635	1.96	0.66		1.1	0.2058		
	DCM	494	2.52	668	1.86	0.66	0.12	1.0	0.2171	1.1×10^8	8.4×10^8
	Acetone	476	2.61	681	1.83	0.79			0.2841		
	MeCN	473	2.63	694	1.79	0.84			0.3062		
	DMSO	484	2.57	597	2.08	0.49	0.01	2.8	0.2634	0.4×10^7	3.5×10^8

Section 3.5: Lippert-Mataga Plots

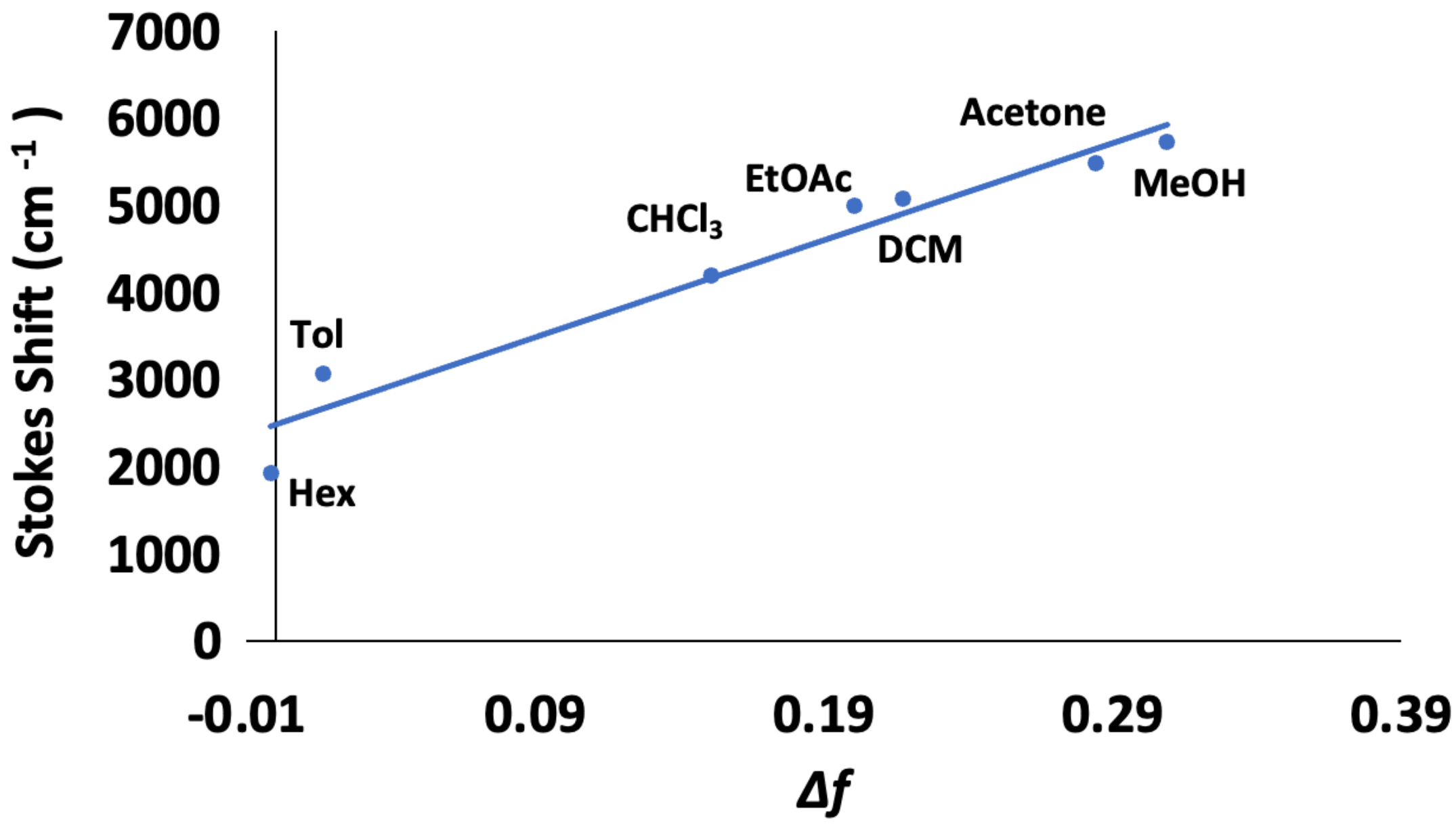

Figure S27: Lippert-Mataga plot of TwistTz-M.

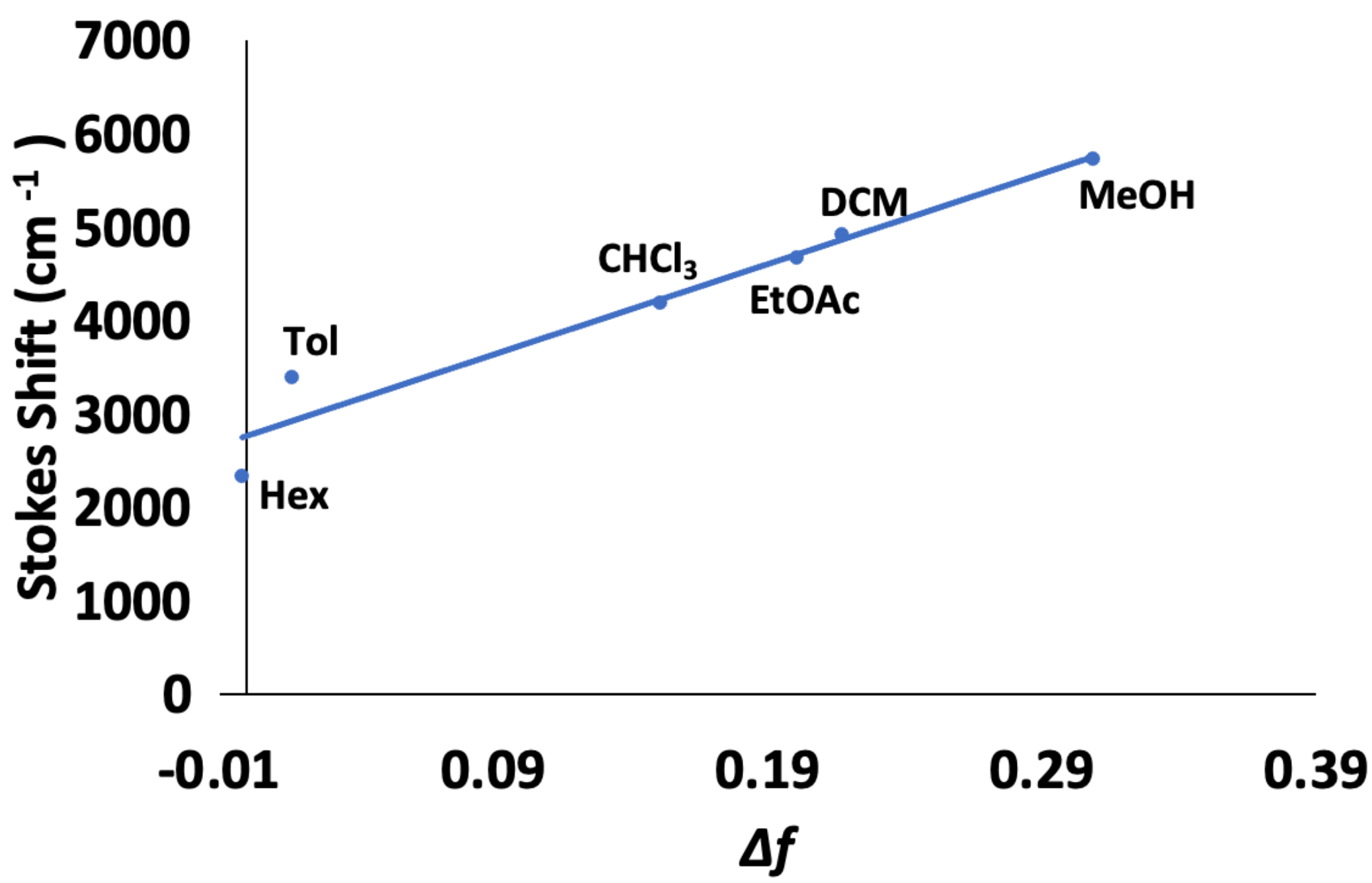

Figure S28: Lippert-Mataga plot of TwistTz-A.

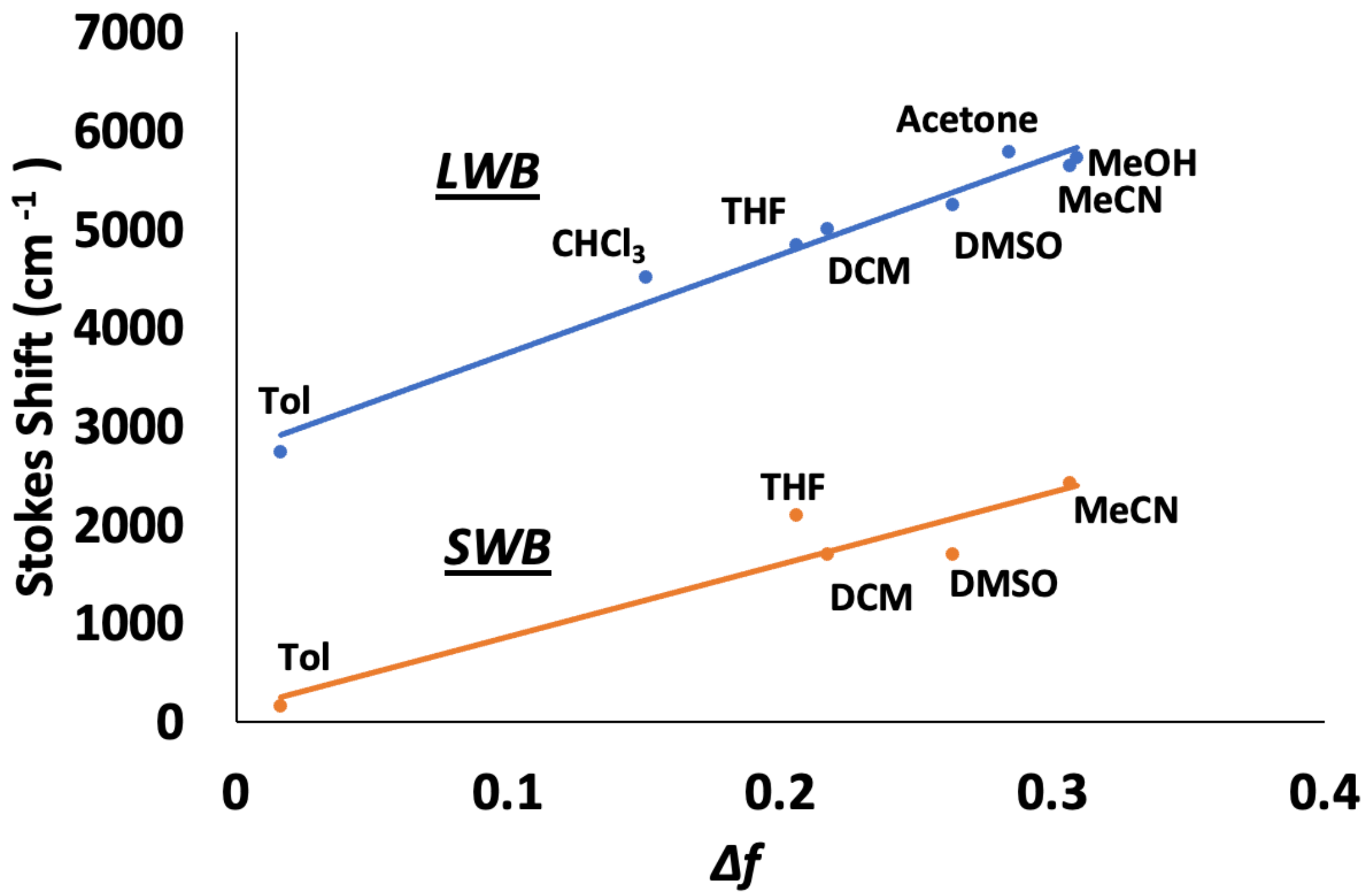

Figure S29: Lippert-Mataga plot of TwistTz-P showing LWB and SWB.

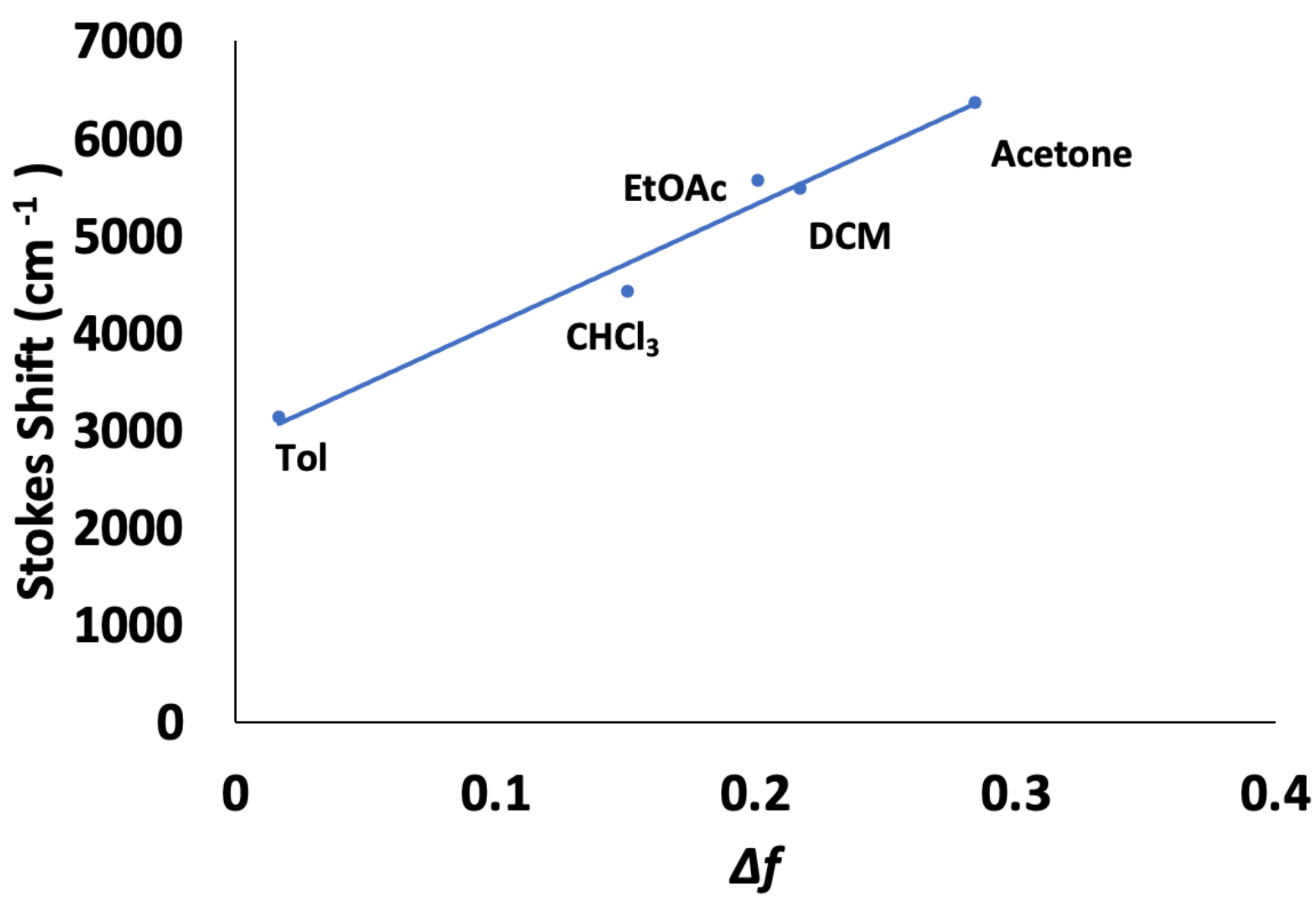

Figure S30: Lippert-Mataga plot of FlatTz-M.

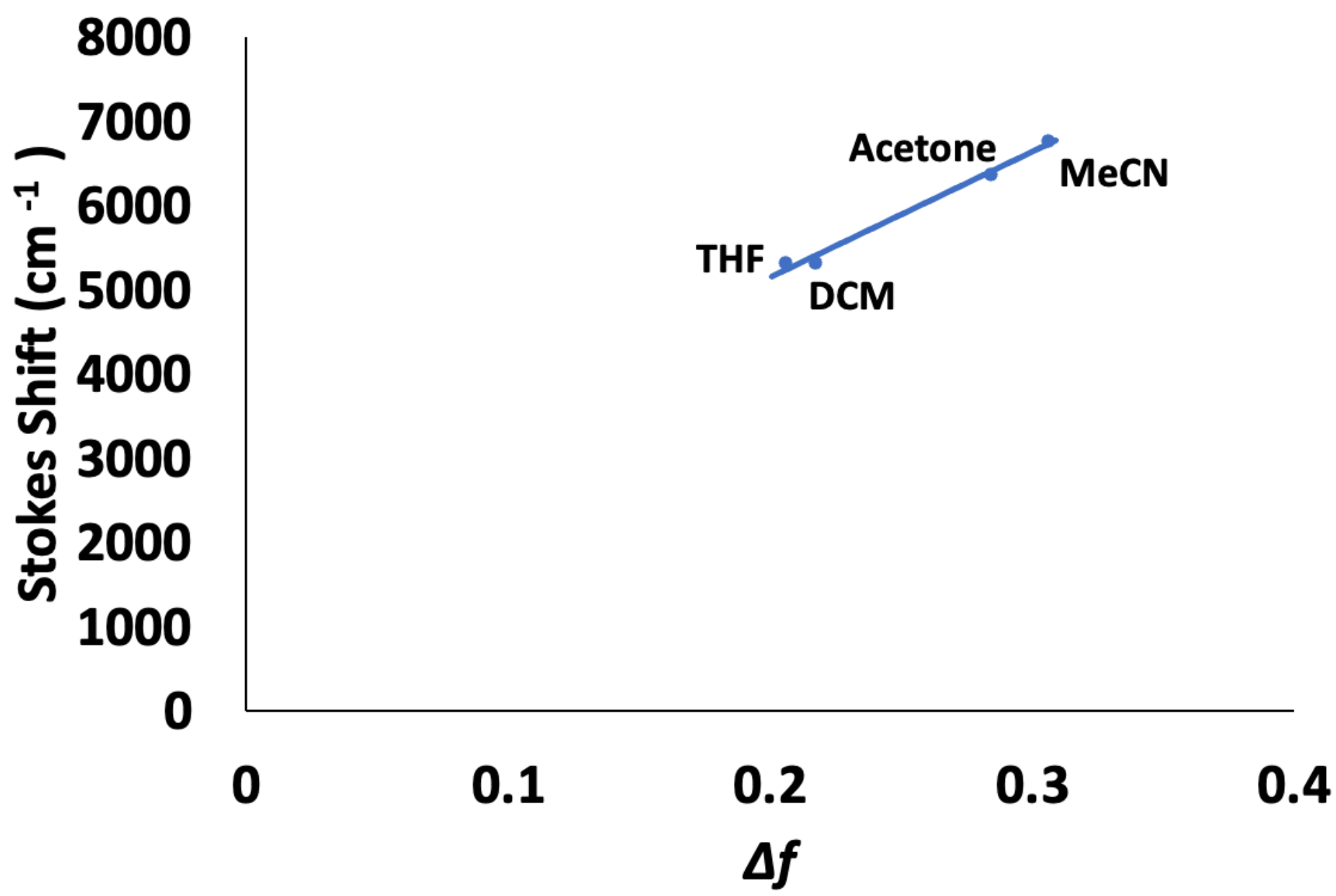

Figure S31: Lippert-Mataga plot of FlatTz-P.

Section 3.6: Polarizability

Table S2: Polarizability calculated from relative permittivity and refractive index of solvent. Constants obtained from sources at 20 or 25 °C

**Solvent**	**relative permittivity (ε)**	**index of refraction (η)**	**polarizability (Δf)**
Hexane	1.88	1.3749	-0.0017
DCM	8.93	1.4241	0.217
EtOAc	6.08	1.3724	0.201
Toluene	2.38	1.4864	0.0164
$CHCl_3$	4.81	1.4382	0.151
Acetone	20.7	1.3592	0.284
MeCN	37.5	1.3421	0.306
MeOH	33.0	1.3284	0.309

Section 4: Computation

Density functional theory (DFT) calculations were performed with Spartan computational software using B3LYP density functional and 6-31G* basis set.

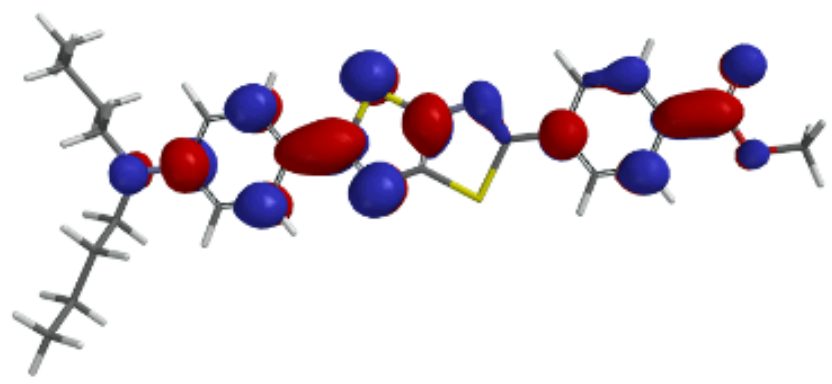

LUMO +1
-1.0 eV

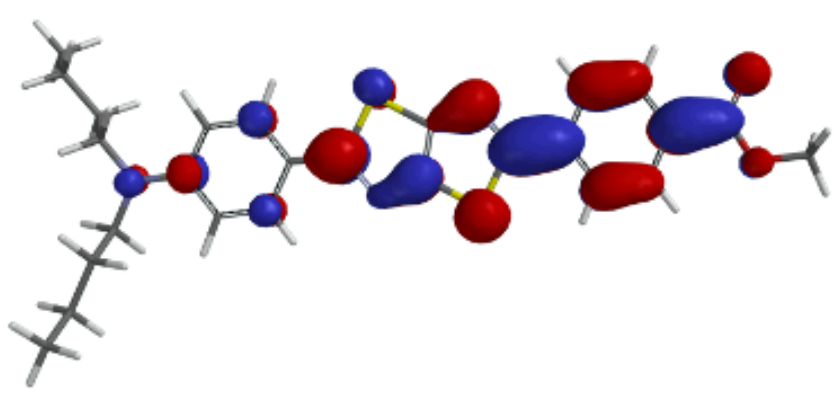

LUMO
-2.0 eV

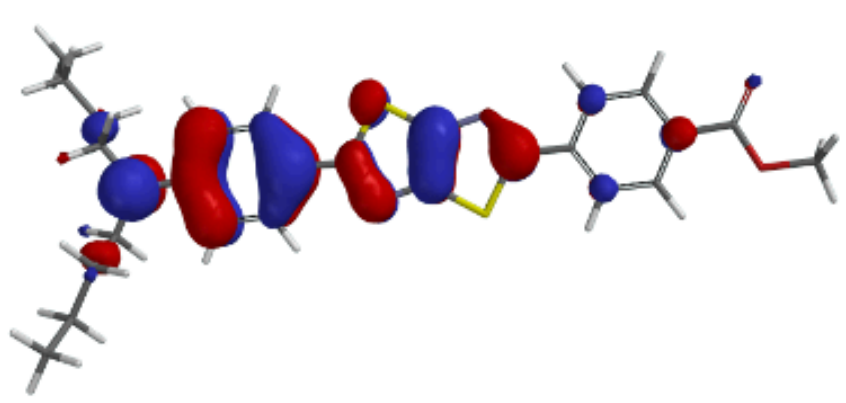

HOMO
-5.0 eV

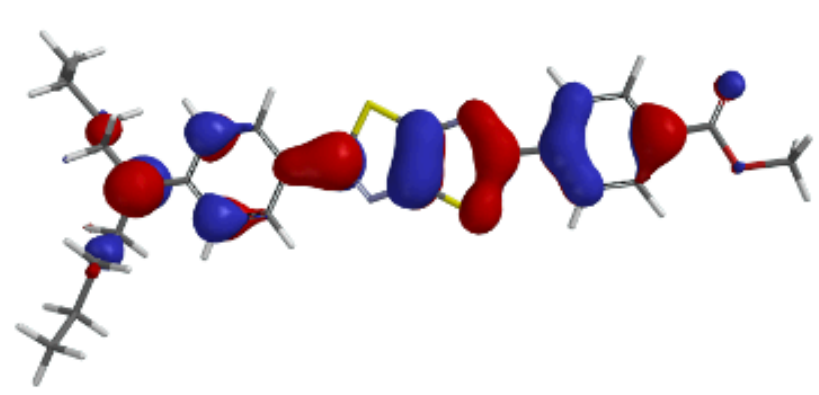

HOMO -1
-6.1 eV

Figure S32: Ground state optimization with LUMO+1, LUMO, HOMO, and HOMO-1 of TwistTz-M in vacuum.

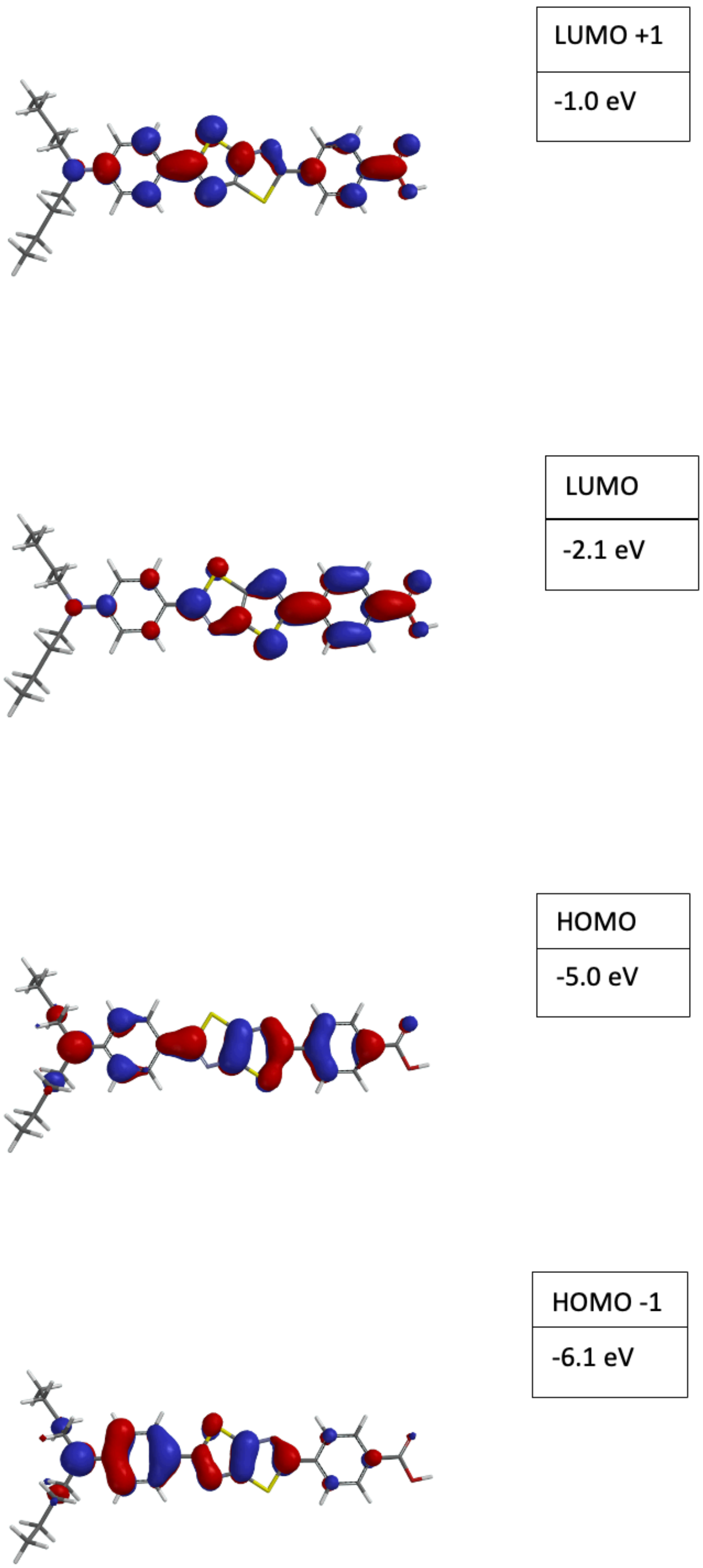

Figure S33: Ground state optimization with LUMO+1, LUMO, HOMO and HOMO-1 of TwistTz-A in vacuum.

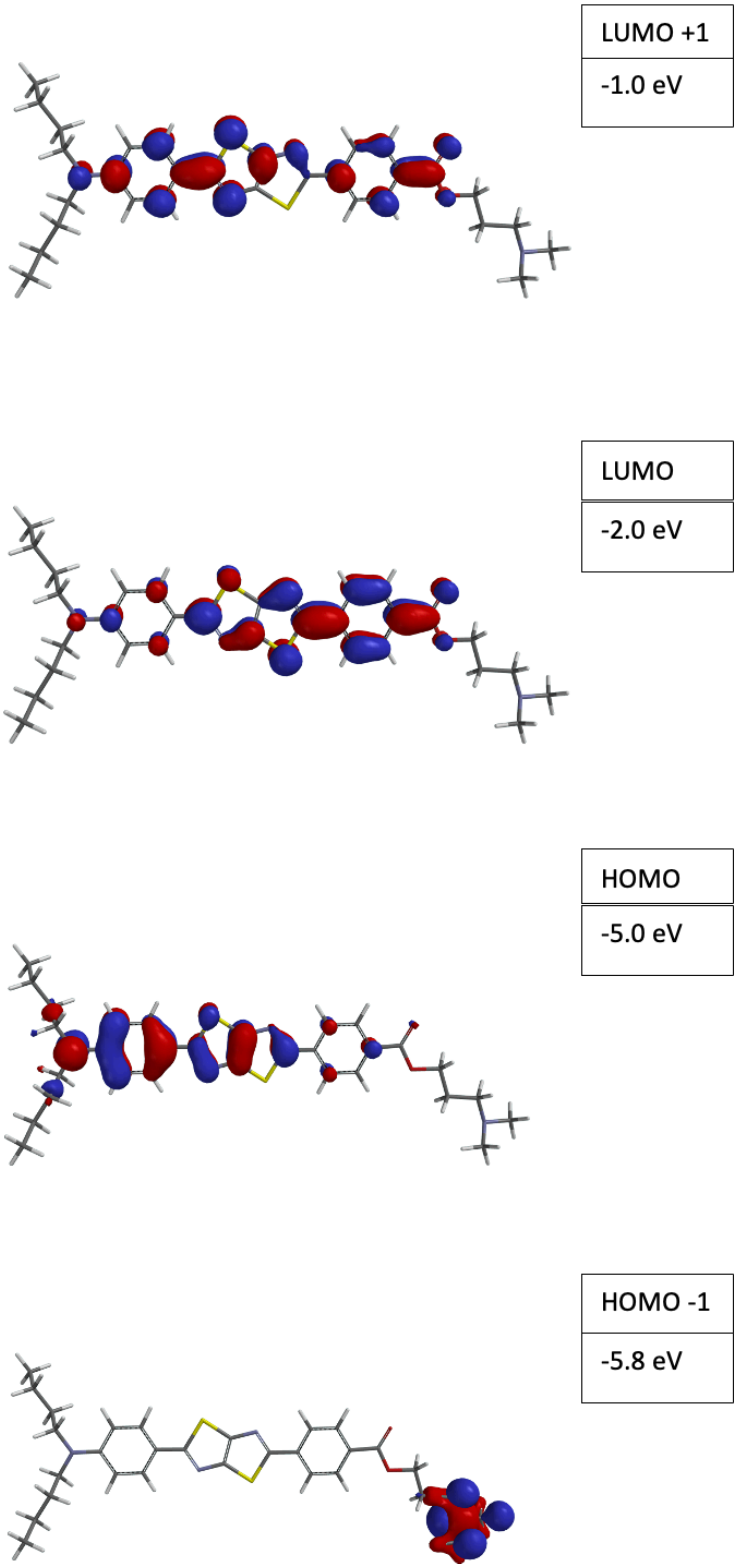

Figure S34: Ground state optimization with LUMO+1, LUMO, HOMO and HOMO-1 of TwistTz-P in vacuum (one methyl group removed from N for accurate results).

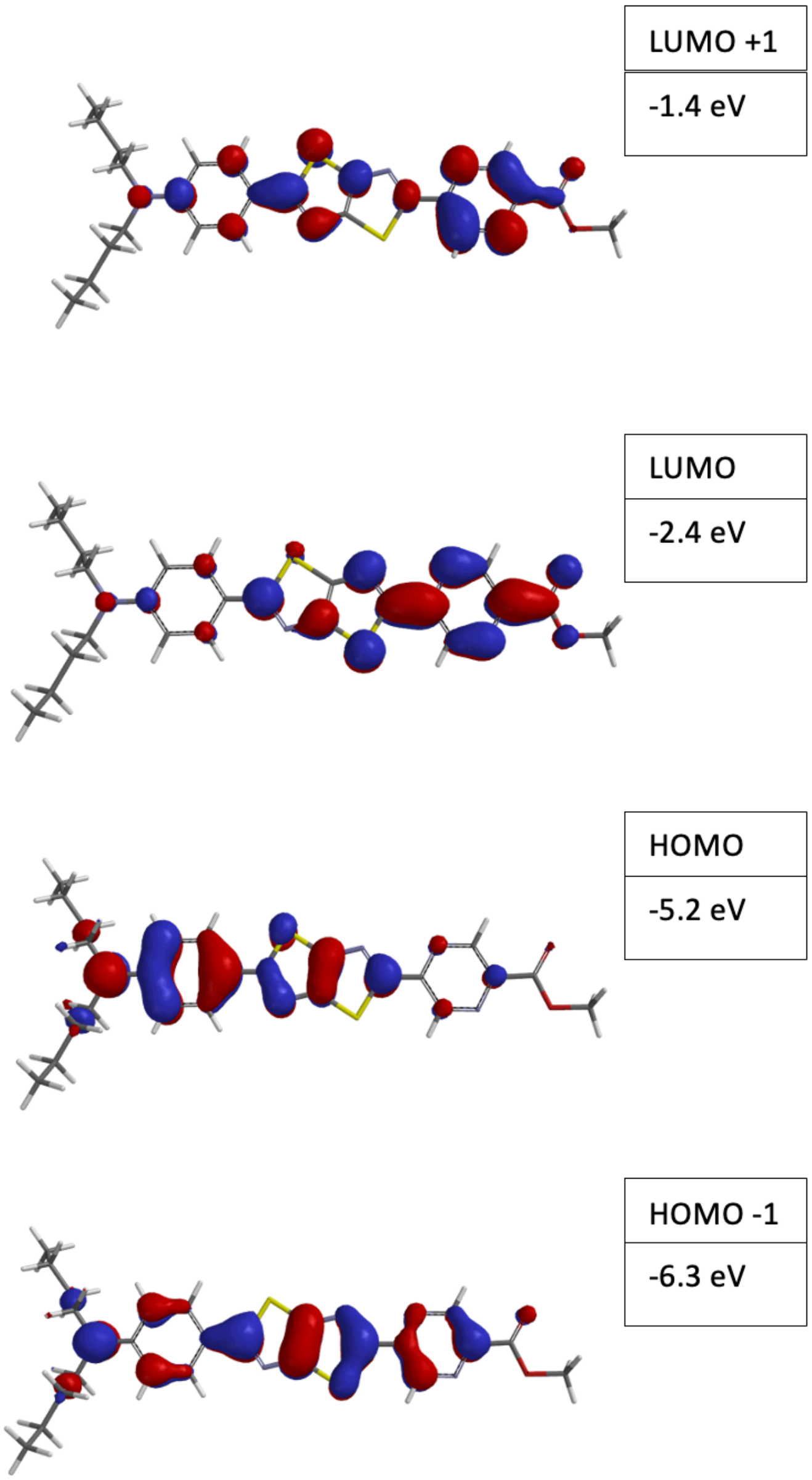

Figure S35: Ground state optimization with LUMO+1, LUMO, HOMO, and HOMO-1 of FlatTz-M in vacuum.

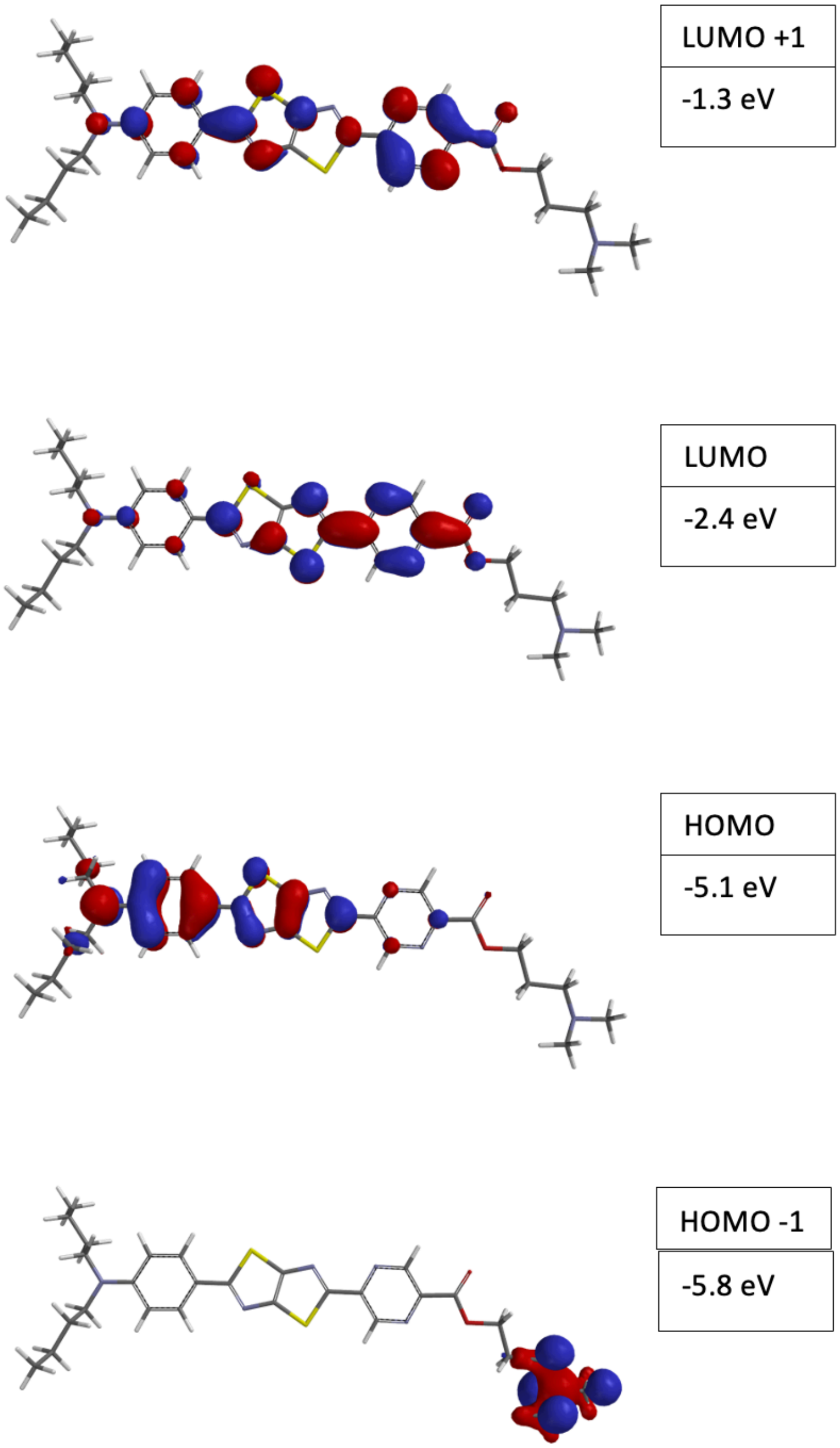

Figure S36: Ground state optimization with LUMO+1, LUMO, HOMO, and HOMO-1 of FlatTz-P in vacuum (one methyl group removed from N for accurate results).

Section 5: Emission of Planar and Twisted States

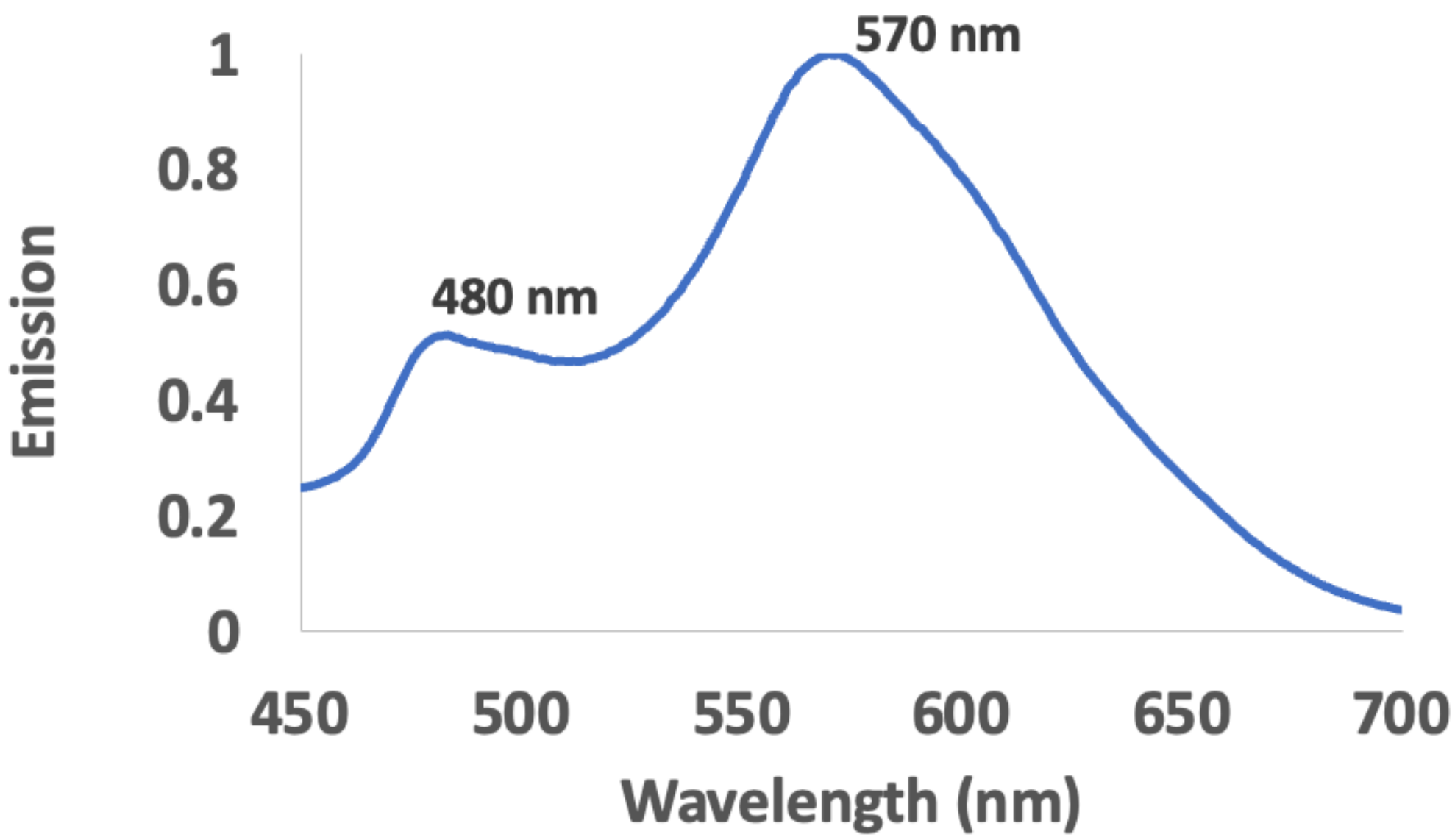

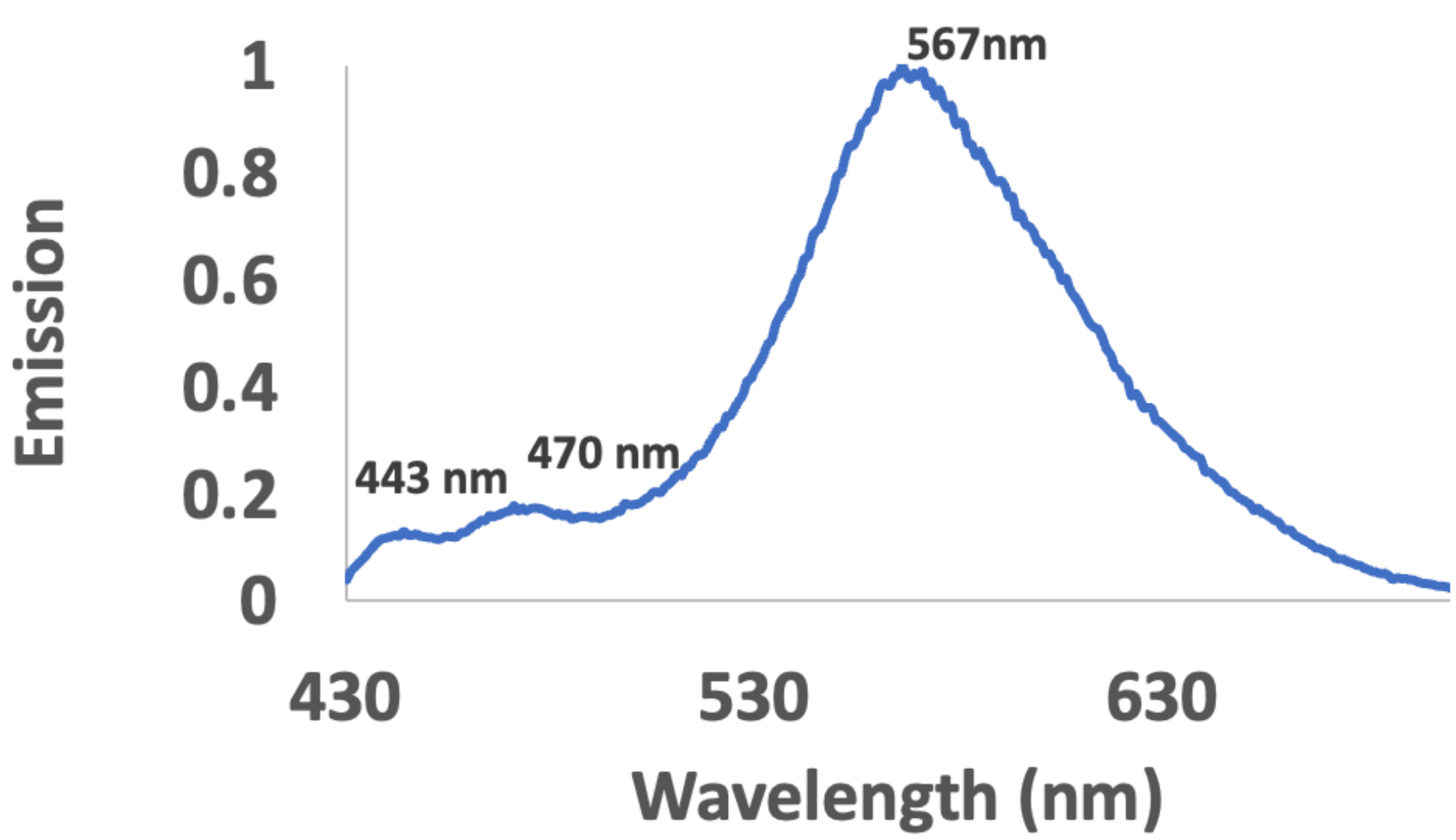

Figure S37: Emission of the planar and twisted states of TwistTz-M in DMSO (above) and TwistTz-P in MeCN (below).

Section 6: Emission Studies in Cells

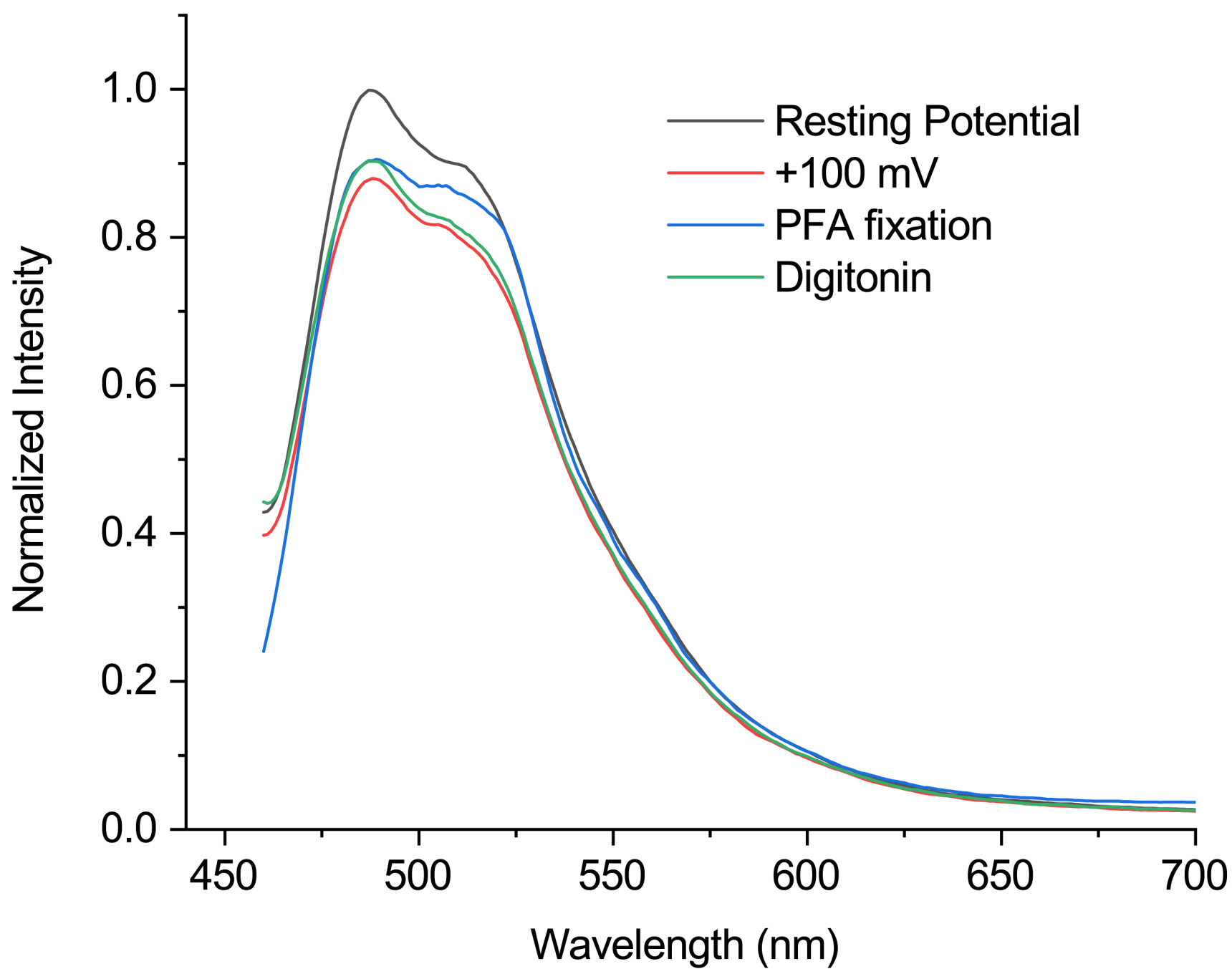

Figure S38: TwistTz-P in HEK293T cells under resting potential, +100mV, PFA fixation and Digitonin

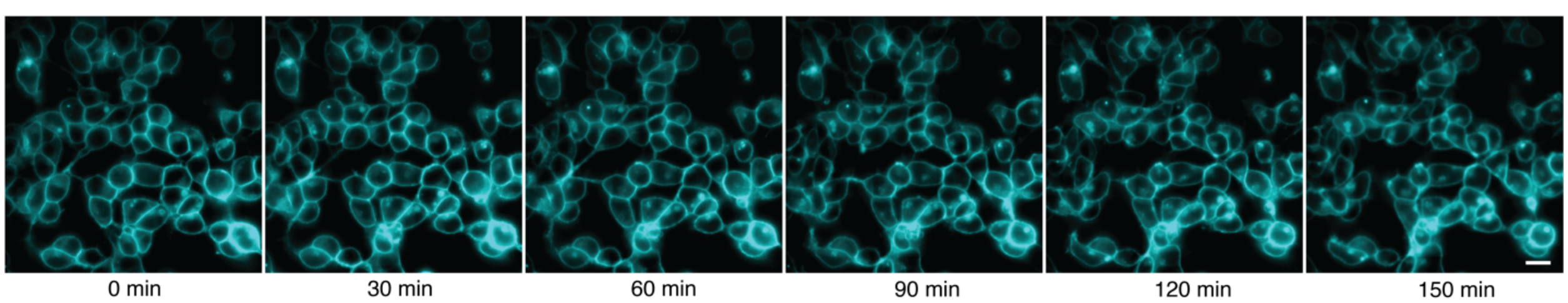

Figure S39: FlatTz-P in HEK293T cells imaged over a period of 150 min.

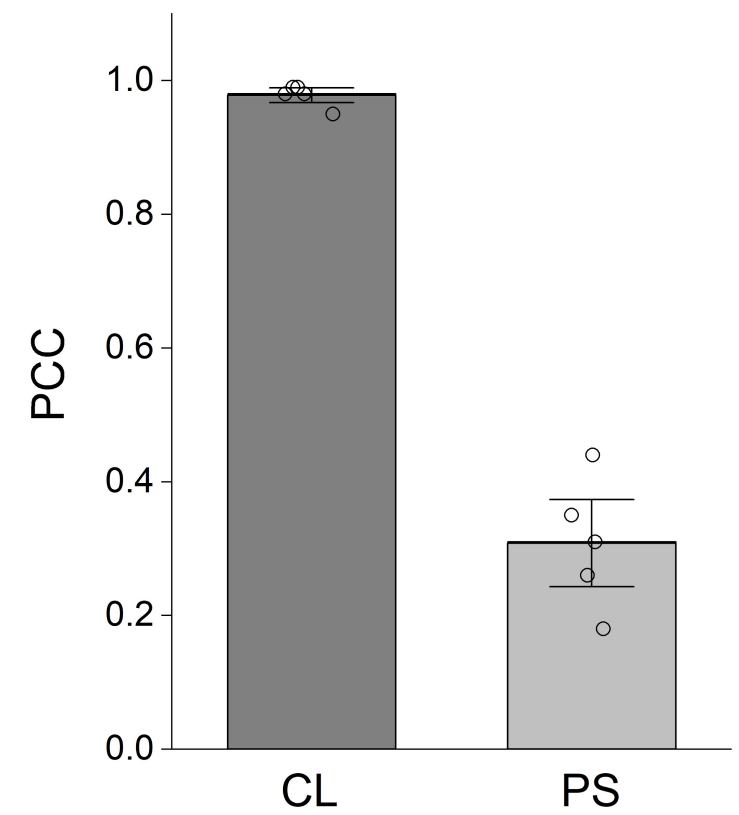

Figure S40: TwistTz-P Pearson Correlation Coefficient (PCC) and pixel shifted PCC

Section 7: Molecular Angles with Applied Voltage

Section 7.1. Angles for TwistTz-P and FlatTz-P in excited and ground states

(Here, E-field stands for applied electric field in atomic units (corresponding voltage - mV), D-STTz stands for the angle between the phenyl ring of the donor group and the S of the TTz core, D-NTTz stands for the angle between the phenyl ring of the donor group and the N of the TTz core, STTz-A stands for the angle between the phenyl ring of the acceptor group and the S of the TTz core, and NTTz-A stands for the angle between the phenyl ring of the acceptor group and the N of the TTz core.)

Table S3 (above) and Table S4 (below). Angles for TwistTz-P in excited states (above – Table S3) and ground states (below – Table S4)

Excited States	E-field (Corresponding Voltage)	D-STTz	D-NTTz	STTz-A	NTTz-A
	0.0001a.u. (-100 mV)	12.8 º	11.1 º	0.6 º	0.5 º
	0.00005 a.u. (-50 mV)	16.2 º	14.0 º	-1.9 º	-1.7 º
	0.00001a.u. (-10 mV)	17.8 º	15.4 º	-2.4 º	-2.2 º
	-0.00001a.u. (+10 mV)	18.6 º	16.2 º	-2.8 º	-2.6 º
	-0.00005 a.u. (+50 mV)	28.8 º	25.6 º	-7.2 º	-6.5 º
	-0.0001a.u. (+100 mV)	30.8 º	27.6 º	-7.9 º	-7.1 º

Ground States	E-field (Corresponding Voltage)	D-STTz	D-NTTz	STTz-A	NTTz-A
	0.0001a.u. (-100 mV)	0.7 º	0.7 º	0.7 º	0.6 º
	0.00005 a.u. (-50 mV)	0.6 º	0.7 º	0.7 º	0.6 º
	0.00001a.u. (-10 mV)	0.6 º	0.6 º	0.6 º	0.5 º
	-0.00001a.u. (+10 mV)	0.6 º	0.6 º	0.6 º	0.5 º
	-0.00005 a.u. (+50 mV)	0.5 º	0.6 º	0.5 º	0.5 º
	-0.0001a.u. (+100 mV)	0.5 º	0.5 º	0.5 º	0.4 º

Table S5 (above) and Table S6 (below). Angles for FlatTz-P in excited states (above – Table S5) and ground states (below – Table S6)

Excited States	E-field (Corresponding Voltage)	D-STTz	D-NTTz	STTz-A	NTTz-A
	0.00005 a.u. (-50 mV)	2.6 º	2.3º	3.0 º	2.7 º
	-0.00001a.u. (+10 mV)	2.3 º	2.0 º	2.8 º	2.5 º
	-0.00005 a.u. (+50 mV)	2.1 º	1.8 º	2.6 º	2.3 º
	-0.0001a.u. (+100 mV)	2.2 º	1.8 º	2.9 º	1.6 º

Ground States	E-field (Corresponding Voltage)	D-STTz	D-NTTz	STTz-A	NTTz-A
	0.0001a.u. (-100 mV)	1.0 º	0.8 º	1.3 º	1.3 º
	0.00005 a.u. (-50 mV)	1.0 º	0.8 º	1.3 º	1.3 º
	0.00001a.u. (-10 mV)	1.0 º	0.8 º	1.3 º	1.3 º
	-0.00001a.u. (+10 mV)	1.0 º	0.8 º	1.3 º	1.3 º
	-0.00005 a.u. (+50 mV)	1.0 º	0.8 º	1.3 º	1.3 º
	-0.0001a.u. (+100 mV)	1.0 º	0.9 º	1.4 º	1.3 º

Section 7.2. Angles of TwistTz-P in different solvents

(Here, in Table S5 (below), D-STTz stands for the angle between the phenyl ring of the donor group and the S of the TTz core, D-NTTz stands for the angle between the phenyl ring of the donor group and the N of the TTz core, STTz-A stands for the angle between the phenyl ring of

the acceptor group and the S of the TTz core, and NTTz-A stands for the angle between the phenyl ring of the acceptor group and the N of the TTz core.)

Table S7. Angles of TwistTz-P in different solvents

	D-NTTz	D-STTz	NTTz-A	STTz-A
Toluene	0.8 º	0.9 º	0.9 º	1.1 º
$CHCl_3$	0.3 º	0.3 º	1.0 º	1.1 º
DCM	0.2 º	0.2 º	1.2 º	1.4 º
THF	0.3 º	0.2 º	1.1 º	1.3 º
Acetone	0.1 º	0.1 º	1.3 º	1.4 º
DMSO	0.1 º	0.0 º	1.3 º	1.5 º
MeCN	0.1 º	0.1 º	1.3 º	1.5 º
MeOH	0.1 º	0.1 º	1.3 º	1.5 º

Section 7.3. Molecular Angles with Applied Voltage

Using the gas phase geometries (B3LYP functional/6-31G* basis set) of TwistTz-P from Spartan, we first aligned the XYZ file along the z-axis (in preparation for applying a uniform electric field) and re-optimized the structure in the gas phase using B3LYP/def2-SVP in ORCA 5.0.2.

We used solvatochromatism to benchmark the performance of TDDFT on capturing trends in fluorescence properties. For solvatochromism, we re-optimized the gas phase structure in the presence of continuum dielectric (CPCM) where each solvent was assigned its corresponding dielectric constant. After ground state geometry optimization, we then carried out excited state geometry optimization in CPCM following the first excited state under TDDFT with the Tamm-Dancoff approximation (TDA). Benchmark studies were performed with increasingly large basis set size (def2-SVP, def2-TZVP, and def2-TZVPP). Fluorescence properties from the first excited state were compared to experimental measurements and found to correlate well with

experimentally measured fluorescence wavelengths, independent of basis set size (**Figure S27, S41**).

Upon identifying that TDDFT computed trends were preserved with respect to experimental data, we next optimized the TwistTz-P molecule in the excited state under the presence of electric field (also using B3LYP/def2-SVP in the gas phase with ORCA 5.0.2). The magnitude of the electric field (in atomic units) was computed as follows (example shown assuming 100 mV voltage and a 4 nm thick bilayer):

$$100\ mV = 0.1\ V * \frac{1\ a.u.}{27.2113\ V} = 0.0037\ a.u.$$

The TwistTz-P molecule resides in 1 leaflet of the bilayer. Correspondingly, it experiences the field over half of the bilayer thickness (thus 2 nm).

$$4\ nm\ bilayer = 2\ nm * \frac{10^{-9}m}{1\ nm} * \frac{1\ a.u.}{5.29 * 10^{-11}m} = 37.8\ a.u.$$

The electric field is defined as follows:

$$E = -\nabla V \approx -\frac{V}{d} = \frac{-0.0037\ a.u.}{37.8\ a.u.} = -\ 0.0001\ a.u.$$

Therefore, the following E-fields are used for excited state optimization:

Table S8. Transmembrane potential corresponding to each electric field

e field	transmembrane potential
0.0001 a.u.	-100 mV
0.00005 a.u.	-50 mV
0.00001 a.u.	-10 mV
-0.00001 a.u.	10 mV
-0.00005 a.u.	50 mV
-0.0001 a.u.	100 mV

Geometries optimized in the first excited state under electric fields were then analyzed using their corresponding dihedral angles.

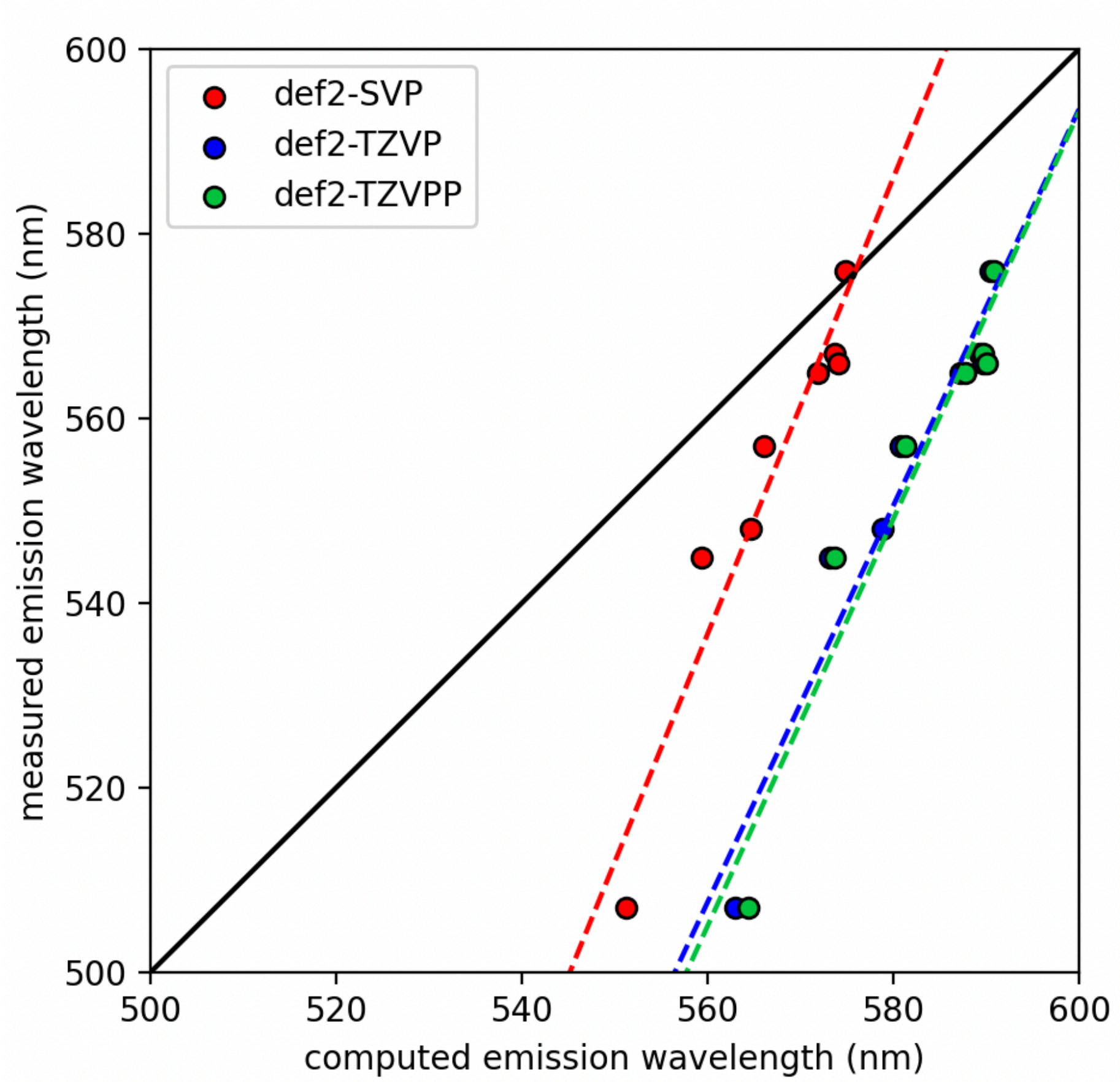

Figure S41. Computed emission wavelength (nm) compared to the measured emission wavelength (nm) for TwistTz-P as a function of increasing basis set size from def2-SVP (red), to def2-TZVP (blue), and def2-TZVPP (green). Each dot represents TwistTz-P in a different solvent with a different dielectric constant.

Table S9. Relative oscillator strengths of TwistTz-P (above) and FlatTz-P (below) corresponding to different electric fields*

Electric field	Relative oscillator strength
0.0001 a.u.	0.698413
0.00005 a.u.	0.669298
0.00001 a.u.	0.652482
-0.00001 a.u.	0.642520
-0.00005 a.u.	0.545027
-0.0001 a.u.	0.517214

Electric field	Relative oscillator strength
0.00005 a.u.	0.522993
-0.00001 a.u.	0.524471
-0.00005 a.u.	0.525473
-0.0001 a.u.	0.526823

* w/o considering membrane or solvents (data should be treated qualitatively)
